# Nonalternant acenaphtho[1,2-*k*]fluoranthene diimide derivatives with outstanding optical-limiting performance

**DOI:** 10.1039/d6sc05845k

**Published:** 2026-09-17

**Authors:** Jiaxin Lan, Zhipeng Ma, Wentao Liu, Shuhao Zeng, Guangxiong Bin, Longbin Ren, Liping Zheng, Chenghua Sun, Jibin Sun, Huajie Chen

**Affiliations:** a Key Laboratory of Environmentally Friendly Chemistry and Application of Ministry of Education, College of Chemistry, Xiangtan University Xiangtan 411105 P. R. China chenhjoe@xtu.edu.cn zhenglp@xtu.edu.cn; b Key Laboratory of Photochemical Conversion and Optoelectronic Materials, Technical Institute of Physics and Chemistry, Chinese Academy of Sciences Beijing 100190 P. R. China sunjibin@mail.ipc.ac.cn; c Beijing National Laboratory for Molecular Sciences, Organic Solids Laboratory, Institute of Chemistry, Chinese Academy of Sciences Beijing 100190 P. R. China

## Abstract

We report the synthesis and characterization of a series of nonalternant acenaphtho[1,2-*k*]fluoranthene diimide derivatives (L1–L4) featuring different aryl substituents and for the first time examine their reverse saturable absorption (RSA) properties and optical-limiting (OL) application potentials. The combined benefits of a large nonalternant π-framework and unique donor–acceptor type conformation make it possible to improve their chemical stability and intramolecular charge-transfer interaction, offering excellent feasibility of finely regulating their OL behavior based on the RSA mechanism. Z-scan experiments uncover that all compounds exhibit outstanding OL performance, far superior to most reported classical OL materials (*e.g.*, C_60_). By applying 532 nm nanosecond laser pulses, the thiophene-bearing L1 achieves the lowest OL onset threshold of 7 mJ cm^−2^, the lowest OL threshold of 0.123 J cm^−2^, and the largest nonlinear absorption coefficient of 941 cm GW^−1^, all of which significantly outweigh those of benchmark C_60_. The RSA mechanism of L1–L4 is well identified by time-resolved transient absorption spectroscopy. The stronger excited-state absorption and longer excited-state lifetimes of L1, L2, and L4 contribute to higher OL performance compared to L3. Our findings demonstrate the effectiveness of imide-functionalized nonalternant polycyclic aromatic hydrocarbons towards constructing high-performance OL materials.

## Abstract 2

Four nonalternant acenaphtho[1,2-*k*]fluoranthene diimide derivatives are prepared for optical-limiting (OL) applications. These diimides exhibit strong reverse saturable absorption and outstanding OL performance.

## Graphical abstract

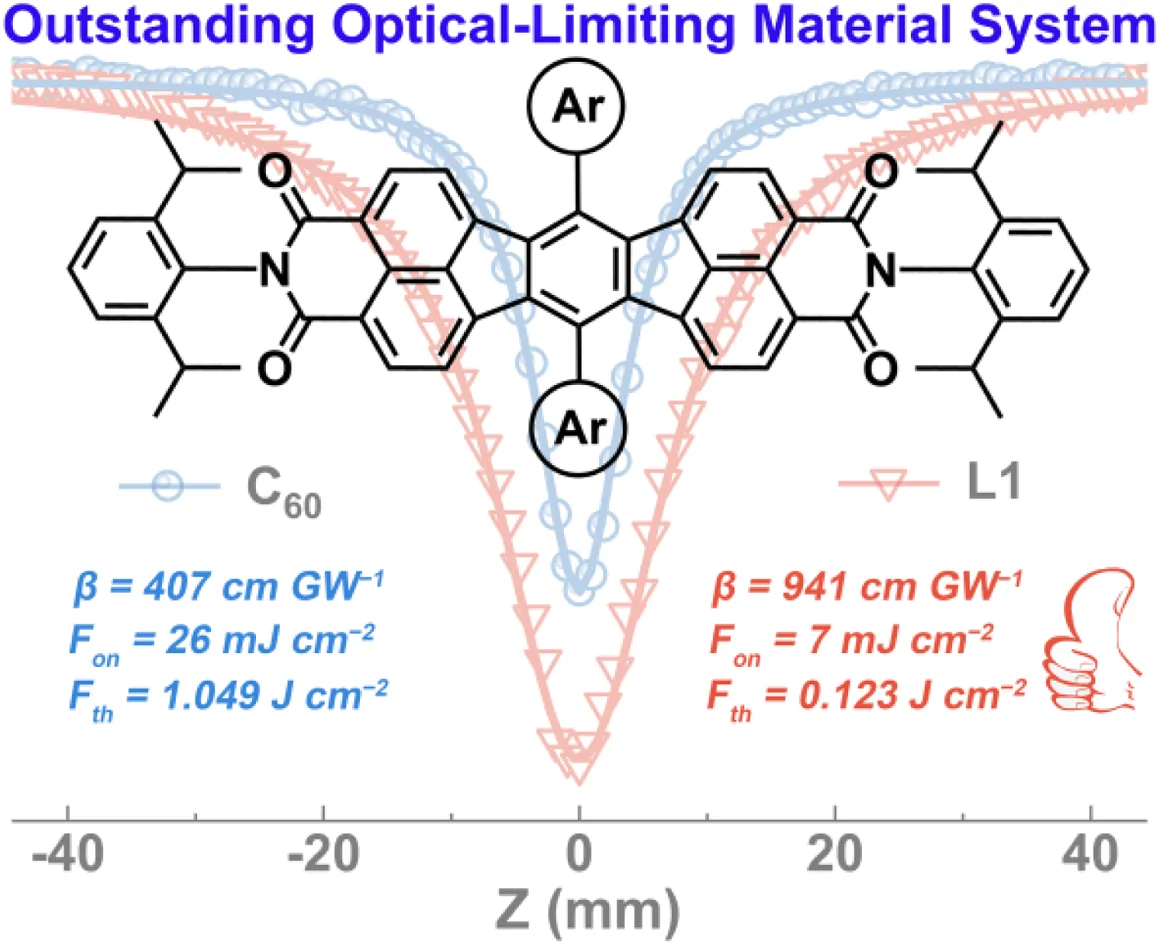

## Introduction

Optical-limiting (OL) materials have been attracting widespread attention during the past few decades due to their promising application potentials in the laser protection technologies applied in both military and civilian fields.^1–4^ As a typical case of nonlinear optical (NLO) responses, OL materials exhibit high transmittance against weak-intensity laser pulses while low transmittance to intense ones, which is useful for smart protection of optical instruments and human eyes from laser-induced damage.^1,3,4^ The intensively studied OL materials mainly can be categorized into carbon-based materials (*e.g.*, fullerenes,^5,6^ carbon nanotubes,^7,8^ and graphene^1,4,9^), organic π-conjugated molecules (OCMs, *e.g.*, porphyrins,^7,10,11^ phthalocyanines,^12,13^ and donor–acceptor (D–A) type π-systems^14–16^), and π-conjugated porous frameworks (*e.g.*, metal–organic frameworks^17,18^ and covalent-organic frameworks^19,20^). Among them, OCMs have been a popular topic of this research area owing to their unique benefits, *e.g.*, good solubility, diverse structures, and tailorable optoelectronic properties. It has been widely accepted that OCMs display OL responses mainly dependent on the mechanism of reverse saturable absorption (RSA),^2,15,16,21^ which takes place when the materials have stronger excited-state absorption (ESA) than that of the ground state. Considering that the ESA of OCMs is mainly determined by their chemical structures and molecular orbital energy levels,^2,15,21–25^ rational design and synthesis of π-conjugated systems with strong ESA character is thus crucial for the development of high-performance optical-limiting OCMs in order to realize a high nonlinear absorption coefficient (*β*), a low OL onset threshold (*F*_on,_ defined as the input laser fluence at which the normalized transmittance (*T*_norm_) falls to 0.95), and a low OL threshold (*F*_th,_ defined as the input laser fluence at a *T*_norm_ value of 0.5).^2–4^

Nonalternant cyclopenta-fused polycyclic aromatic hydrocarbons (CP-PAHs)—a distinct family of fused-ring π-systems—have recently been extensively studied in the fields of chemistry and materials science due to their structural similarities to fullerene derivatives (*e.g.*, C_60_), enhanced electronic tunability, and outstanding ability to accept electrons.^26–34^ Incorporating pentagonal rings into the π-framework of PAHs (particularly for linear acene systems) has been proven to be an effective strategy to create more-stable CP-PAHs,^35,36^ and this design concept was successfully extended to access large-sized, stable CP-PAHs for various functional applications.^15,30,32,37–39^ On the other hand, the anti-aromatic pentagonal rings embedded in the PAHs' π-frameworks could behave as electron-deficient subunits,^40^ which can be expected to modulate frontier orbital energy levels and reduce band gaps induced by unique intramolecular D–A interactions.^30,38,40,41^ Although these characteristics make CP-PAHs favorable for OL materials, to the best of our knowledge, very few examples have been documented so far in the literature.^15,23,42,43^ Early in 2023, our group demonstrated that triptycene end-capped N-doped nonalternant nanoribbons—a novel family of N-doped CP-PAHs—exhibit strong ESA characteristics and a remarkable OL response.^15^ Upon applying a 532 nm laser pulse, the nanoribbon NNNR-2 (Fig. 1) featuring a 13 linearly fused-ring backbone shows the *F*_on_, *F*_th_, and *β* values of 13 mJ cm^−2^, *ca*. 0.4 J cm^−2^, and 374 cm GW^−1^, which, respectively, are far superior to those of the well-known C_60_ (*F*_on_ = 43 mJ cm^−2^, *F*_th_ > 3 J cm^−2^, *β* = 153 cm GW^−1^) under the same measurement conditions. Recently, Song and Xiao *et al.* reported two helicene-like CP-PAHs featuring a highly extended π-conjugation, which contributes to a red-shifted light-capturing band, broadband ESA, and a good OL response in the near-infrared region.^23^ Particularly the double helicene-like CPPAH-2 (Fig. 1) showcases the *F*_on_ values of 6.2 and 20.8 mJ cm^−2^ when treated with femtosecond pulse lasers of 800 and 1030 nm, respectively. Despite this important progress in accessing such materials, exploration of CP-PAHs for OL applications is still a less tapped area compared to the classical OL material systems (*e.g.*, fullerenes, graphene, phthalocyanines, and porphyrins).^1–4^ To further boost OL performance (*e.g.*, achieving a higher *β* value but lower *F*_on_ and *F*_th_ values) and understand materials' structure–performance relationship in depth, exploiting novel design strategies and developing novel CP-PAH π-systems are thus highly desirable.

**Fig. 1:**
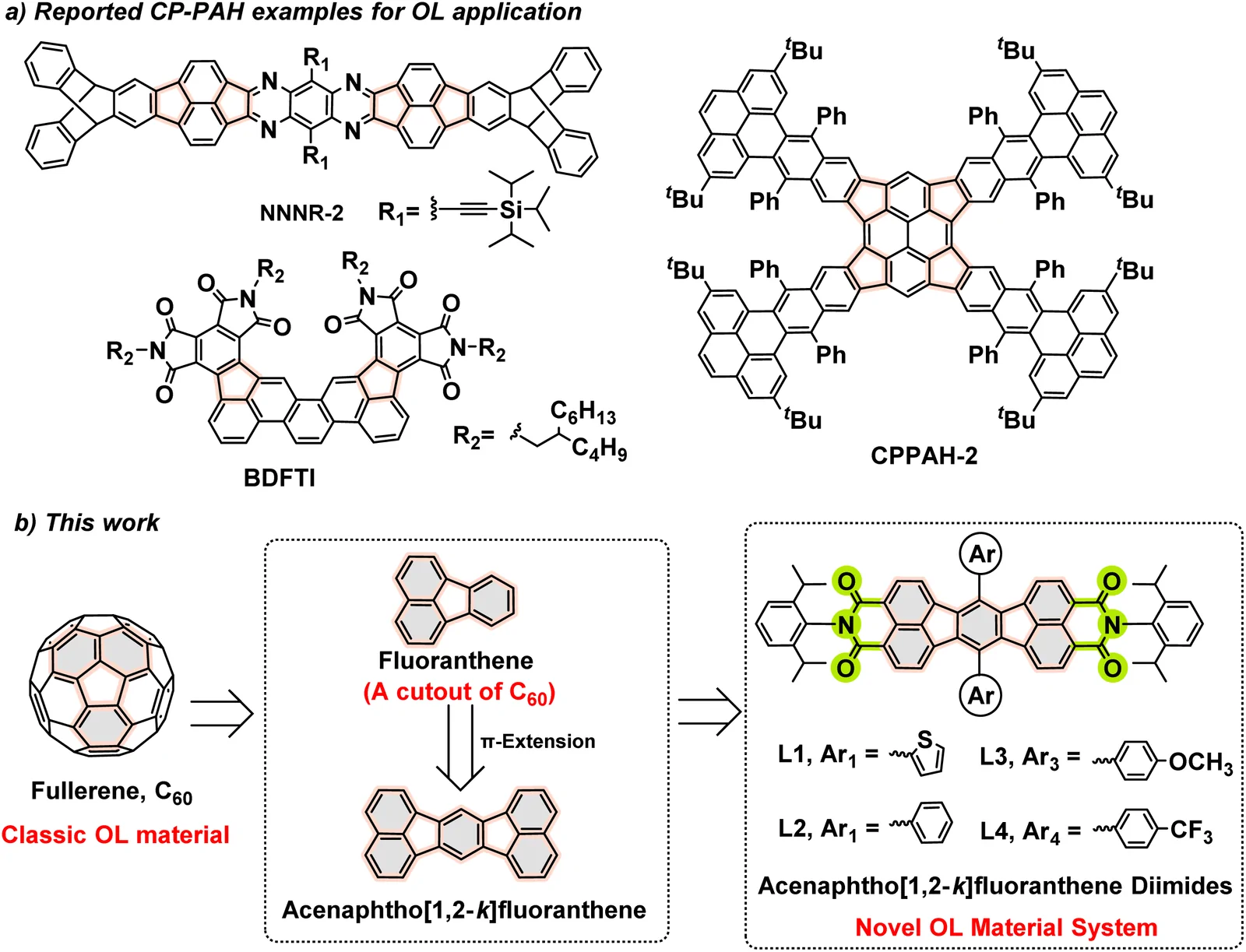
(a) Reported representative examples of CP-PAHs for OL applications. (b) Molecular design strategy for D–A type acenaphtho[1,2-*k*]fluoranthene diimides bearing different aryl substituents (L1–L4) for OL applications studied in this work.

Derived as a molecular cutout of fullerene C_60_, fluoranthene is a typical CP-PAH that can be regarded as a benzene annulation of a naphthalene moiety in a pentagon mode (Fig. 1). The presence of an electron-poor pentagon ring in the fluoranthene's π-backbone contributes to the formation of a unique intramolecular charge-transfer (ICT) interaction and anomalous fluorescence properties, making it highly promising for structural derivatives and functional regulations in the fields of both photochemistry and materials.^44–46^ The chemical modification of fluoranthene, particularly through the extension of π-conjugation or the introduction of imide units,^37,43,47–52^ has been proven effective in the enhancement of charge-carrier mobility, photovoltaic efficiency, ICT interaction, and chemical stability. These attractive characteristics have drawn intensive attention in building fluoranthene-derived CP-PAHs for a wide range of applications like n-type organic field-effect transistors (OFETs),^37,52^ non-fullerene solar cells (NFSCs),^48,49^ and organic light-emitting diodes.^44^ For example, Pei and co-workers reported a typical example of fluoranthene-derived CP-PAH diimides, *i.e.*, 2-octyldodecyl-substituted acenaphtho[1,2-*k*]fluoranthene diimide, and demonstrated a photovoltaic efficiency of 0.33% when paired with an electron-donating material (*i.e.*, poly(3-hexylthiophene)) in NFSC devices.^48^ Despite this important progress in organic electronics, the application of such material systems, especially for the imide-functionalized CP-PAHs, in the OL field remains rarely documented.^15,23,42,43^ Recently, our group reported the first evaluation of the OL performance of an imide-bearing CP-PAH system (*i.e.*, benzodifluoranthene tetraimides, BDFTI, Fig. 1), which was synthesized through UV-photocyclization of a vinyl-bridged difluoranthenediimide precursor.^43^ Both RSA and OL responses of BDFTI are clearly observed, while its OL performance is highly unsatisfactory compared to the other material systems (*i.e.*, C_60_) reported in the literature (Fig. 6 and Table S7). To examine the feasibility of imide-bearing CP-PAHs for high-performance OL applications, further development of such π-scaffolds is worthy of study.

In this contribution, we report the synthesis and characterization of a series of nonalternant acenaphtho[1,2-*k*]fluoranthene diimide derivatives (L1–L4, Fig. 1) bearing different aryl substituents and for the first time evaluate their application potentials in the OL field. To access high-performance OL materials, our design inspiration initially stems from the unique nonalternant π-system of C_60_ (Fig. 1), a well-known OL material that is heavily employed as a standard to evaluate the OL performance of novel material systems. As illustrated in Fig. 1, the parent core, acenaphtho[1,2-*k*]fluoranthene, can be regarded as a π-extended molecular cutout of C_60_ or a fluoranthene-derived CP-PAH. Such a unique π-backbone is extremely important for facilitating an OL response based on the RSA mechanism, thereby resulting in high OL performance for C_60_, and potentially for its derivatives (*e.g.*, L1–L4). Another key benefit of our molecular design is that incorporating two electron-poor imide end-caps into L1–L4 makes it possible to form a unique D–A type nonalternant π-system. This structural design can be expected to further improve their chemical stability and ICT interaction and also offers excellent feasibility of modulating their ESA and RSA behaviors and, in turn, their OL response induced by the combined benefits of large nonalternant π-backbones and strong ICT interactions. Strategic installation of different aryl substituents (*i.e.*, thienyl, phenyl, methoxyphenyl, and trifluoromethylphenyl) provides the possibility to finely regulate their electronic structures and optoelectronic properties, such as RSA and OL behaviors. As expected, the open-aperture Z-scan experiments reveal that L1–L4 all exhibit strong RSA behavior and outstanding OL performance upon treatment with 532 nm nanosecond laser pulses. Among them, the thiophene-substituted L1 achieves the best OL performance, exhibiting the lowest *F*_on_ and *F*_th_ values (7 mJ cm^−2^ and 0.123 J cm^−2^, respectively) and the largest *β* value (941 cm GW^−1^). For such material systems, low *F*_on_ and *F*_th_ values coupled with a high *β* value fully demonstrate outstanding OL performance, significantly surpassing those of the well-known OL material C_60_ (*F*_on_ = 26 mJ cm^−2^, *F*_th_ = 1.049 J cm^−2^ and *β* = 407 cm GW^−1^) and many classical OL materials reported in the literature (Fig. 6 and Table S7). This work presents an efficient strategy towards synthesizing high-performance OL materials, highlighting the imide-functionalized CP-PAHs as an outstanding material system for future nonlinear optics applications, particularly in laser protection fields.

## Results and discussion

Scheme 1 shows the synthetic routes of L1–L4 featuring different aryl substituents. The key starting materials, compounds 1 and 2, were prepared using a known procedure.^53^ Four key ketone-bearing intermediate compounds, including 1,3-di(thiophen-2-yl)propan-2-one (5a),^49^ 1,3-diphenylpropan-2-one (5b),^54^ 1,3-bis(4-methoxyphenyl)-propan-2-one (5c),^55^ and 1,3-bis(4-(trifluoromethyl)phenyl)propan-2-one (5d),^56^ were also prepared following the corresponding literature procedures. For preparing the acenaphthylene imide motif (3), dehydrogenation oxidation of the saturated fused-pentagon ring in compound 2 was readily conducted using a low-temperature one-pot methodology developed by our group. Compound 2 was reacted with 3.0 equivalents of lithium diisopropylamide (LDA) at low temperatures ranging from −78 to −40 °C, followed by adding 3.0 equivalents of I_2_ at −78 °C, and then stirred at room temperature overnight. This reaction procedure affords the target compound 3 in 40% yield, offering excellent feasibility of recovering the residual compound 2 by column chromatography. Under the catalysis of 4-methylbenzenesulfonic acid at 110 °C, Knoevenagel condensations between compound 4 and each ketone-bearing intermediate (*i.e.*, 5a–5d) in toluene and acetic acid were accomplished in 12 h, giving ring-closure molecules, 6a–6d, in acceptable yields of 40–60%. Finally, Diels–Alder condensations between compounds 3 and 6 were carried out at 180 °C, affording the final products in isolated yields of 65% for L1, 76% for L2, 81% for L3 and 52% for L4. The chemical structures of the new compounds were elucidated by nuclear magnetic resonance analysis and high-resolution mass spectrometry. The final products L1–L4 were also characterized by Fourier-transform infrared spectroscopy (Fig. S1), in which the vibrational peaks of the carbonyl groups (*ν*_C=O_) are detectable at around 1709–1713 and 1676–1680 cm^−1^, respectively. Strategic installation of large 2,6-diisopropylphenyl and diaryl substituents endows L1–L4 with a unique 3D configuration and good solubility in most common solvents (*e.g.*, THF, chlorinated solvents, and aromatic solvents) at room temperature. Under ambient conditions, L1–L4 are extremely stable both in solution and in the powder state due to their unique nonalternant π-conjugations and low-lying energies of the highest occupied and the lowest unoccupied molecular orbitals (HOMO and LUMO), aided by deliberate decoration of multiple electron-poor species like fused-pentagon rings and imide end-caps.

**Scheme 1:**
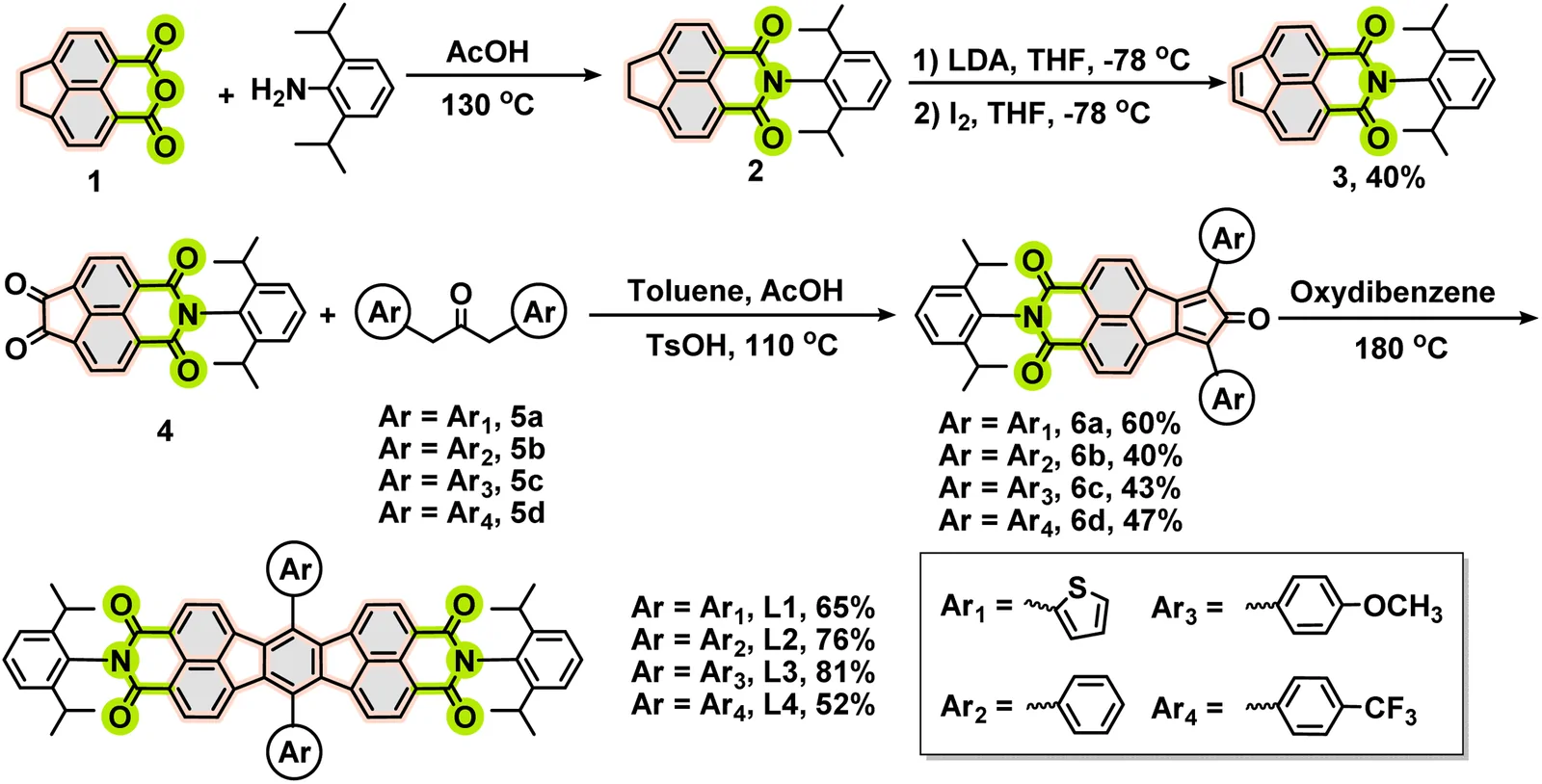
Synthetic routes to the four acenaphtho[1,2-*k*]fluoranthene diimide derivatives (L1–L4).

The chemical structures of L1–L4 were further confirmed using X-ray crystallographic analysis. All the single crystals were grown from their corresponding mixed solutions at room temperature (for the details, see the SI).^57^ As shown in Fig. 2, the single-crystal structures of L1–L4 evidence a typical 3D-shaped molecular configuration owing to the careful installations of both aryl substituents and bulky diisopropylphenyl end-caps. However, the fused-ring π-backbones of L1–L4 are proven nearly coplanar, since the twisted dihedral angles (TDAs) between the central benzene ring and its adjacent acenaphthylene subunit are less than 6.27° (Fig. S2). Such large and nearly coplanar π-backbones can be expected to facilitate effective π-electron delocalization, which is extremely important for realizing remarkable RSA properties.^15,23^ In addition, the aryl substituents installed in L1–L4 show a remarkable deviation from the π-plane of fused-ring backbones, resulting in large TDA values of 68.7 to 88.3°(Fig. 2). The TDA of L2 is significantly different from those of the other three compounds, which is due to the disorder of phenyl substituents in the single-crystal structure. The 3D-like molecular configuration coupled with multiple bulky aryl substituents suppresses effective π–π stacking between the adjacent molecules (Fig. S3), thereby resulting in loose molecular arrangements. This structural feature enables them to access better solubility compared to similar-sized, coplanar fused-ring systems.^15,39^

**Fig. 2:**
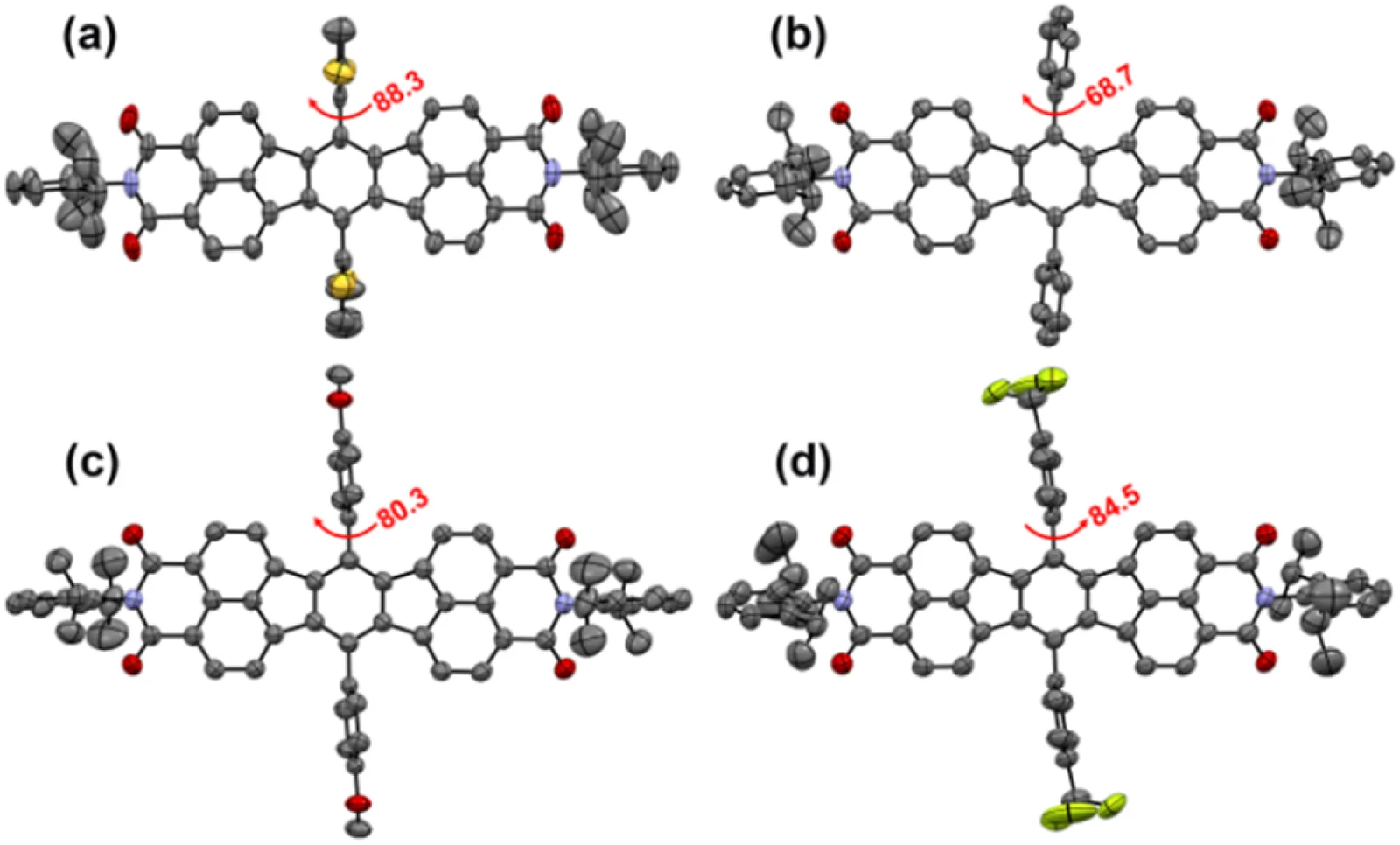
X-ray crystallographic structures of (a) L1, (b) L2, (c) L3, and (d) L4. All the hydrogen atoms are omitted for clarity. Thermal ellipsoids are plotted at the 80% probability level.

The absorption spectra of L1–L4 were investigated in chloroform and as thin films (Fig. 3a). In chloroform, four fused-ring diimides, L1–L4, all display a similar spectral shape with two distinct absorption bands located at around 300–400 and 400–570 nm, respectively. The former exhibits higher molar absorptivity compared to the latter, which is attributable to strong π–π* transition behavior. Varying diaryl substituents in L1–L4 exert a profound influence on their absorption peaks and tails in the long wavelength region. The phenyl-substituted L2 shows a low-energy absorption peak at 481 nm, exhibiting an around 6 nm red-shift relative to that of thiophenyl-substituted L1 (475 nm). Among them, compound L3 achieves the largest red-shift in absorption bands along with the low-energy absorption peak extending to 485 nm (Table S5), which can be attributed to the electron-donating 4-methoxyphenyl substituents improving ICT interaction significantly. In contrast, 4-trifluoromethylphenyl-containing L4 gives a low-energy absorption peak at 474 nm, blue-shifted by 7 and 11 nm compared to L2 and L3, respectively. This phenomenon can be attributed to the nature of weakened ICT interaction induced by electron-withdrawing trifluoromethyl groups. The absorption tails determined in the long-wavelength regions are 502 nm (L1), 508 nm (L2), 521 nm (L3), and 496 nm (L4), with the corresponding optical band gaps (*E*^opt^_g_) of 2.47, 2.44, 2.38, and 2.50 eV (Table S5), respectively. The absorption properties and spectral shapes of L1–L4 are in line with the results of time-dependent density functional theory (TD-DFT) calculations (Fig. S8–S12). The calculations indicate that the low-energy absorption peaks of L1, L2, and L4 arise from the electronic transition of HOMO → LUMO, while that of L3 can be attributed to the combined contributions of both HOMO → LUMO and HOMO-3 → LUMO transitions. In chloroform, L1–L4 exhibited weak photoluminescence (PL) behavior (Fig. S4b and S5), and the emission peaks appeared at 620, 625, 627, and 617 nm, respectively. It is obvious that their PL peaks also follow an identical tendency to their low-energy absorption peaks. We did not detect any phosphorescence signals for our samples (L1–L4) even at low temperature (78 K). The photoluminescence quantum yields of L1–L4 determined in chloroform are 6.41%, 7.56%, 7.47%, and 8.33%, respectively. The photoluminescence observed here could be assigned to the radiative transition of singlet excitons.

**Fig. 3:**
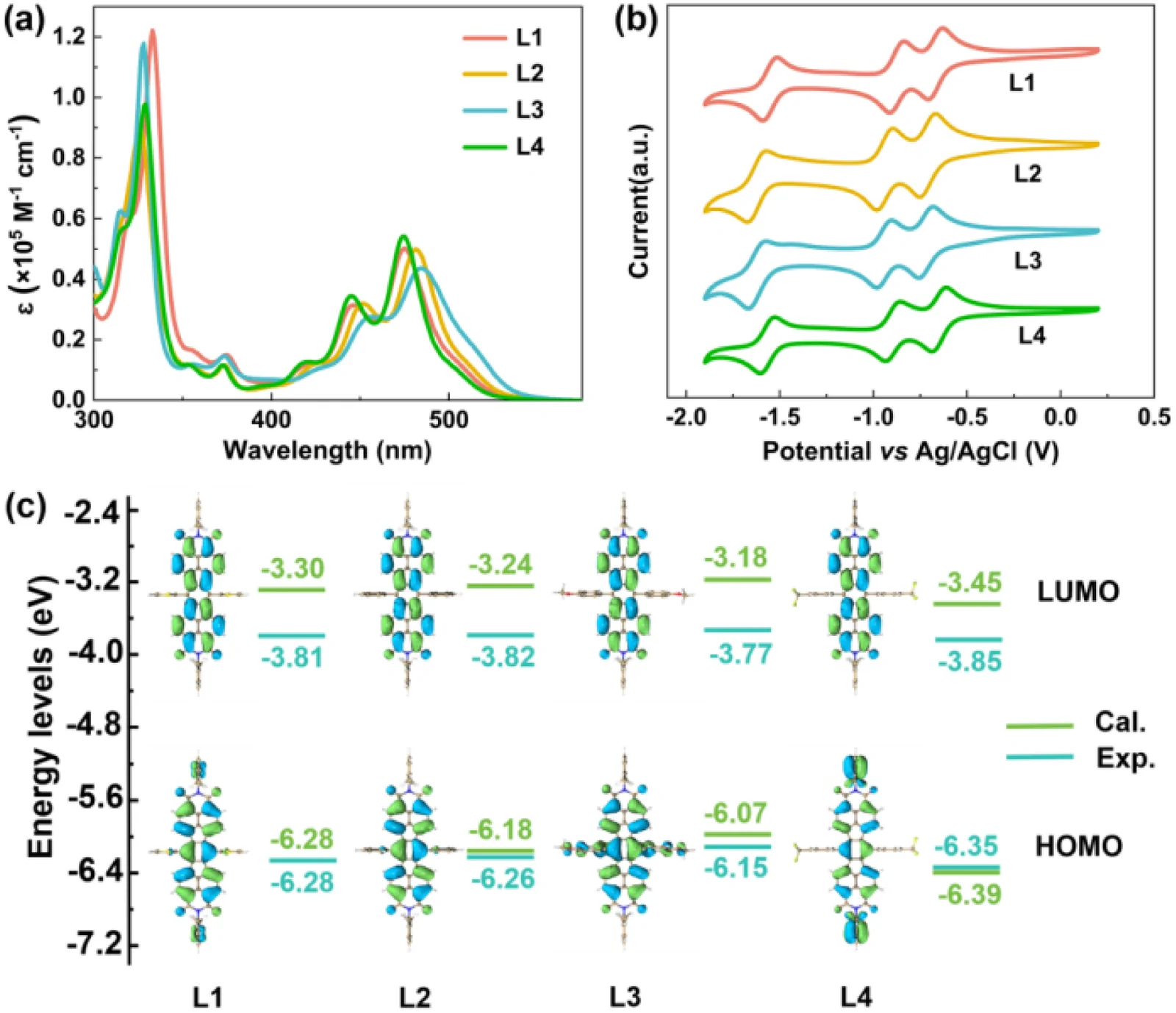
(a) Absorption spectra in chloroform. (b) Cyclic voltammetry profiles in dichloromethane. (c) DFT-optimized HOMO/LUMO orbital profiles and the energy diagrams of the HOMOs and LUMOs determined from experimental and calculated results.

To evaluate the redox behaviors and estimate the HOMO/LUMO energy levels of L1–L4, we further collected their cyclic voltammetry (CV) plots in dichloromethane (Fig. 3b). The redox potentials of L1–L4 were calibrated using the ferrocene/ferrocenium redox couple.^39^ When scanning the negative voltage window, we can observe three couples of strong and quasi-reversible electron-reduction waves for each sample, indicative of good stability of the anion species in these nonalternant fused-ring diimides.^39^ However, the redox processes of L1–L4 were proven irreversible when scanning the positive voltage window (Fig. S6). According to their redox behaviors, it can be concluded that L1–L4 are a class of highly electron-poor n-type organic semiconductors due to the incorporation of multiple electron-deficient species, *i.e.*, fused-pentagon rings and imide units. The LUMO energies of L1–L4 were estimated from the onset potential of the first reductive wave, giving the values of −3.81, −3.82, −3.77, and −3.85 eV (Table S5), respectively. The HOMO energies of L1–L4 were indirectly calculated according to the equation *E*_HOMO_ = (*E*_LUMO_ – *E*^opt^_g_) eV. The values were found to gradually decrease in the order L3 (−6.15 eV) > L2 (−6.26 eV) > L1 (−6.28 eV) > L4 (−6.35 eV). Among them, L4 achieves the lowest HOMO/LUMO energies owing to the attachment of highly electron-poor 4-trifluoromethylphenyl groups. The variation trends in both HOMO and LUMO energies were well reproduced by DFT calculations at the B3LYP-D3(BJ)/6-31G(d) level (Fig. 3c). For example, DFT-calculated HOMO values also gradually reduce in the order L3 (−6.07 eV) > L2 (−6.18 eV) > L1 (−6.28 eV) > L4 (−6.39 eV). In addition, for these materials, such low-lying HOMO and LUMO values imply excellent chemical stability against oxidation degradation under ambient conditions.^15,39^

To gain insights into the molecular geometries and electronic properties of L1–L4, density-functional theory (DFT) calculations were implemented at the B3LYP-D3(BJ)/6-31G(d) level. The calculation models of L1–L4 were simplified by replacing isopropyl groups with methyl groups (Fig. 4a). As illustrated in Fig. S7, the fused-ring π-backbones of L1–L4 are nearly coplanar. Due to steric constraints, the π-planes of the bulky aryl substituents are oriented almost perpendicular to their fused-ring π-planes. These results thus demonstrate a unique 3D-like molecular configuration for the whole molecule structure, in line with the results of single-crystal structures (Fig. 2). In addition, the plots of LUMO wavefunctions of L1–L4 are localized in a similar mode, covering the whole fused-ring π-backbones and imide units (Fig. 3c). As for their HOMO wavefunctions, the distribution characters are highly similar in the fused-ring π-backbones, while differing in their aryl substituents. Apart from L2, the other three diimides show the isosurface plot distributions not only over the fused-ring π-backbones, but also extending to the 2,6-diisopropylphenyl or 4-methoxyphenyl subunits (Fig. 3c).

**Fig. 4:**
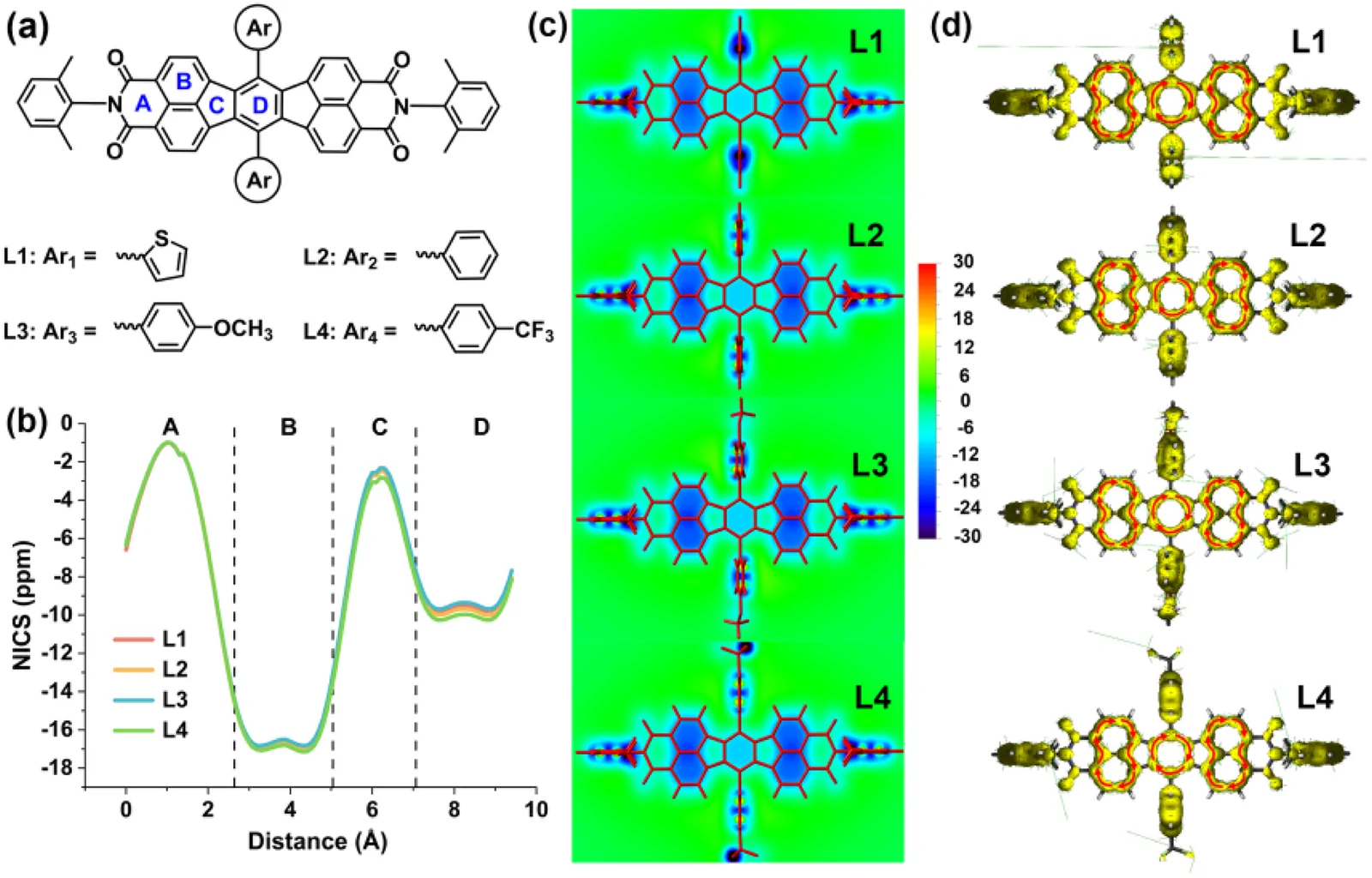
(a) Calculated molecular models of L1–L4. (b) Calculated NICS(1.7)-XY profiles and (c) 2D-NICS maps of L1–L4. (d) Calculated π-only AICD plots (isovalue: 0.035) of L1–L4. The orientation of the magnetic field is perpendicular to the paper plane, and the diamagnetic ring currents are indicated by red arrows.

Aromaticity is an important property of non-alternant fused-ring systems. To gain deeper insights, we studied the electronic properties by nucleus-independent chemical shift (NICS), 2D-NICS map and anisotropy of the induced current density (AICD) calculations based on the simplified model systems (also named L1–L4). As shown in Fig. 4b and S13, varying aryl substituents of L1–L4 show less pronounced effects on both NICS-XY profiles and NICS(1.7)_ZZ_ values. In all of the systems, we observe more negative NICS(1.7)_ZZ_ values for the naphthalene subunits (B ring, −16.6 to −16.9 ppm) and the central benzene subunits (D ring, −9.4 to −10.0 ppm) compared to the fused-pentagon rings (C ring, −2.6 to −3.1 ppm, Fig. S13). The results indicate that the fused-pentagon rings are anti-aromatic species. In particular, the significantly weakened aromaticity for the D ring is attributable to the fusion of two anti-aromatic pentagon rings in L1–L4. This local aromatic character is further supported by 2D-NICS analysis (Fig. 4c), which visually confirms the strong aromaticity for naphthalene subunits, the weakened aromaticity for central benzene rings and the non-aromatic and anti-aromatic characters for imide and fused-pentagon units, respectively. The AICD plots (Fig. 4d) further reveal that the clockwise ring currents are clearly observed on the benzene and naphthalene subunits of L1–L4. The antiaromatic pentagonal rings disrupt their global aromaticity, resulting in a local aromaticity character for these nonalternant π-systems. The results observed in AICD plots match well with the NICS-XY and 2D-NICS data. Note that the B rings maintain good aromaticity in the excited state, showing slightly reduced NICS(1.7)zz values compared to those of the ground state (Fig. S14 and S15). Unlike their ground state, the aryl-linked central D rings exhibit pronounced anti-aromaticity in the excited state (with NICS(1.7)zz values of 13.3 to 15.1 ppm) and also weaken the aromaticity of the neighboring pentagonal C rings. Consequently, the central rings exhibit local Hückel aromatic character, which gives rise to relatively high S_1_ levels of about 2.4 eV. Meanwhile, the excited-state stability of these π-systems is further supported by the strong aromatic nature of the adjacent naphthalene subunits and aryl substituents.

Considering that L1–L4 show a series of unique benefits, including good chemical stability, large nonalternant π-framework and strong intramolecular D–A interaction, we thus decided to examine their NLO properties and application potentials in organic laser protection fields. Firstly, the NLO properties of L1–L4 were investigated using the Z-scan technique, and the experimental details are provided in the SI. All the samples dissolved in *o*-dichlorobenzene were controlled with an identical linear transmittance (*T*_lin_) of 60% at 532 nm and then placed in a 1 mm quartz cuvette for open-aperture Z-scan measurement (Fig. S16). For comparison, the well-known NLO material, C_60_, was also applied. As shown in Fig. 5a–c, L1–L4 and C_60_ all exhibit typical NLO responses of RSA, which is evidenced by the valley-shaped transmittance profiles.^3,15^ The RSA response of the measured samples increases as the input laser energies (*E*_in_) increase from 5 to 15 µJ. It was found that regulating aryl substituents of L1–L4 shows pronounced effects on their RSA responses, which could be associated with the different ESA properties as revealed in the transient absorption spectra (for the details, see Fig. 8). The minimum normalized transmittance (*T*_min_) of the measured samples, which was determined from the normalized transmittance at the position of *Z* = 0, was found to gradually decrease as the *E*_in_ values increased from 5 to 15 µJ (Fig. 5a–c). Specifically, the *T*_min_ values of L1–L4 were determined to be 0.28, 0.25, 0.44, and 0.41 at an *E*_in_ of 5 µJ (Table S6), respectively, all of which are lower than that of C_60_ (*T*_min_ = 0.5). When the *E*_in_ value reaches 15 µJ, the *T*_min_ values of some materials are further decreased to 0.15 (L1), 0.15 (L2), and 0.17 (L4), much lower compared to C_60_ (*T*_min_ = 0.36, Table S6). Such low *T*_min_ values effectively demonstrate that the RSA responses of L1, L2, and L4 are more outstanding compared to that of C_60_. This improvement in RSA responses can be associated with their combined benefits of a large nonalternant π-framework, unique intramolecular D–A interaction, and efficient electron delocalization.

**Fig. 5:**
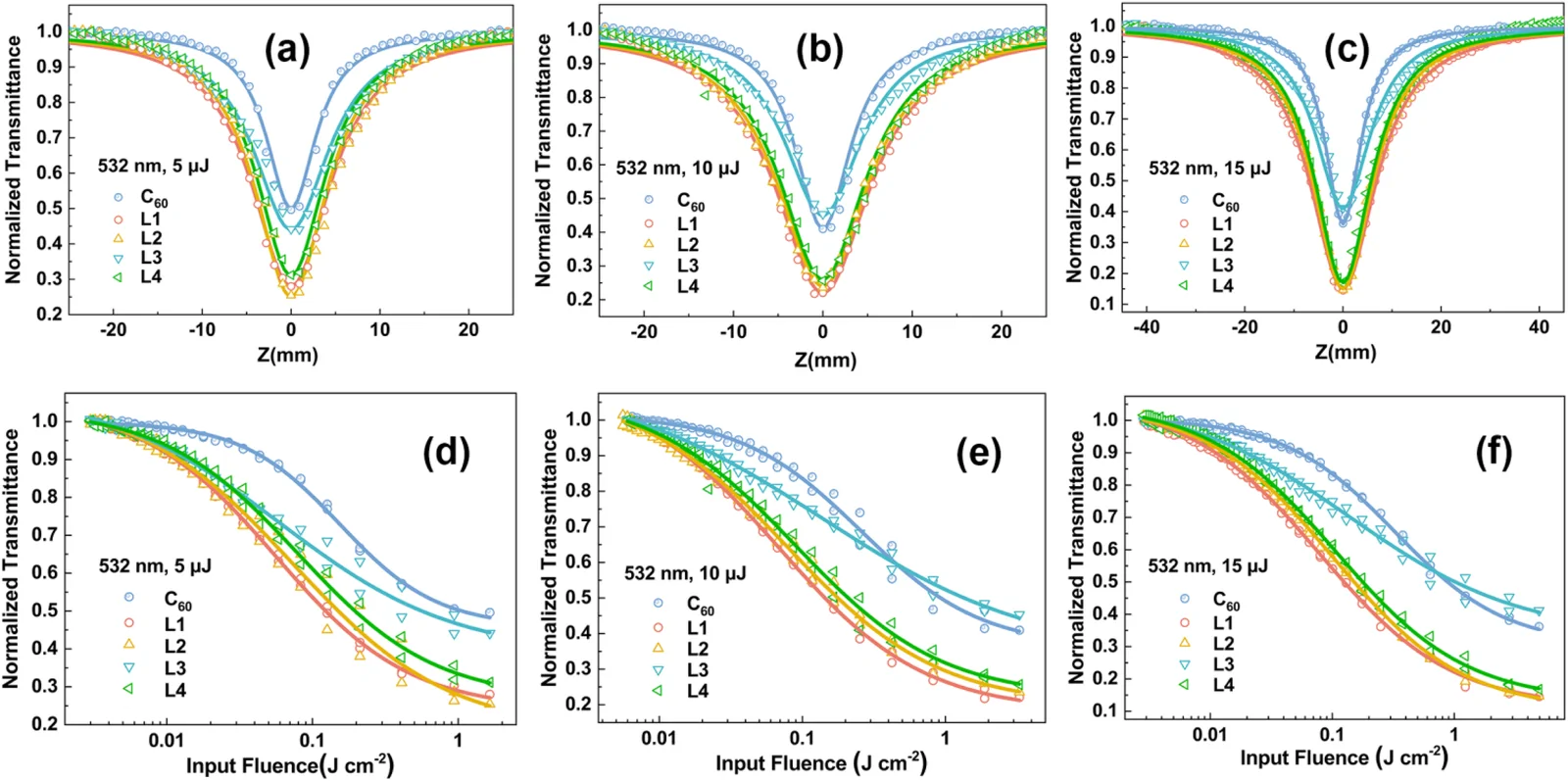
(a–c) The Z-scan traces of L1–L4 and C_60_ with the input laser energies of 5 µJ, 10 µJ, and 15 µJ. (d–f) The plots of normalized transmittance *versus* input fluence of L1–L4 and C_60_ at the incident laser energies of 5 µJ, 10 µJ, and 15 µJ.

**Fig. 6:**
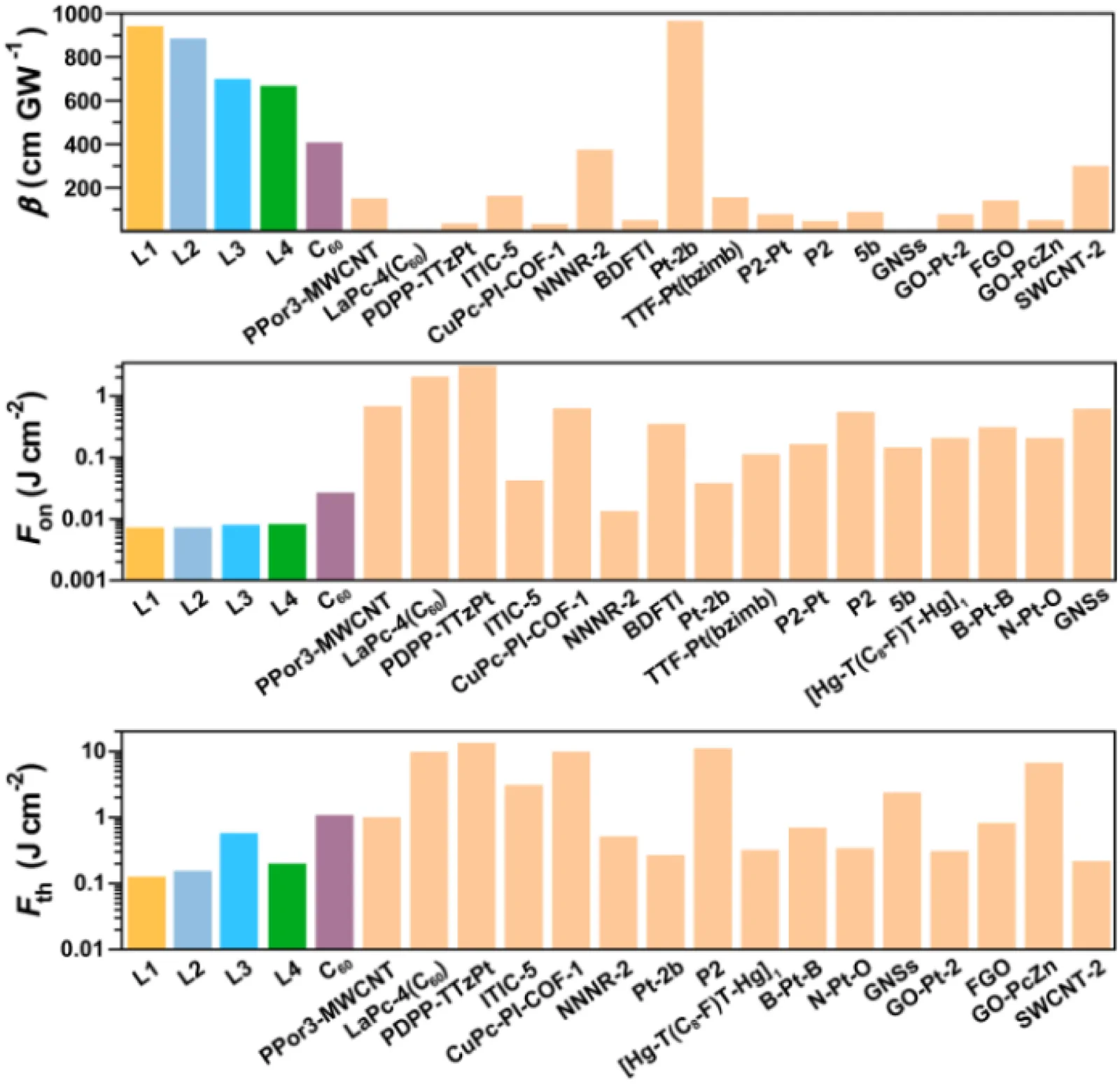
Comparison of the *β*, *F*_on_, and *F*_th_ values of L1–L4, C_60_, and previously reported materials.

To gain insight into their differences, the NLO performance of L1–L4 at a 5 µJ laser pulse was selected for detailed discussion. In comparison, the *T*_min_ values of L1 and L2 are lower than those of L3 and L4, whereas the nonlinear absorption coefficients (*β*_eff_) of L1 (941 cm GW^−1^) and L2 (885 cm GW^−1^) surpass those of L3 (698 cm GW^−1^) and L4 (668 cm GW^−1^, Table S6). In general, the lower *T*_min_ and larger *β*_eff_ values indicate much better NLO performance. The *β*_eff_ value of 941 cm GW^−1^ represents a high level, almost 2.3 times higher than that of C_60_ (407 cm GW^−1^, Table S6), significantly outperforming most of the previously reported NLO materials (Fig. 6 and Table S7), such as carbon-based materials,^58–63^ porphyrin derivatives,^62^ phthalocyanine derivatives,^20,58,60,63^ organic conjugated materials,^15,43,64,65^ and Pt(ii)-incorporated complexes.^22,61,66,67^ With the increase in *E*_in_ values, the corresponding *β*_eff_ values for each sample gradually decline owing to the saturation of RSA (Table S6). However, the *β*_eff_ values of L1–L4 still maintain a rather high level (321–557 cm GW^−1^) even at a high *E*_in_ value of 15 µJ, far higher than that of their counterpart C_60_ (225 cm GW^−1^, Table S6). In addition, the ratio of the effective excited-state absorption cross section and the ground-state absorption cross section (*σ*_ex_/*σ*_0_) values was further calculated to evaluate their RSA ability. Among all compounds, L3 displays the lowest *σ*_ex_/*σ*_0_ value of 1.83 at a 5 µJ laser pulse, whereas L1, L2, and L4 exhibit larger *σ*_ex_/*σ*_0_ values (*i.e.*, 2.34, 2.41, and 2.66, respectively) than C_60_ (1.84, Table S6). When applying 10 and 15 µJ laser pulses, the corresponding *σ*_ex_/*σ*_0_ values of L1–L4 and C_60_ were found to increase further (Table S6), while their variation tendency also follows the one observed at an *E*_in_ of 5 µJ. In theory, a larger *σ*_ex_/*σ*_0_ value is indicative of stronger excited-state absorption that facilitates the improvement of the RSA response.^15,64,65^

The plots of normalized transmittance *versus* input fluence were further extracted by theoretical fitting of the Z-scan traces in order to evaluate their application potential in laser OL fields. As depicted in Fig. 5d–f, the transmittance of L1–L4 shows no appreciable decrease before reaching the optical energy-limiting onset fluence (*F*_on_, defined as the input laser fluence at which the *T*_norm_ value falls to 0.95) at the applied incident laser energies of 5–15 µJ. However, when the intensity increased as the sample moved toward the laser focus, the *T*_norm_ value decreased and deviated from linearity, which is indicative of typical OL behavior. When applying a 5 µJ laser pulse, L1–L4 exhibit similar and small *F*_on_ values of 7–8 mJ cm^−2^, far lower than those of C_60_ (26 mJ cm^−2^, Table S6) and many classical OL materials reported in the literature (Fig. 6 and Table S7), such as carbon-based materials (*e.g.*, PPor3-MWCNT, 0.66 J cm^−2^;^62^LaPc-4(C_60_), *ca.* 2.0 J cm^−2^ (ref. 63) and GNSs, 0.06 J cm^−2^ (ref. 8)), organic conjugated materials (ITIC-5, *ca.* 0.041 J cm^−2^;^64^NNNR-2, 0.013 J cm^−2^;^15^BDFTI, 0.34 J cm^−2^;^43^P2, 0.53 J cm^−2^ (ref. 16) and 5b, 0.14 J cm^−2^ (ref. 65)), and metal-containing complexes (PDPP-TTzPt, *ca.* 3.0 J cm^−2^;^67^CuPc-PI-COF-1, 0.61 J cm^−2^;^20^Pt-2b, 0.037 J cm^−2^;^68^[Hg-T(C_8_-F)T-Hg]_1_, 0.02 J cm^−2^;^69^B-Pt-B, *ca.* 0.03 J cm^−2^ (ref. 21) and N-Pt-O, *ca.* 0.02 J cm^−2^ (ref. 21)). Moreover, their *F*_on_ values measured at both 10 and 15 µJ are also smaller compared to C_60_ (Table S6). A much smaller *F*_on_ value indicates that the OL properties of L1–L4 are superior to those of C_60_.

Another important parameter for evaluating the merits of OL materials is the OL threshold (*F*_th_), which is defined as the input laser fluence at a *T*_norm_ value of 0.5. In the present work, the *F*_th_ values of L1–L4 are found to be rather small (ranging from 0.123 to 0.195 J cm^−2^, Table S6), all of which are remarkably smaller than that of C_60_ (1.049 J cm^−2^) measured at an identical *E*_in_ of 5 µJ. Among them, L1 manifests the lowest *F*_th_ value of 0.123 J cm^−2^, whose characteristics favourably approach the requirement of a practical laser-protecting device. It should be highlighted that the *F*_th_ values of L1–L4 are far lower than those of many classical OL materials reported in the literature (Fig. 6 and Table S7), such as carbon-based materials (*e.g.*, PPor3-MWCNT, 0.98 J cm^−2^;^62^LaPc-4(C_60_), 9.5 J cm^−2^;^63^GNSs, 2.3 J cm^−2^ (ref. 8) and GO-Pt-2, 0.3 J cm^−2^ (ref. 61)), organic conjugated materials (ITIC-5, *ca.* 3.0 J cm^−2^;^64^NNNR-2, 0.5 J cm^−2^ (ref. 15) and P2, 10.0 J cm^−2^ (ref. 16)), and metal-containing complexes (PDPP-TTzPt, *ca.* 13 J cm^−2^;^67^CuPc-PI-COF-1, 9.55 J cm^−2^;^20^Pt-2b, 9.55 J cm^−2^;^68^[Hg-T(C_8_-F)T-Hg]_1_, 0.31 J cm^−2^ (ref. 69) and GO-Pt-2, 0.30 J cm^−2^ (ref. 61)). Considering that the OL performance depends strongly on the different measurement conditions, the current comparison between L1–L4 and the selected examples is not fully rigorous. However, this comparison indirectly reflects the effectiveness of our molecular design strategy towards constructing high-performance OL materials. As we know, C_60_ is a well-known OL material that has been widely employed as a standard material to evaluate the OL performance of new materials.^14,15,66^ In the present case, the combined benefits of the lower *T*_min_ values (except for L3) coupled with the smaller *F*_on_ and *F*_th_ values than the corresponding values of C_60_ demonstrate that the OL performance of our material system is superior to that of C_60._ The comparison with C_60_ under the same experimental conditions thus supports the reliability of the OL results. Taken together, aryl-substituted acenaphtho[1,2-*k*]fluoranthene diimide derivatives are proven to be a family of excellent OL materials, holding promising potential in laser protection applications.

To further demonstrate their application potentials in organic laser protection fields, the OL properties of L1–L4 toward a 532 nm pulsed laser were further studied in *o*-dichlorobenzene with an identical linear transmittance of 60% at 532 nm. As depicted in Fig. 7a, the output fluences of L1–L4 evidently deviate from linearity along with the increase of input fluence, demonstrating obvious OL abilities for these materials. A decreasing tendency of L1 > L2 > L4 > L3 is also observed, consistent with the NLO properties observed in Z-scan experiments. As shown in Fig. 7b, the OL behaviors of L1, L2 and L4 are almost similar to each other, and their *F*_th_ values are determined to be 0.32, 0.37 and 0.41 J cm^−2^, respectively. Among them, L3 bearing two 4-methoxyphenyl substituents shows the worst OL ability with the largest *F*_th_ of above 1.40 J cm^−2^. To our delight, the *F*_th_ values of L1, L2 and L4 are still lower than that of C_60_ (0.82 J cm^−2^), further demonstrating their superior OL performance and more promising potential in laser protection applications.

**Fig. 7:**
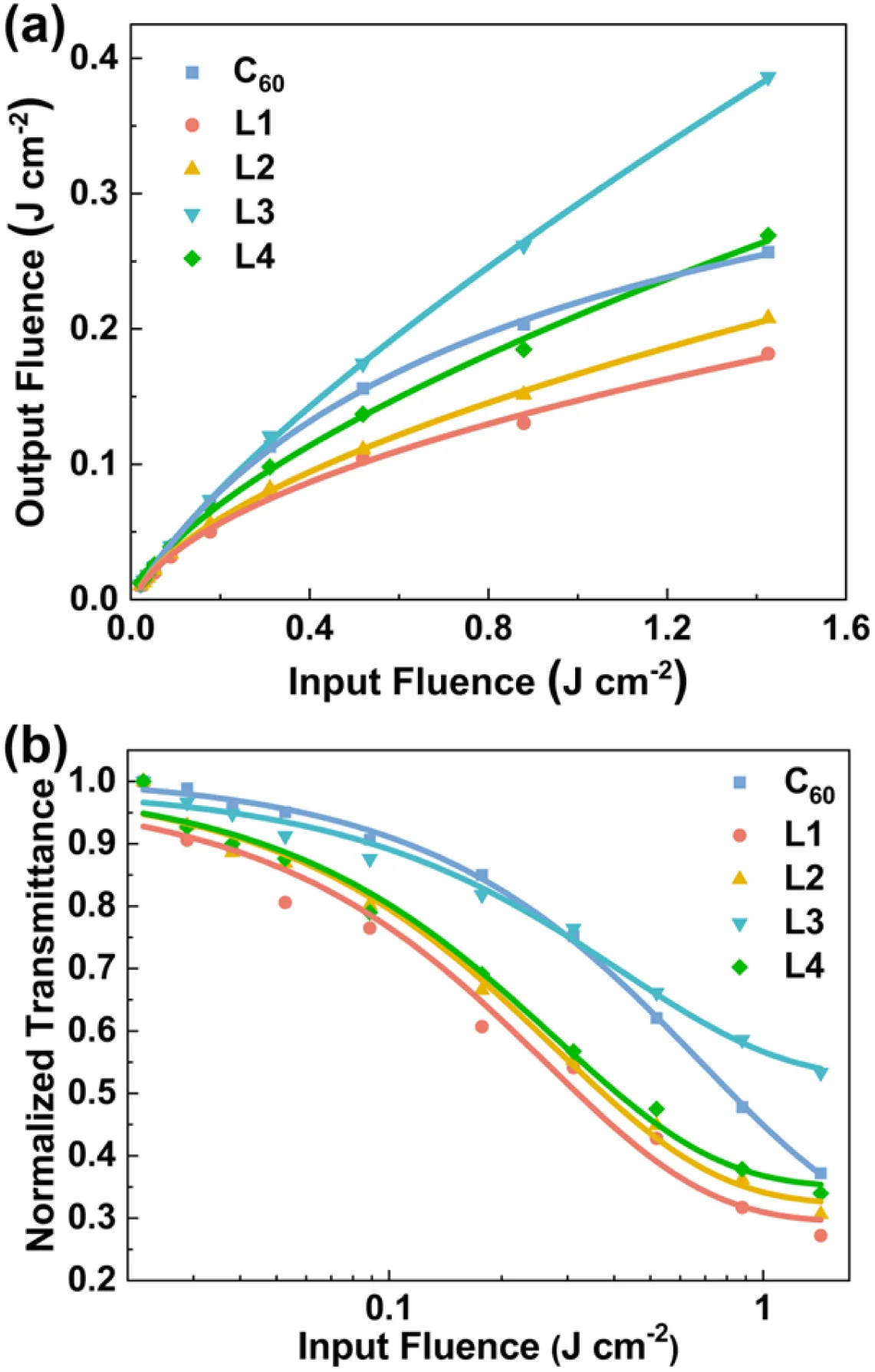
The optical-limiting properties of L1–L4 and C_60_ upon treatment with a 532 nm pulsed laser. (a) Typical profiles of output fluence *vs.* input fluence and (b) normalized transmittance *vs.* input fluence.

**Fig. 8:**
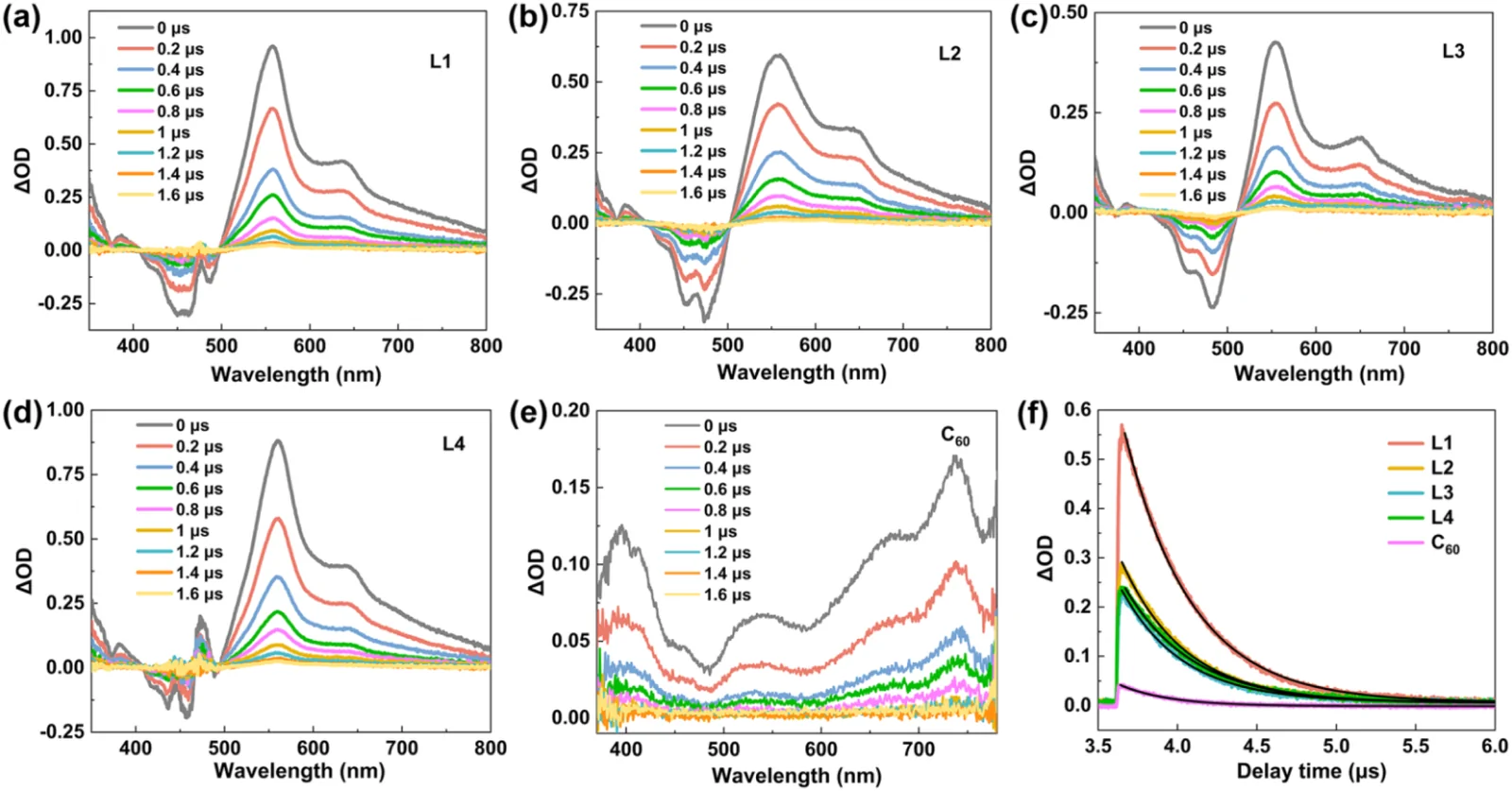
(a–e) Nanosecond transient absorption spectra of L1–L4 and C_60_. (f) Dynamics traces of the selected wavelength at 532 nm for L1–L4 and C_60_.

To understand the performance difference and OL response mechanism of our samples in depth, the nanosecond time-resolved transient absorption (TA) spectra of L1–L4 were further examined *via* pump-probe experiments. As shown in Fig. 8a–d, L1–L4 all exhibit distinct saturable absorption signals before around 500 nm, featuring the strongest peaks centered at around 457, 473, 483, and 457 nm, respectively. These saturable absorption signals correlate precisely with the absorption maxima observed in the corresponding UV-vis absorption spectra (Fig. 3a). After around 500 nm, L1–L4 all show strong and broadband ESA, along with the largest ESA intensity appearing around 550 nm (Fig. 8a–d). Among them, L1 achieves the largest absorption intensity, significantly larger than that of L3. In addition, the decay kinetics extracted from TA spectra (Fig. 8f) at 532 nm reveals the excited-state lifetimes of 421 ns (L1), 400 ns (L2), 386 ns (L3), and 427 ns (L4). The strongest ESA and relatively long excited-state lifetimes for L1 could be responsible for explaining why L1 delivers more efficient RSA response and better OL performance than L2–L4, particularly compared to L3. Among them, compound L3 displays the strongest ground-state absorption at 532 nm (Fig. 3a), which is due to its strong ICT interaction induced by the electron-rich 4-methoxyphenyl substituents. Generally, the strong ground-state absorption at 532 nm is unfavourable for accessing strong ESA at the same wavelength. As a result, L3 is proven to be the weakest one in terms of ESA at 532 nm (Fig. 8). These results further indicate that the weakest ESA and the shortest excited-state lifetime for L3 result in a relatively weakened RSA response and lower OL performance (see Fig. 7) compared to the other three compounds. However, L3 still demonstrates superior RSA response relative to C_60_. The results observed here thus indicate that regulating aryl substituents of this material system is an effective approach towards effective modulation of their ESA, NLO response and OL performance. For example, materials incorporating electron-rich aryl substituents could access stronger ICT interaction and higher intensity of ground-state absorption at 532 nm, while featuring relatively weak ESA that could be responsible for relatively weakened OL performance. Furthermore, the combined benefits of a large nonalternant π-framework and unique intramolecular D–A interaction can indeed facilitate π-electron delocalization and ESA of these materials, thereby achieving more outstanding NLO responses compared to C_60_.

## Conclusions

We have developed a series of diimide-functionalized cyclopenta-fused polycyclic aromatic hydrocarbons, namely acenaphtho[1,2-*k*]fluoranthene diimide derivatives (L1–L4), for high-performance optical-limiting (OL) applications. These aryl-substituted diimides featuring a 3D-like single-crystal structure exhibit excellent solubility in most common solvents. Incorporating electron-poor fused-pentagon and imide subunits into these large nonalternant π-frameworks endows them with good chemical stability, efficient π-electron delocalization, and unique intramolecular donor–acceptor interaction, all of which are extremely important for improving OL response based on the RSA mechanism. Employing open-aperture Z-scan experiments, these diimides are demonstrated to deliver outstanding OL performance featuring extremely low *F*_th_ and *F*_on_ values, superior to those of many reported OL materials (see Fig. 6). Among them, the thiophene-bearing L1 showcases the lowest *F*_on_ of 7 mJ cm^−2^ and *F*_th_ of 0.123 J cm^−2^ and the largest *β* of 941 cm GW^−1^, significantly outperforming those of benchmark C_60_ upon treatment with 532 nm nanosecond laser pulses. The nanosecond transient absorption spectra reveal that, for such material systems, high OL performance originates from the RSA mechanism and benefits from their strong ESA and long excited-state lifetimes. Our findings offer encouraging progress towards the design and synthesis of materials of this type with outstanding OL performance, highlighting nonalternant fused-ring diimides as a new material system for future nonlinear optics.

## Author contributions

J. Lan: conceptualization, investigation, and writing – original draft. Z. Ma: investigation and methodology. W. Liu: investigation and data curation. S. Zeng: formal analysis and data curation. G. Bin: data curation and theoretical calculation. L. Ren: investigation. L. Zheng: supervision and project administration. C. Sun: supervision. J. Sun: supervision, project administration, and writing – review and editing. H. Chen: conceptualization, supervision, writing – review and editing, project administration, and funding acquisition.

## Conflicts of interest

The authors declare no conflict of interest.

## Supplementary Material

SC-017-D6SC05845K-s001

SC-017-D6SC05845K-s002

## Data Availability

The data that support the findings of this study have been included as part of the supplementary information (SI). Supplementary information: experimental conditions, material synthesis, photophysical and electrochemical data, crystal data, theoretical calculations, Z-scan experimental details, and NMR characterization. See DOI: https://doi.org/10.1039/d6sc05845k. CCDC 2511982 (L2), 2511984 (L3), 2511985 (L4) and 2511986 (L1) contain the supplementary crystallographic data for this paper.^57*a*–*d*^
