# Supplementary material for: Nonalternant acenaphtho[1,2-k]fluoranthene diimide derivatives with outstanding optical-limiting performance
Source: Chem Sci. 2026 Sep 17;17(42):20655–64. doi: 10.1039/d6sc05845k (PMC13592094; doi:10.1039/d6sc05845k)
Supplement: Supplementary file 1 [file SC-017-D6SC05845K-s001.pdf]

Supplementary Information (SI) for Chemical Science.
This journal is © The Royal Society of Chemistry 2026

Supplementary Information

Nonalternant Acenaphtho[1,2-*k*]fluoranthene Diimide Derivatives with Outstanding Optical-Limiting Performance

Jiaxin Lan[a,#], Zhipeng Ma[a,b,#], Wentao Liu[a], Shuhao Zeng[a], Guangxiong Bin[a], Longbin Ren[c], Liping Zheng[a,*], Chenghua Sun[b], Jibin Sun[b,*], Huajie Chen[a,*]

[a]. J. Lan, Z. Ma, W. Liu, S. Zeng, Dr. L. Zheng, Prof. H. Chen
Key Laboratory of Environmentally Friendly Chemistry and Application of Ministry of Education, College of Chemistry, Xiangtan University, Xiangtan 411105, P. R. China.
E-mail: chenhjoe@xtu.edu.cn; zhenglp@xtu.edu.cn

[b]. Prof. C. Sun, Prof. J. Sun
Key Laboratory of Photochemical Conversion and Optoelectronic Materials, Technical Institute of Physics and Chemistry, Chinese Academy of Sciences, Beijing 100190, P. R. China.
E-mail: sunjibin@mail.ipc.ac.cn

[c]. Dr. L. Ren
Beijing National Laboratory for Molecular Sciences, Organic Solids Laboratory, Institute of Chemistry, Chinese Academy of Sciences, Beijing 100190, P. R. China

Contents

1. Measurement S2

2. Synthesis of Materials S2

3. FT-IR Spectrometer S7

4. Single Crystal X-ray Crystallographic Data S8

5. Photophysical and Electrochemical Properties S14

6. Theoretical Calculations S15

7. Z-Scan Experiments S19

8. ^{1}H and ^{13}C NMR Spectra of the Novel Compounds S23

9. Computational Atomic Coordinate S32

10. References S43

1. Measurement

The ^{1}H and ^{13}C NMR spectra in deuterated solvents (e.g., $CDCl_3$) were collected on a Bruker AVANCE 400 spectrometer with *tetra*-methylsilane (TMS) as the internal reference. High-resolution MALDI-TOF mass spectra were recorded on a Bruker autoflex III smartbeam MALDI-TOF Bruker instrument. High-resolution electrospray ionization (ESI) mass spectra were collected on an Orbitrap Fusion Lumos instrument. The absorption spectra of each sample in chloroform were measured on an Agilent Cary 60 UV-Vis spectrophotometer. The photoluminescence (PL) spectra were recorded in chloroform by using a Hitachi F-4600 spectrophotometer. To determine the redox potentials of each sample, cyclic voltammetry (CV) was performed on an electrochemical workstation (CHI660E, Shanghai CHENHUA) with the scan rate of 100 mV s^{-1}. The three-electrode electrochemical cell contains a glassy carbon electrode, a Pt wire, and an Ag/AgCl (KCl, Sat'd) electrode, which, respectively were used as the working electrode, counter electrode, and reference electrode. An anhydrous and N_2-saturated dichloromethane solution of *tetra*-*n*-butylammonium hexafluorophosphate ($TBAPF_6$, 0.1 M) was used as the supporting electrolyte. The redox potentials of each sample were calibrated with the ferrocene/ferrocenium (Fc/Fc^+) redox couple, whose the onset value of oxidation potential was determined as 0.38 V vs Ag/AgCl. The LUMO energies of these samples were thus calculated from the empirical equation of $E_{LUMO} = -(E_{red} + 4.42)$ eV[1]. The Z-scan experimental system was set up with a Q-switched Nd:YAG laser, which was applied to generate 8 ns pulses with a repetition frequency of 10 Hz at the wavelength of 532 nm. Samples dissolved in *o*-dichlorobenzene were prepared with the identical linear transmittance of 60% at the wavelength of 532 nm and were placed in 1 mm quartz cuvette for open-aperture Z-scan experiment. A nanosecond time-resolved transient absorption (TA) was conducted by using pump-probe experiments on a LP980KS Edinburgh Instruments.

2. Synthesis of Materials

Materials. All the chemical reagents and organic solvents were purchased from Chemical Greatwall. The key starting materials, compounds **1** and **2**, were synthesized using a reported method[1]. Four key ketone-bearing intermediate compounds, including 1,3-di(thiophen-2-yl)propan-2-one (**5a**)[2], 1,3-diphenylpropan-2-one (**5b**)[3], 1,3-bis(4- methoxyphenyl)-propan-2-one (**5c**)[4], and 1,3-bis(4-(trifluoromethyl)phenyl)propan-2-one (**5d**)[5], were prepared following the literature procedures.

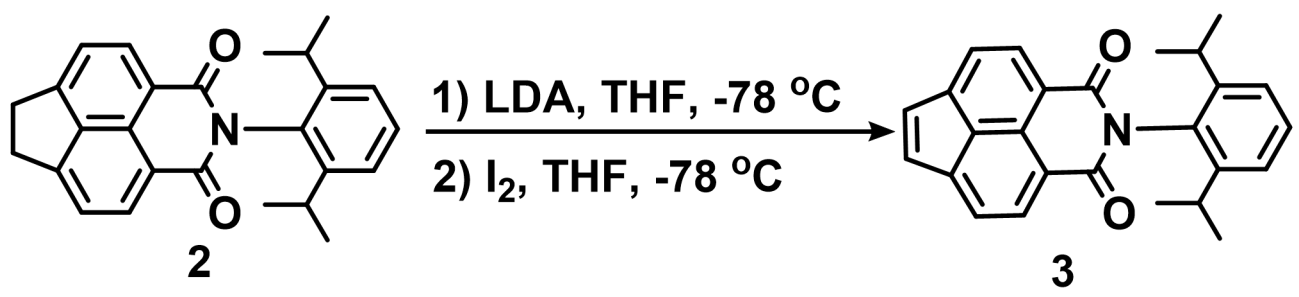

Synthesis of compound 3. Under nitrogen atmosphere, a mixture of compound **2** (0.80 g, 2.09 mmol) and anhydrous THF (40 mL) was stirred at –78 °C for 20 min, followed by the addition of lithium diisopropylamide (3.13 mL, 6.26 mmol) dropwise, and then kept stirring for another 1 h. The reaction mixture was then warmed to –40 °C and then kept stirring for another 1 h. After that, an anhydrous THF solution containing iodine (1.59 g, 6.26 mmol) was slowly dropped into the reaction mixture at –78 °C, and then stirred at room temperature overnight. After being quenched with sodium bisulfite aqueous solution (200 mL, 4.3 M), the organic phase was separated by dichloromethane extraction and then dried with anhydrous $MgSO_4$. After removal of the solvent, the residue was further purified by column chromatography (PE: DCM = 2:1) to afford red solid (315 mg, 40%). ^{1}H NMR (400 MHz, $CDCl_3$) δ (ppm): 8.42 (d, J = 8.0 Hz, 2H), 7.79 (d, J = 8.0 Hz, 2H), 7.46 (t, J = 8.0 , 4.0 Hz, 1H), 7.31 (d, J = 8.0 Hz, 2H), 7.14 (s, 2H), 2.82 – 2.75 (m, 2H), 1.15 (d, J = 8.0 Hz, 12H). ^{13}C NMR (100 MHz, $CDCl_3$) δ (ppm): 164.13, 146.07, 144.86, 133.20, 132.42, 131.13, 130.78, 129.58, 127.80, 125.31, 124.04, 122.88, 29.23, 24.14. HRMS: m/z $[M+H]^+$ calcd for ($C_{26}H_{24}NO_2$) 382.1801; found 382.1801 (error = -0.1 ppm).

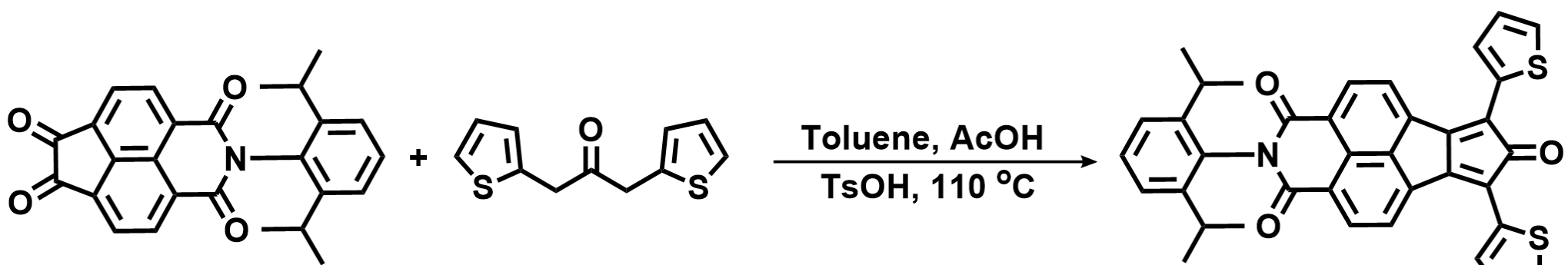

Synthesis of compound 6a. Under nitrogen atmosphere, a mixture of compound **4** (100 mg, 0.24 mmol), compound **5a** (54.1 mg, 0.24 mmol), toluene (4 mL), acetic acid (1 mL) and 4-methylbenzenesulfonic acid (10 mg, 0.06 mmol) was stirred at 110 °C overnight. The organic phase was obtained by dichloromethane extraction and then dried with $MgSO_4$. After removal of the solvent, the crude product was purified by column chromatography (PE: DCM = 2:1) to afford black solid (65 mg, 45%). ^{1}H NMR (400 MHz, $CDCl_3$) δ (ppm): 8.73 (d, J = 8.0 Hz, 2H), 8.66 (d, J = 8.0 Hz, 2H), 8.09 (d, J = 4.0 Hz, 2H), 7.65 (d, J = 8.0 Hz, 2H), 7.50 (t, J = 8.0 Hz, 1H), 7.36 (d, J = 8.0 Hz, 2H), 7.30 (t, J = 4.0 Hz, 2H), 2.81 – 2.74 (m, 2H), 1.19 (d, J = 8.0 Hz, 12H). The ^{13}C NMR of compound 4 is hardly accessible due to its poor solubility at room temperature. HRMS: m/z $[M+H]^+$ calcd for ($C_{37}H_{28}NO_3S_2$) 598.1504; found 598.1505 (error = -0.1 ppm).

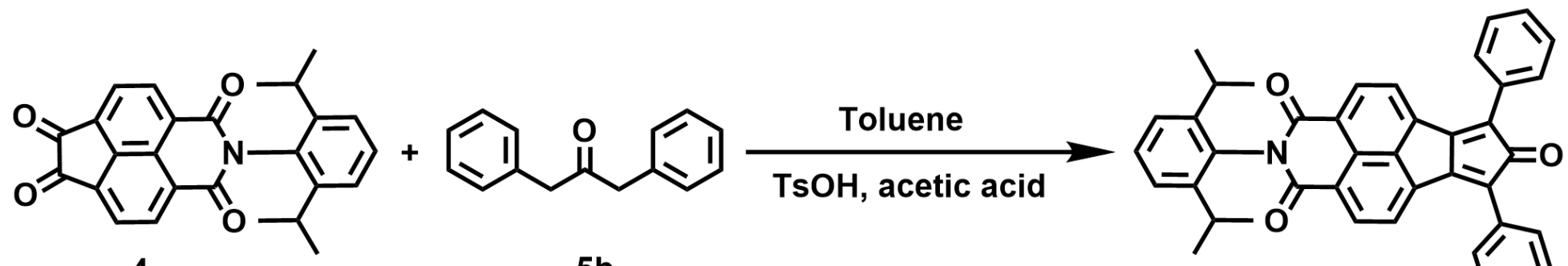

4 5b 6b

Synthesis of compound 6b. Under nitrogen atmosphere, a mixture of compound **4** (100 mg, 0.24 mmol), compound **5b** (51.1 mg, 0.24 mmol), toluene (4 mL), acetic acid (1 mL) and 4-methylbenzenesulfonic acid (10 mg, 0.06 mmol) was stirred at 110 °C overnight. The organic phase was obtained by dichloromethane extraction and then dried with $MgSO_4$. After removal of the solvent, the crude product was purified by column chromatography (PE: DCM = 2:1) to afford black solid (57 mg, 40%). ^{1}H NMR (400 MHz, $CDCl_3$) δ (ppm): 8.68 (d, J = 8.0 Hz, 2H), 8.33 (d, J = 8.0 Hz, 2H), 7.90 (d, J = 8.0 Hz, 4H), 7.61 (t, J = 8.0 Hz, 4H), 7.53 (d, J = 8.0 Hz, 2H), 7.49 (d, J = 4.0 Hz, 1H), 7.35 (d, J = 8.0 Hz, 2H), 2.78 – 2.71 (m, 2H), 1.17 (d, J = 8.0 Hz, 12H). ^{13}C NMR (100 MHz, $CDCl_3$) δ (ppm): 200.59, 163.66, 151.82, 145.76, 143.23, 136.72, 133.46, 131.06, 130.58, 129.77, 129.71, 129.35, 129.14, 127.93, 126.22, 124.24, 122.53, 121.51, 29.27, 24.16. HRMS: m/z $[M+H]^+$ calcd for ($C_{41}H_{32}NO_3$) 586.2375; found 586.2376 (error = 0.2 ppm).

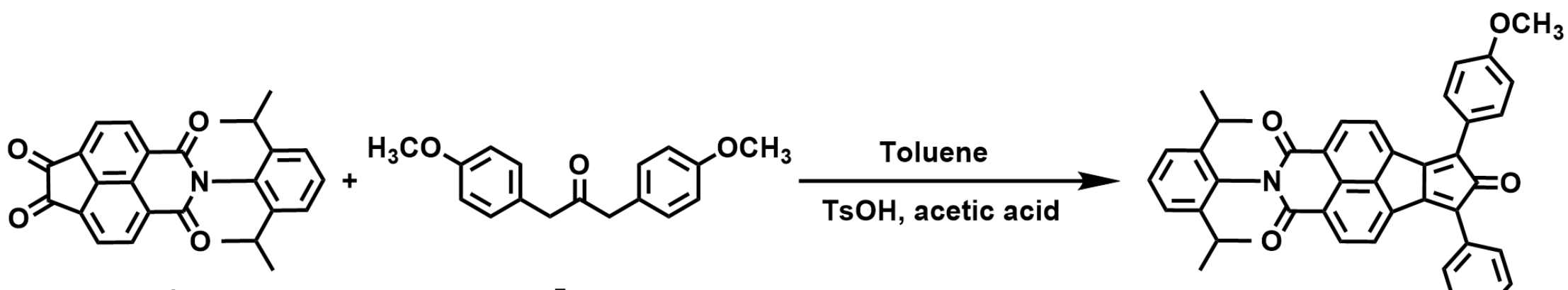

4 5c 6c

Synthesis of compound 6c. Under nitrogen atmosphere, a mixture of compound **4** (100 mg, 0.24 mmol), compound **5c** (65.7 mg, 0.24 mmol), toluene (4 mL), acetic acid (1 mL) and 4-methylbenzenesulfonic acid (10 mg, 0.06 mmol) was stirred at 110 °C overnight. The organic phase was obtained by dichloromethane extraction and then dried with $MgSO_4$. After removal of the solvent, the crude product was purified by column chromatography (PE: DCM = 2:1) to afford black solid (67 mg, 43%). ^{1}H NMR (400 MHz, $CDCl_3$) δ (ppm): 8.66 (d, J = 8.0 Hz, 2H), 8.29 (d, J = 8.0 Hz, 2H), 7.88 (d, J = 8.0 Hz, 4H), 7.49 (t, J = 8.0 Hz, 1H), 7.35 (d, J = 8.0 Hz, 2H), 7.12 (d, J = 8.0 Hz, 4H), 3.93 (s, 6H), 2.79 – 2.72 (m, 2H), 1.17 (d, J = 8.0 Hz, 12H). ^{13}C NMR (100 MHz, $CDCl_3$) δ (ppm): 201.47, 163.79, 160.86, 150.09, 145.80, 143.07, 137.11, 133.43, 131.18, 130.90, 129.66, 128.03, 125.99, 124.22, 123.18, 121.98, 120.91, 114.69, 55.62, 29.26, 24.16. HRMS: m/z $[M+H]^+$ calcd for ($C_{43}H_{36}NO_5$) 646.2586; found 646.2588 (error = -0.3 ppm).

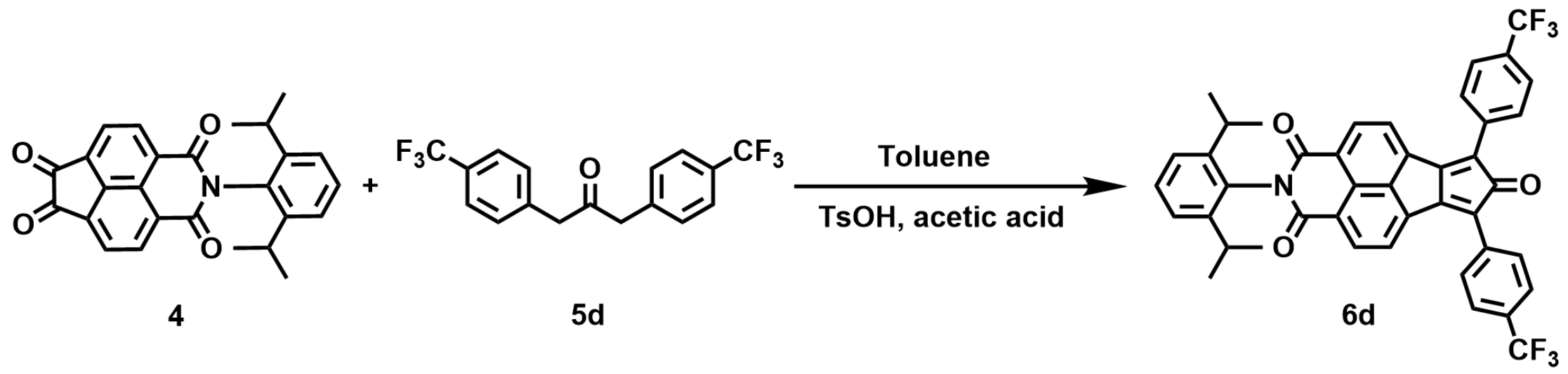

Synthesis of compound 6d. Under nitrogen atmosphere, a mixture of compound **4** (100 mg, 0.24 mmol), compound **5d** (84.2 mg, 0.24 mmol), toluene (4 mL), acetic acid (1 mL) and 4-methylbenzenesulfonic acid (10 mg, 0.06 mmol) was stirred at 110 °C overnight. The organic phase was obtained by dichloromethane extraction and then dried with $MgSO_4$. After removal of the solvent, the crude product was purified by column chromatography (PE: DCM = 2:1) to afford black solid (82.2 mg, 47%). ^{1}H NMR (400 MHz, $CDCl_3$) δ (ppm): 8.72 (d, J = 8.0 Hz, 2H), 8.31 (d, J = 8.0 Hz, 2H), 8.01 (d, J = 8.0 Hz, 4H), 7.87 (d, J = 8.0 Hz, 4H), 7.51 (t, J = 8.0 Hz, 1H), 7.36 (d, J = 8.0 Hz, 2H), 2.77 – 2.70 (m, 2H), 1.17 (d, J = 8.0 Hz, 12H). ^{13}C NMR (100 MHz, $CDCl_3$) δ (ppm): 199.41, 163.42, 153.46, 145.71, 143.45, 135.87, 133.83, 133.56, 130.82, 129.85, 129.61, 127.91, 126.18, 126.15, 124.64, 124.31, 123.31, 122.08, 29.85, 29.31, 24.16. HRMS: m/z $[M+H]^+$ calcd for ($C_{43}H_{30}F_6NO_3$) 722.2125; found 722.2124 (error = 0.2 ppm).

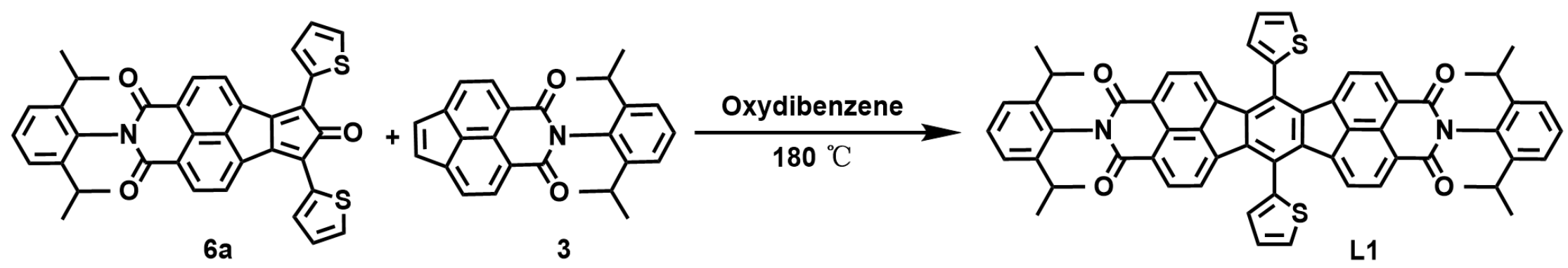

Synthesis of L1. Under nitrogen atmosphere, a mixture of compound **6a** (150 mg, 0.24 mmol), compound **3** (105 mg, 0.28 mmol), oxydibenzene (2 mL) was stirred at 180 °C for 12 h. After cooling down to the room temperature, the organic phase was obtained by dichloromethane extraction and then dried with $MgSO_4$. After removal of the solvent, the mixture was purified by column chromatography (PE: DCM = 2:1) to give red crude product. The crude product was settled with acetone to afford a bright red solid product (155 mg, 65%). ^{1}H NMR (400 MHz, $CDCl_3$) δ (ppm): 8.39 (d, J = 8.0 Hz, 4H), 7.81 (d, J = 8.0 Hz, 2H), 7.50 (t, J = 4.0 Hz, 2H), 7.45 (d, J = 8.0 Hz, 2H), 7.41 (d, J = 4.0 Hz, 2H), 7.31 (d, J = 8.0 Hz, 4H), 7.04 (d, J = 8.0 Hz, 4H), 2.76 – 2.72 (m, 4H), 1.13 (d, J = 8.0 Hz, 24H). ^{13}C NMR (100 MHz, $CDCl_3$) δ (ppm): 163.85, 145.92, 141.15, 140.95, 136.72, 132.91, 132.33, 131.04, 130.21, 129.62, 128.83, 128.08, 127.35, 125.66, 124.77, 124.09, 122.70, 29.26, 24.10. HRMS: m/z $[M+H]^+$ calcd for ($C_{62}H_{49}N_2O_4S_2$) 949.3127; found 949.3128 (error = -0.1 ppm).

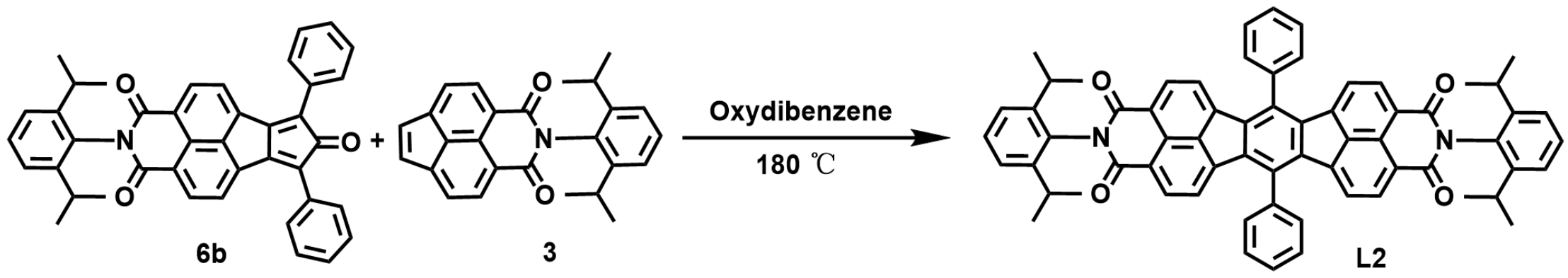

Synthesis of L2. Under nitrogen atmosphere, a mixture of compound **6b** (121.1 mg, 0.21 mmol), compound **3** (94.7 mg, 0.25 mmol), oxydibenzene (3 mL) was stirred at 180 °C for 12 h. After cooling down to the room temperature, the organic phase was obtained by dichloromethane extraction and then dried with $MgSO_4$. After removal of the solvent, the mixture was purified by column chromatography (PE: DCM = 1:1) to give red crude product. The crude product was settled with acetone to afford a bright red solid product (145 mg, 76%). ^{1}H NMR (400 MHz, $CDCl_3$) δ (ppm): 8.30 (d, J = 8.0 Hz, 4H), 7.79 (t, J = 4.0 Hz, 2H), 7.78 (d, J = 4.0 Hz, 4H), 7.68 (t, J = 4.0 Hz, 4H), 7.45 (t, J = 8.0 Hz, 2H), 7.29 (d, J = 8.0 Hz, 4H), 6.86 (d, J = 8.0 Hz, 4H), 2.76 – 2.69 (m, 4H), 1.12 (d, J = 4.0 Hz, 24H). ^{13}C NMR (100 MHz, $CDCl_3$) δ (ppm): 163.91, 145.91, 141.66, 139.45, 137.27, 137.16, 132.79, 132.42, 131.06, 130.27, 129.62, 129.58, 128.56, 125.64, 124.54, 124.06, 122.31, 29.23, 24.07. HRMS: m/z $[M+H]^+$ calcd for ($C_{66}H_{53}N_2O_4$) 937.3997; found 937.3999 (error = 0.3 ppm).

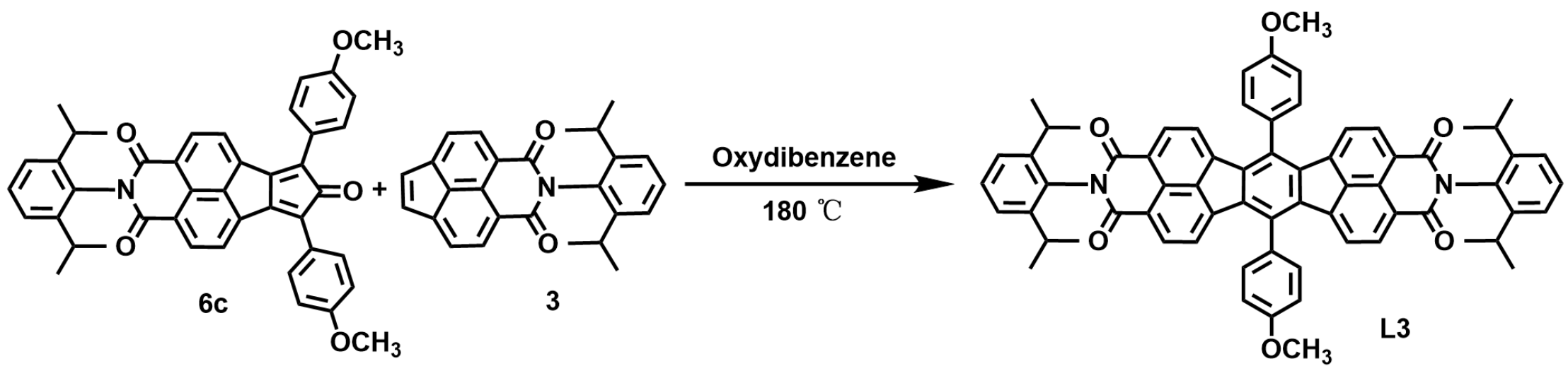

Synthesis of L3. Under nitrogen atmosphere, a mixture of compound **6c** (164 mg, 0.25 mmol), compound **3** (107 mg, 0.28 mmol), oxydibenzene (3 mL) was stirred at 180 °C for 12 h. After cooling down to the room temperature, the organic phase was obtained by dichloromethane extraction and then dried with $MgSO_4$. After removal of the solvent, the mixture was purified by column chromatography (PE: DCM = 2:1) to give red crude product. The crude product was settled with acetone to afford a bright red solid product (205 mg, 81%). ^{1}H NMR (400 MHz, $CDCl_3$) δ (ppm): 8.32 (d, J = 8.0 Hz, 4H), 7.58 (d, J = 8.0 Hz, 4H), 7.45 (t, J = 8.0 Hz, 2H), 7.31 (d, J = 4.0 Hz, 4H), 7.29 (d, J = 4.0 Hz, 4H), 6.96 (d, J = 8.0 Hz, 4H), 4.06 (s, 6H), 2.77 – 2.70 (m, 4H), 1.12 (d, J = 8.0 Hz, 24H). ^{13}C NMR (100 MHz, $CDCl_3$) δ (ppm): 163.94, 160.46, 145.89, 141.89, 139.88, 137.07, 132.80, 132.39, 131.08, 129.80, 129.57, 129.12, 125.64, 124.53, 124.05, 122.23, 115.55, 55.68, 29.22, 24.08. HRMS: m/z $[M+H]^+$ calcd for ($C_{68}H_{57}N_2O_6$) 997.4208; found 997.4211 (error = 0.2 ppm).

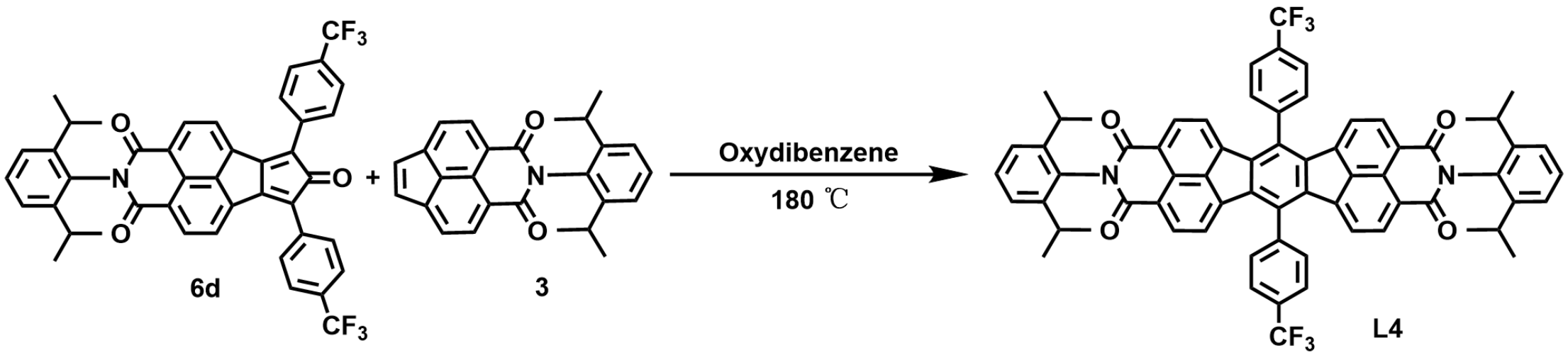

Synthesis of L4. Under nitrogen atmosphere, a mixture of compound **6d** (130 mg, 0.18 mmol), compound **3** (76 mg, 0.20 mmol), oxydibenzene (5 mL) was stirred at 180 °C for 12 h. After cooling down to the room temperature, the organic phase was obtained by dichloromethane extraction and then dried with $MgSO_4$. After removal of the solvent, the mixture was purified by column chromatography (PE: DCM = 1:1) to give red crude product. The crude product was settled with acetone to afford a bright red solid product (100 mg, 52%). ^{1}H NMR (400 MHz, $CDCl_3$) δ (ppm): 8.34 (d, J = 8.0 Hz, 4H), 8.10 (d, J = 8.0 Hz, 4H), 7.87 (d, J = 8.0 Hz, 4H), 7.44 (t, J = 8.0 Hz, 2H), 7.29 (d, J = 8.0 Hz, 4H), 6.82 (d, J = 8.0 Hz, 4H), 2.75 – 2.68 (m, 4H), 1.12 (d, J = 8.0 Hz, 24H). ^{13}C NMR (100 MHz, $CDCl_3$) δ (ppm): 163.64, 145.84, 140.94, 140.78, 139.23, 135.79, 132.87, 132.37, 130.88, 129.69, 129.39, 127.41, 127.37, 125.75, 124.46, 124.12, 122.79, 29.87, 29.27, 24.08. HRMS: m/z $[M+H]^+$ calcd for ($C_{68}H_{51}F_6N_2O_4$) 1073.3745; found 1073.3747 (error = 0.2 ppm).

3. FT-IR Spectrometer

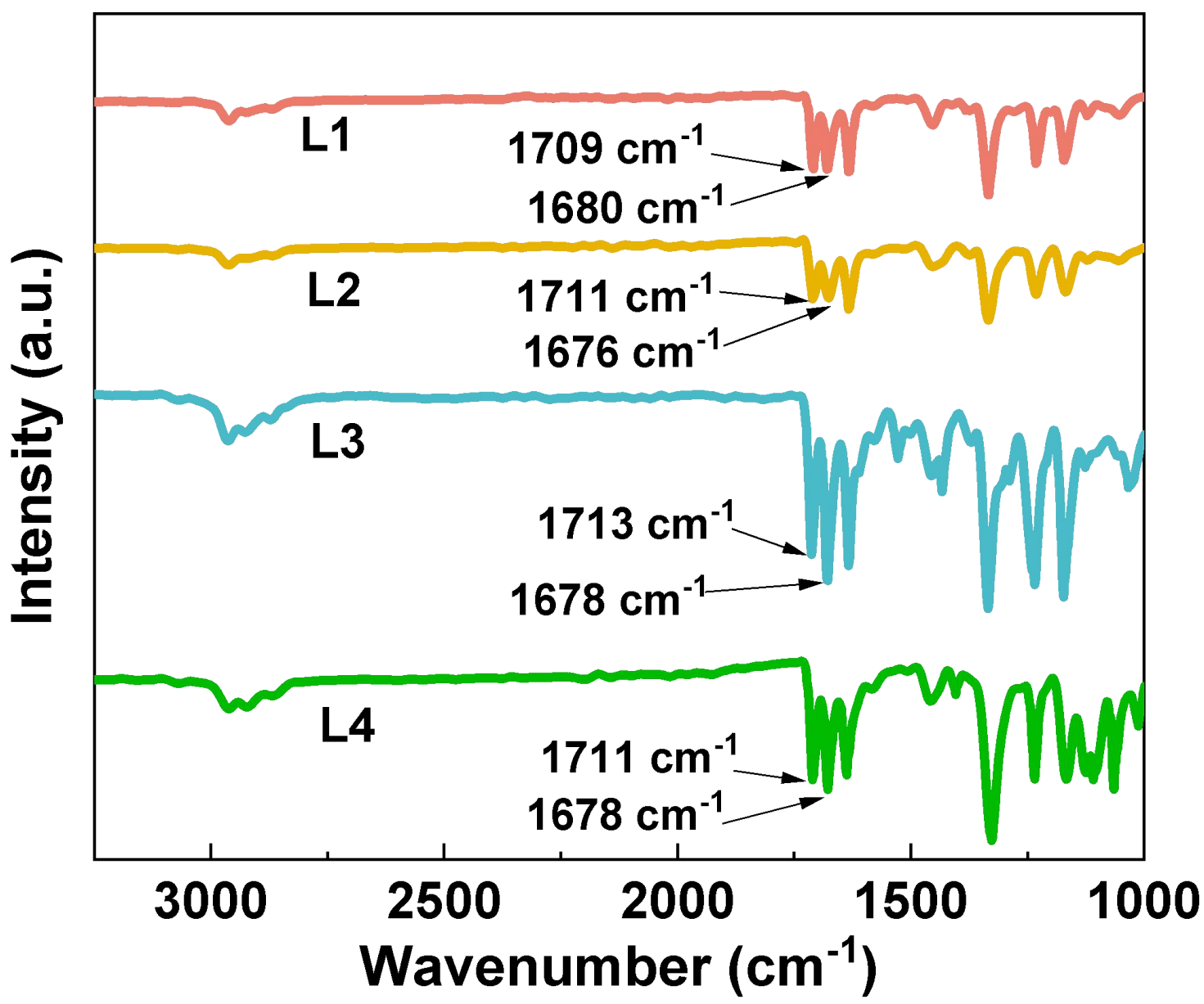

Figure S1. FT-IR spectra of **L1–L4** .

4. Single Crystal X-ray Crystallographic Data

The single crystals of **L1**–**L4** suitable for X-ray diffraction analysis were obtained by the slow evaporation of the corresponding mixed solutions. A crystal of **L1** was obtained from a mixed solution in dichloromethane/cyclohexane/1,4-dioxane (V: V: V = 5: 1: 0.5). Crystals of **L2** and **L4** were grown from the mixed solutions in chloroform/methanol/petroleum ether (V: V: V = 1: 1: 8), while **L3** was crystallized from a mixed solution in THF/*n*-butanol (V: V = 10: 1). Single-crystal X-ray measurements of **L1** were conducted on a XtaLAB AFC12 (RINC): Kappa single diffractometer, while those of **L2**–**L4** were carried out on a ROD, Synergy Custom system, HyPix-Arc 150 diffractometer. During data collection, the crystals of **L1**, **L2**, **L3**, and **L4** were kept at 149.99(10), 100.00(10), 100.02(10), and 100.3(5) K, respectively. Using Olex2[6], the structures were solved with the SHELXT[7-8] structure solution program using Intrinsic Phasing (**L1**) or Direct Methods (**L2**–**L4**), and all were refined with the SHELXL[8] refinement package using Least Squares minimisation. Crystallographic data of **L1**–**L4** have been deposited at the Cambridge Crystallographic Data Center, with the corresponding deposition numbers (CCDC) of 2511986, 2511982, 2511984, and 2511985. These data can be obtained free of charge from The Cambridge Crystallographic Data Centre via www.ccdc.cam.ac.uk/structures.

Table S1. Crystal data and structure refinement for **L1**.

Identification code	**L1**
Empirical formula	$C_{72}H_{68}N_2O_6S_2$
Formula weight	1121.40
Temperature/K	150
Crystal system	orthorhombic
Space group	$Pna2_1$
a/Å	15.0028(3)
b/Å	18.2461(5)
c/Å	22.1763(5)
α/°	90

β/°	90
γ/°	90
Volume/Å^3	6070.6(2)
Z	4
ρ_{calc}g/cm^3	1.227
μ/mm^{-1}	1.227
F(000)	2376.0
Crystal size/mm^3	0.14 × 0.12 × 0.1
Radiation	CuKα (λ = 1.54184)
2Θ range for data collection/°	6.272 to 143.892
Index ranges	-18 ≤ h ≤ 17, -22 ≤ k ≤ 20, -26 ≤ l ≤ 23
Reflections collected	20115
Independent reflections	8586 [R_{int} = 0.0366, R_{sigma} = 0.0529]
Data/restraints/parameters	8586/855/821
Goodness-of-fit on F^2	1.043
Final R indexes [I>=2σ (I)]	R_1 = 0.0704, wR_2 = 0.1956
Final R indexes [all data]	R_1 = 0.0847, wR_2 = 0.2146
Largest diff. peak/hole / e Å^{-3}	0.57/-0.40
Flack parameter	0.43(5)
CCDC number	2511986

Table S2. Crystal data and structure refinement for **L2**.

Identification code	**L2**
Empirical formula	$C_{66}H_{52}N_2O_4$
Formula weight	937.09
Temperature/K	100.00(10)
Crystal system	triclinic
Space group	P-1

a/Å	11.6447(5)
b/Å	14.2883(5)
c/Å	19.4870(6)
α/°	75.283(3)
β/°	89.982(3)
γ/°	67.259(4)
Volume/$Å^3$	2874.7(2)
Z	2
ρ_{calc}g/cm^3	1.083
μ/mm^{-1}	0.524
F(000)	988.0
Crystal size/mm^3	0.15 × 0.12 × 0.11
Radiation	Cu Kα (λ = 1.54184)
2Θ range for data collection/°	7.268 to 133.202
Index ranges	-13 ≤ h ≤ 13, -16 ≤ k ≤ 14, -23 ≤ l ≤ 23
Reflections collected	43493
Independent reflections	9840 [R_{int} = 0.0278, R_{sigma} = 0.0290]
Data/restraints/parameters	9840/422/734
Goodness-of-fit on F^2	1.062
Final R indexes [I>=2σ (I)]	R_1 = 0.0724, wR_2 = 0.1856
Final R indexes [all data]	R_1 = 0.0849, wR_2 = 0.1932
Largest diff. peak/hole / e $Å^{-3}$	0.32/-0.28
CCDC number	2511982

Table S3. Crystal data and structure refinement for **L3**.

Identification code	**L3**
Empirical formula	$C_{68}H_{56}N_2O_6$
Formula weight	997.14

Temperature/K	100.02(10)
Crystal system	monoclinic
Space group	C2/c
a/Å	25.0366(4)
b/Å	15.3998(2)
c/Å	13.6960(2)
α/°	90
β/°	95.333(2)
γ/°	90
Volume/Å^3	5257.75(13)
Z	4
ρ_{calc}g/cm^3	1.260
μ/mm^{-1}	0.633
F(000)	2104.0
Crystal size/mm^3	0.14 × 0.11 × 0.1
Radiation	Cu Kα (λ = 1.54184)
2Θ range for data collection/°	9.13 to 145.942
Index ranges	-31 ≤ h ≤ 29, -18 ≤ k ≤ 16, -16 ≤ l ≤ 16
Reflections collected	18090
Independent reflections	5067 [R_{int} = 0.0341, R_{sigma} = 0.0281]
Data/restraints/parameters	5067/0/348
Goodness-of-fit on F^2	1.089
Final R indexes [I>=2σ (I)]	R_1 = 0.0577, wR_2 = 0.1459
Final R indexes [all data]	R_1 = 0.0637, wR_2 = 0.1503
Largest diff. peak/hole / e Å^{-3}	0.45/-0.28
CCDC number	2511984

Table S4. Crystal data and structure refinement for **L4**.

Identification code	**L4**
Empirical formula	$C_{68}H_{50}F_6N_2O_4$
Formula weight	1073.10
Temperature/K	100.3(5)
Crystal system	monoclinic
Space group	C2/c
a/Å	18.7752(3)
b/Å	19.4826(2)
c/Å	15.6512(2)
α/°	90
β/°	95.2015(13)
γ/°	90
Volume/Å^3	5701.50(13)
Z	4
ρ_{calc}g/cm^3	1.250
μ/mm^{-1}	0.750
F(000)	2232.0
Crystal size/mm^3	0.14 × 0.12 × 0.09
Radiation	Cu Kα (λ = 1.54184)
2Θ range for data collection/°	8.384 to 146.312
Index ranges	$-22 \leq h \leq 23$, $-14 \leq k \leq 22$, $-18 \leq l \leq 19$
Reflections collected	24060
Independent reflections	5457 [R_{int} = 0.0305, R_{sigma} = 0.0218]
Data/restraints/parameters	5457/39/386
Goodness-of-fit on F^2	1.044
Final R indexes [I>=2σ (I)]	R_1 = 0.0466, wR_2 = 0.1247
Final R indexes [all data]	R_1 = 0.0522, wR_2 = 0.1288
Largest diff. peak/hole / e Å^{-3}	0.34/-0.24

CCDC number	2511985

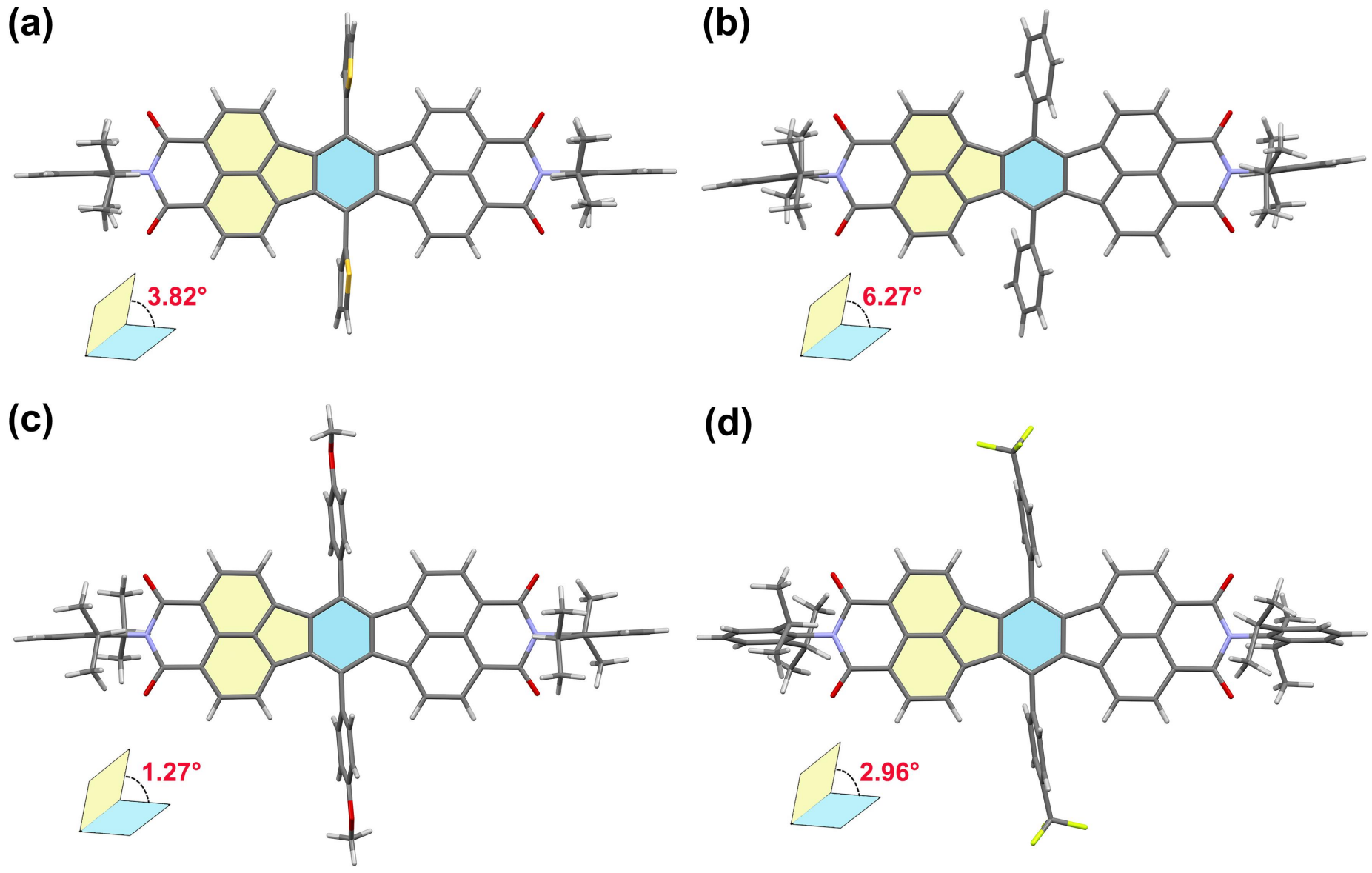

Figure S2. Single-crystal structures of (a) **L1**, (b) **L2**, (c) **L3**, and (d) **L4**.

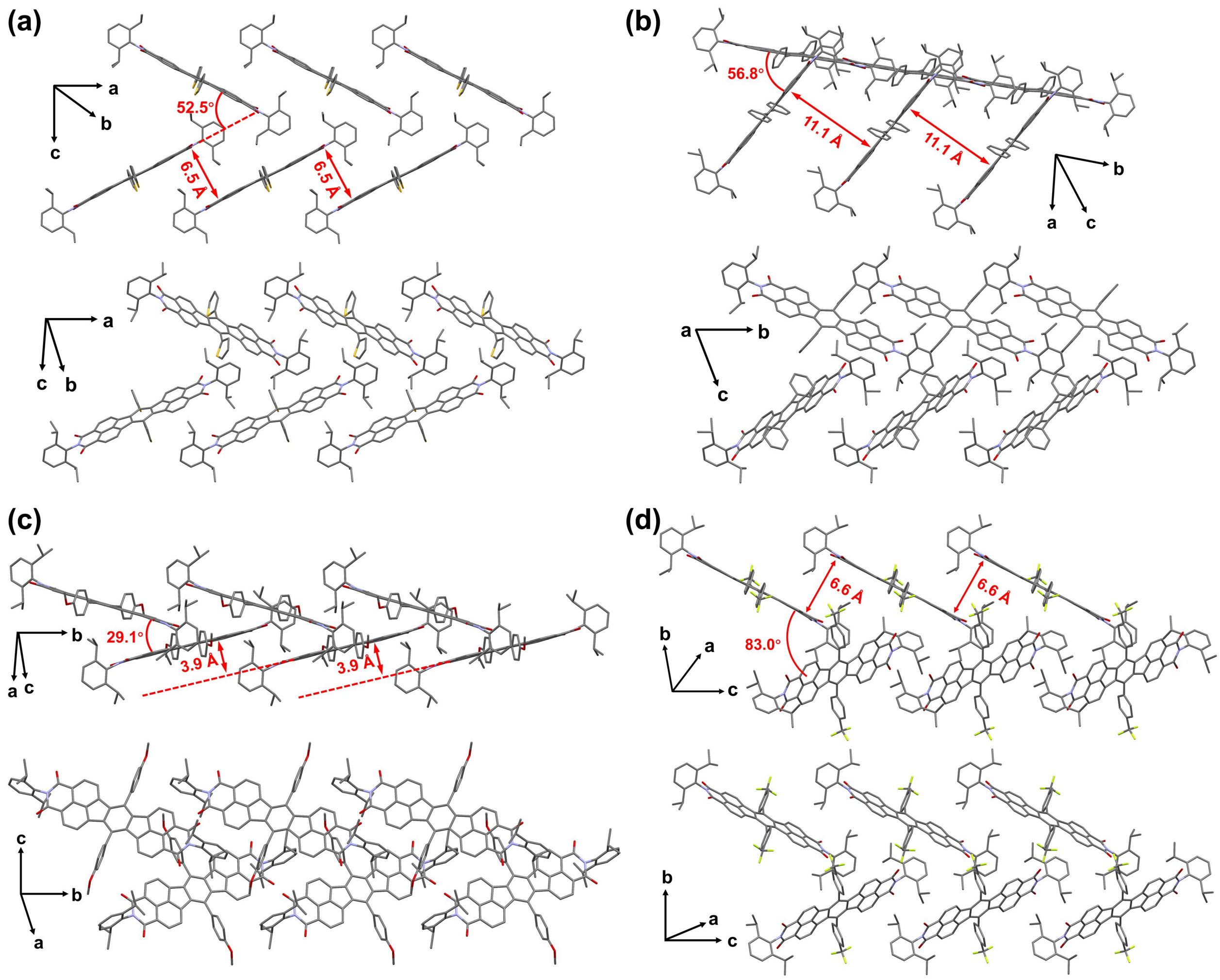

Figure S3. Crystal packing structures of (a) **L1**, (b) **L2**, (c) **L3**, and (d) **L4**.

5. Photophysical and Electrochemical Properties

Table S5. Photophysical/Electrochemical properties and DFT-calculated energy levels of **L1**–**L4**.

Sample	λ_{sol}^{max} [a] [nm]	λ_{sol}^{onset} [a] [nm]	E_g^{opt} [a] [eV]	λ_{sol}^{em} [b] [nm]	E_{red}^{onset} [V]	E_{LUMO} [c] [eV]	E_{HOMO} [d] [eV]	E_{LUMO} [e] [eV]	E_{HOMO} [e] [eV]	E_g^{DFT} [d] [eV]
**L1**	475	502	2.47	620	–0.61	–3.81	–6.28	–3.30	–6.28	2.98
**L2**	481	508	2.44	625	–0.60	–3.82	–6.26	–3.24	–6.18	2.94
**L3**	485	521	2.38	627	–0.65	–3.77	–6.15	–3.18	–6.07	2.89
**L4**	474	496	2.50	617	–0.57	–3.85	–6.35	–3.45	–6.39	2.95

[a] Measured in 5.50 × 10^{-6} M chloroform solution. [b] Measured in 2.75 × 10^{-6} M chloroform solution. [c] Estimated from $E_{LUMO} = -(E_{red}^{onset} + 4.42)$ eV. [d] $E_{HOMO} = E_{LUMO} - E_g$ eV. [e] Estimated from DFT calculations at the B3LYP-D3(BJ)/6-31G (d) level.

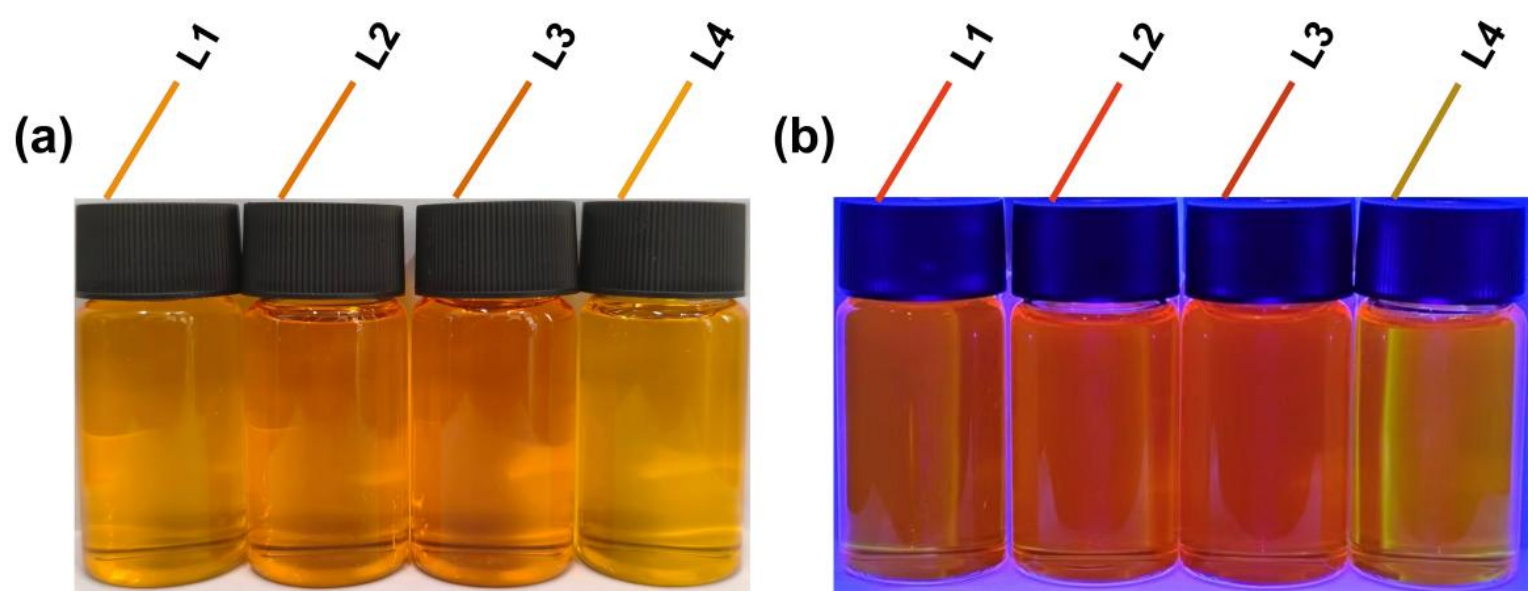

Figure S4. (a) The color of **L1–L4** in chloroform. (b) Photoluminescence photographs of **L1–L4** in chloroform taken under 365 nm UV irradiation.

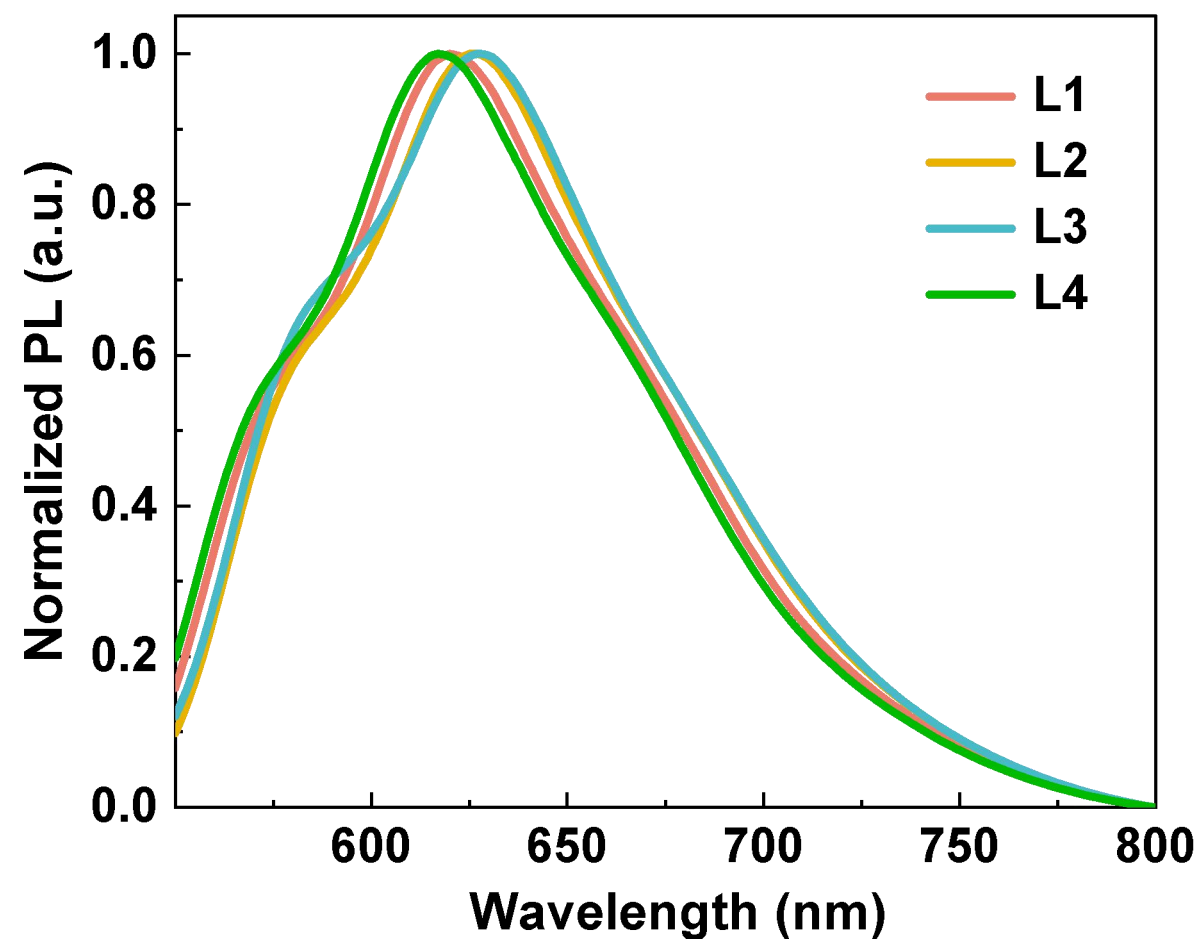

Figure S5. Normalized photoluminescence spectra of **L1–L4** in chloroform (2.75 × 10^{-6} M).

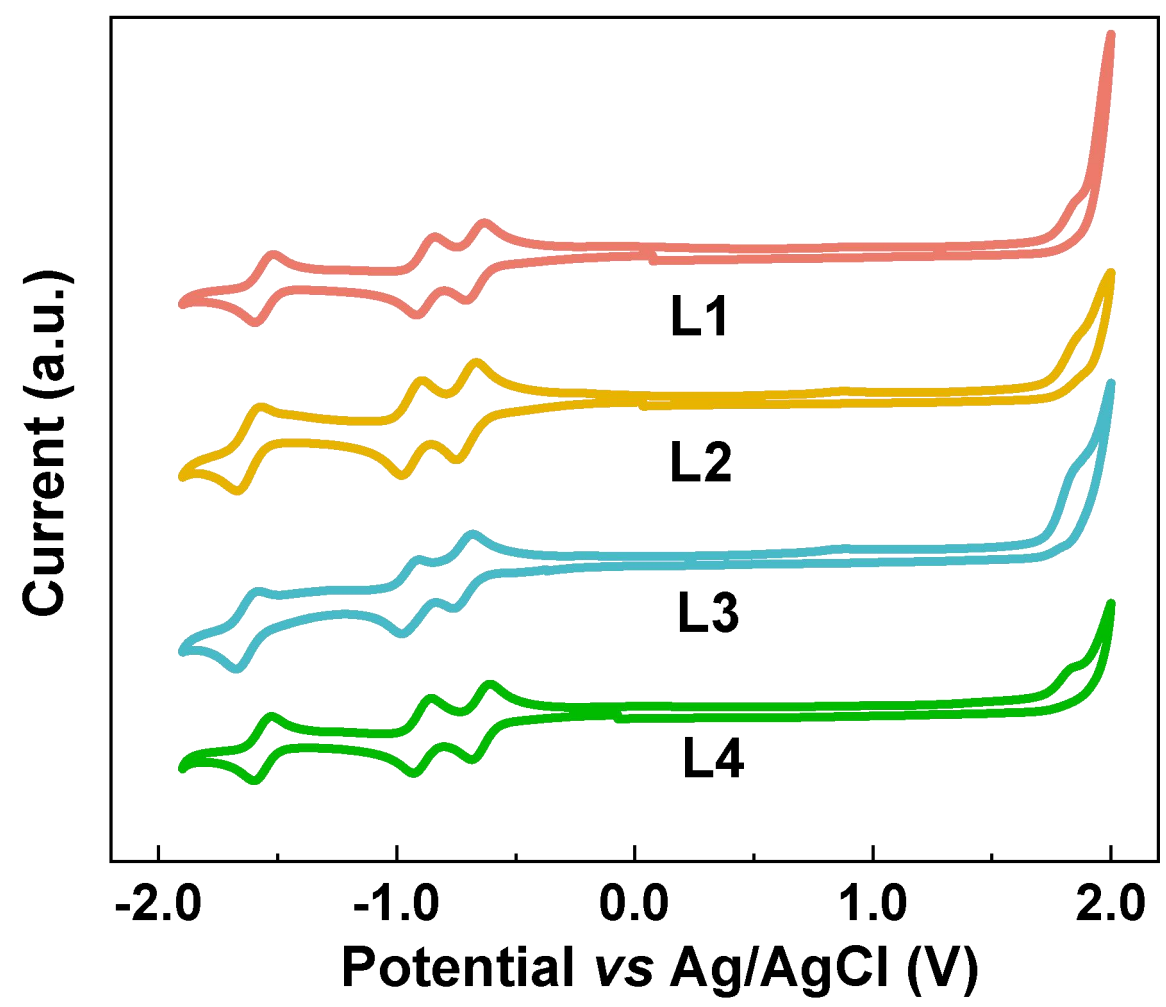

Figure S6. Cyclic voltammetry profiles of **L1–L4** collected in dichloromethane with the scan voltage window ranging from –2.0 to 2.0 V.

6. Theoretical Calculations

To understand the electronic structures and absorption spectra of **L1–L4** in depth, density functional theory (DFT) and time-dependent DFT (TD-DFT) calculations were conducted by using Gaussian 16 program[9]. The ground-state geometries and frequency analysis for each molecular model (also named as **L1–L4)** were obtained at the level of the B3LYP-D3(BJ)/6-31G(d)[10], while all the alkyl chains were simplified to be methyl groups. Basing on the optimized ground-state geometries, the absorption spectra of **L1–L4** were optimized via TD-DFT calculations at the PBE0/Def2-SVP level[11]. The solvent effect of chloroform ($\varepsilon = 4.71$) was taken into account by a polarizable continuum model (PCM)[12]. Excited-state analysis was processed with the TD-DFT results by using Multiwfn 3.8 program according to the program manual and literature method[13].

To evaluate the aromaticity of **L1–L4**, the nucleus independent chemical shift (NICS)[14-15] and anisotropy of the induced current density (AICD) plots[16] were computed on the basis of the ground-state geometries optimized at the level of the B3LYP-D3(BJ)/6-31G(d). Both calculation methods had been benchmarked and widely used in evaluating aromatic criteria. The $NICS(1.7)_{ZZ}$ values were calculated by adopting the ghost atoms placed at 1.7 Å above the center of each ring plane and using the gauge-independent atomic orbital (GIAO) method at the B3LYP/6-311+G(d,p) level[14-15]. The $NICS(1.7)_{XY}$ values were calculated with the gauge-independent atomic orbital (GIAO) method at the B3LYP/6-311+G(d,p) level by scanning the path from ring A to ring D (For the details, see Figure X). The calculation was conducted by adopting the ghost atoms placed 1.7 Å above the molecular plane, spanning from the nitrogen atom in ring A to the center of ring D. The 2D-NICS maps were calculated with the gauge-independent atomic orbital (GIAO) method at the B3LYP/6-311G(d,p) level. The AICD plots were calculated at the B3LYP/6-311+G(d,p) level and then generated with the AICD 2.0.0 program at 0.035 a.u. isosurface[16].

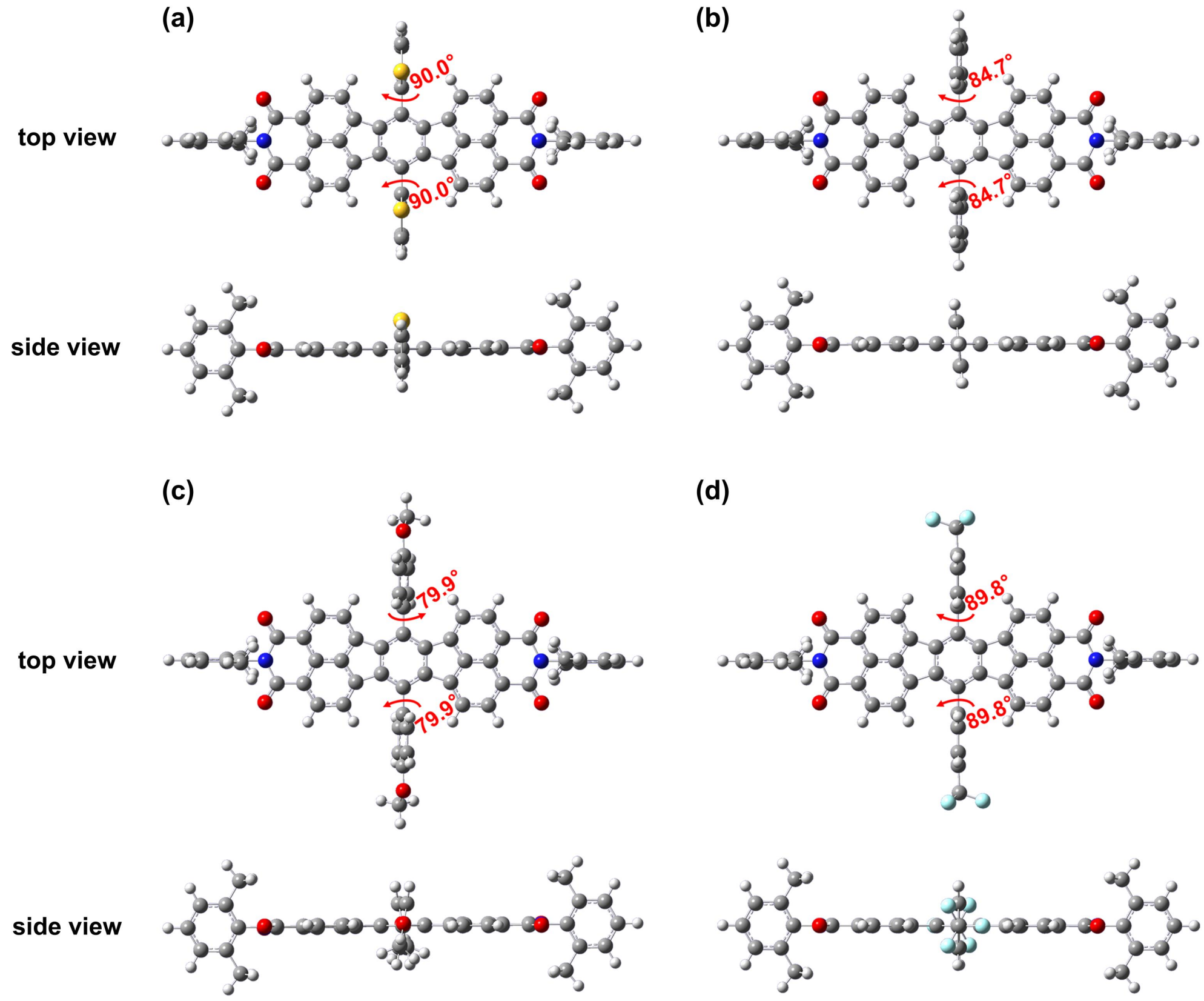

Figure S7. Ground-state structures and twisted dihedral angles of (a) **L1**, (b) **L2**, (c) **L3**, and (d) **L4** calculated at the level of B3LYP-D3(BJ)/6-31G(d).

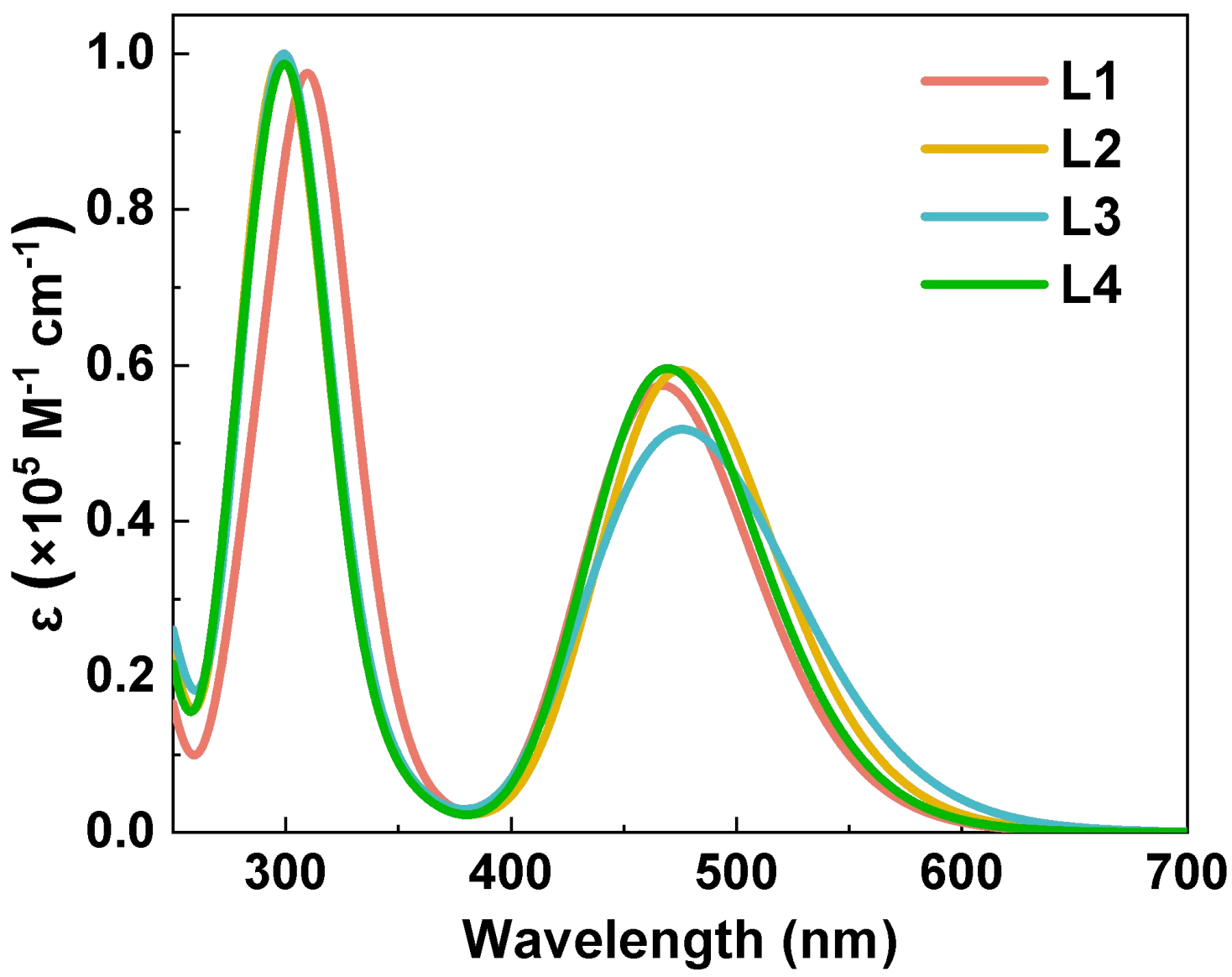

Figure S8. Absorption spectra of **L1-L4** calculated at PBE0/def2-SVP by TD-DFT treatment in chloroform solution.

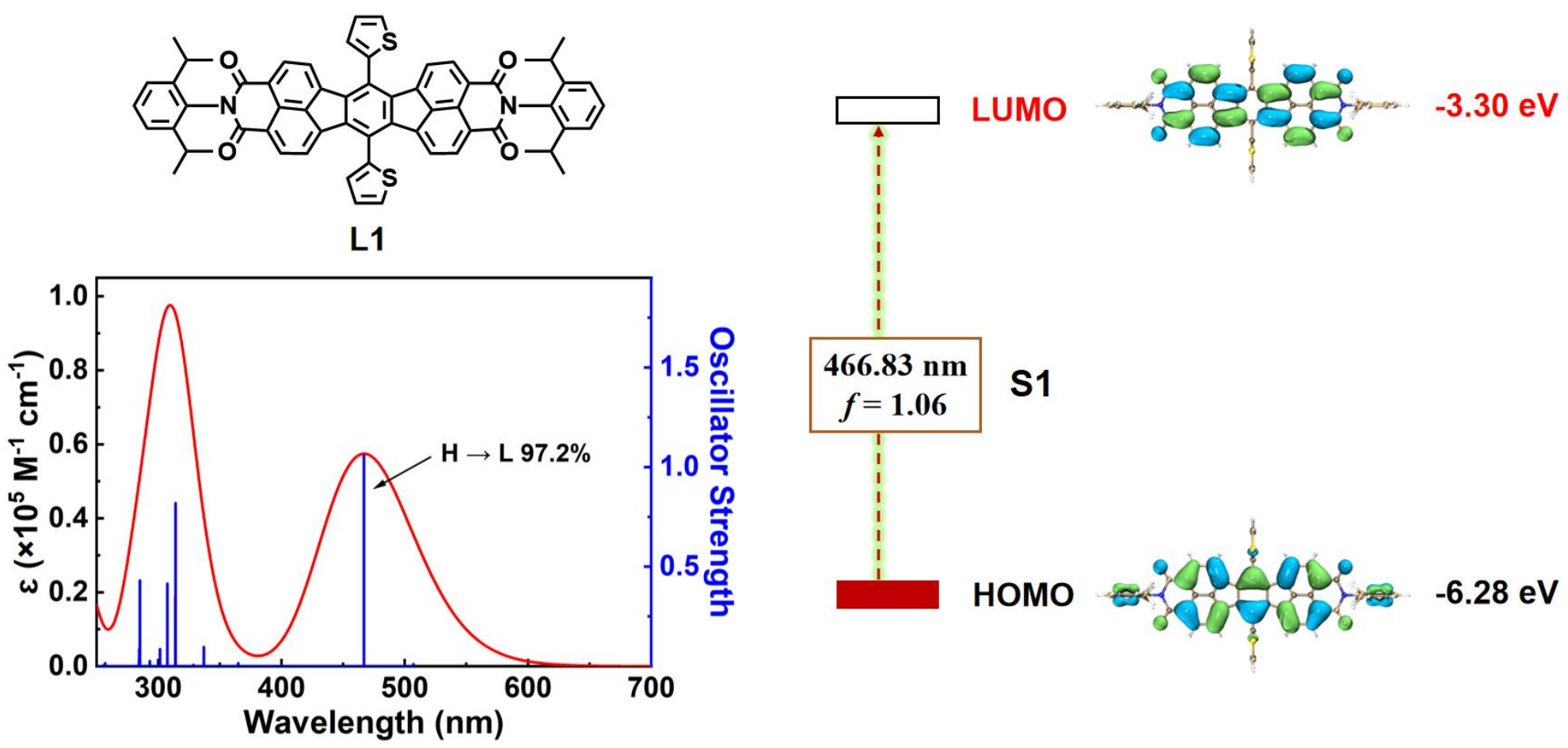

Figure S9. Absorption spectra and typical electron transitions of **L1** evaluated at PBE0/def2-SVP by TD-DFT treatment in chloroform solution (H represents HOMO, and L represents LUMO).

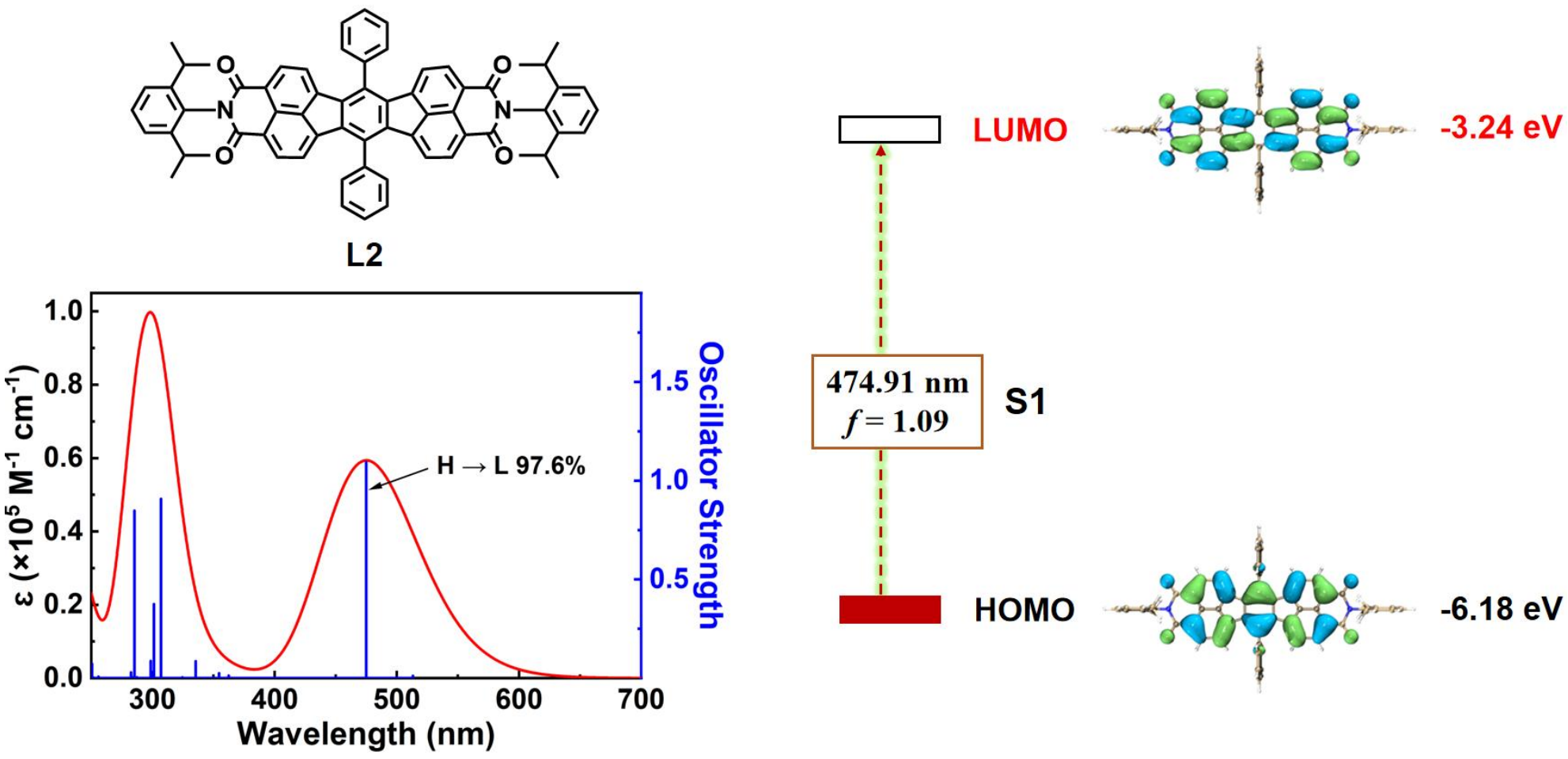

Figure S10. Absorption spectra and typical electron transitions of **L2** evaluated at PBE0/def2-SVP by TD-DFT treatment in chloroform solution.

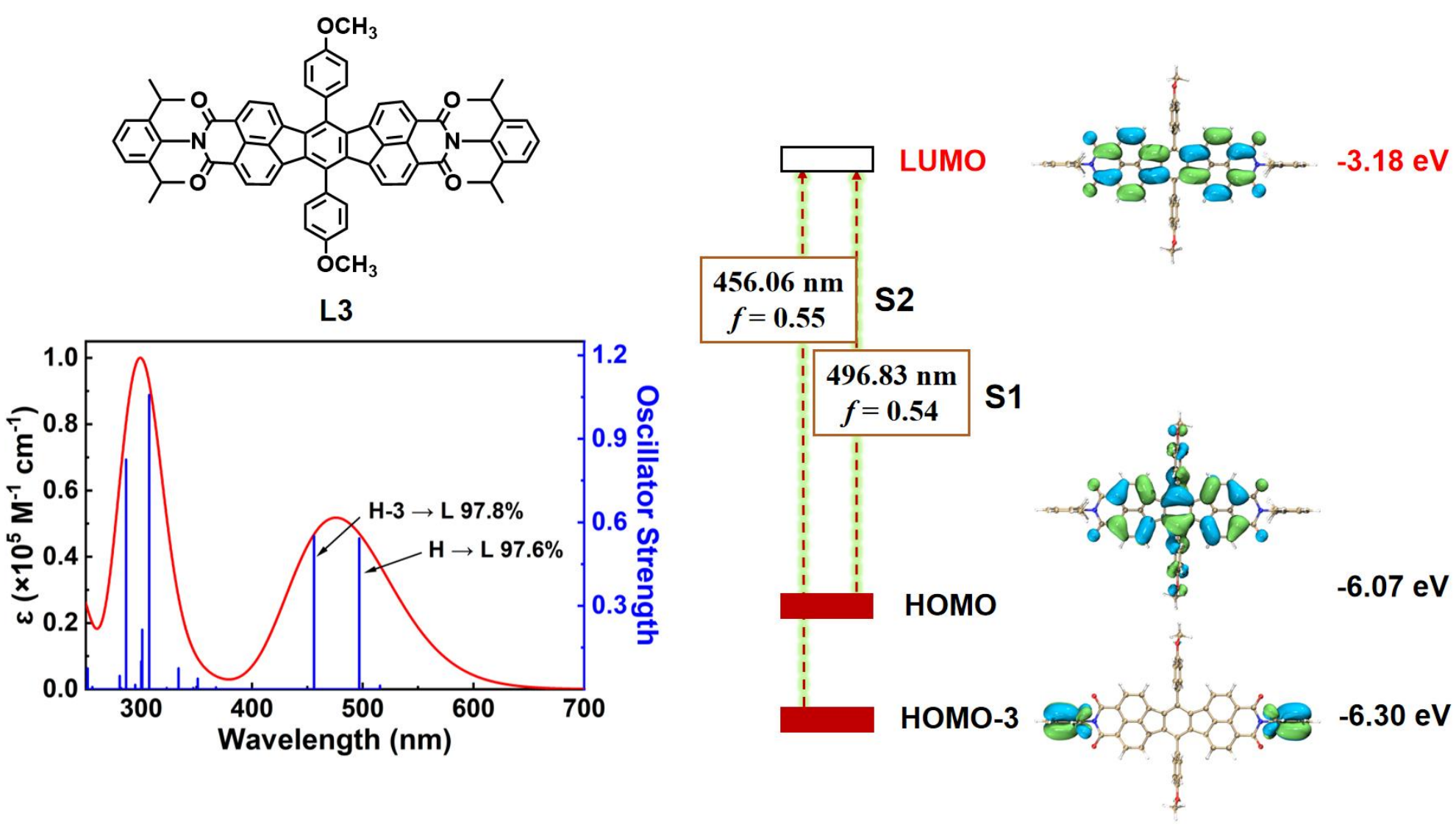

Figure S11. Absorption spectra and typical electron transitions of **L3** evaluated at PBE0/def2-SVP by TD-DFT treatment in chloroform solution.

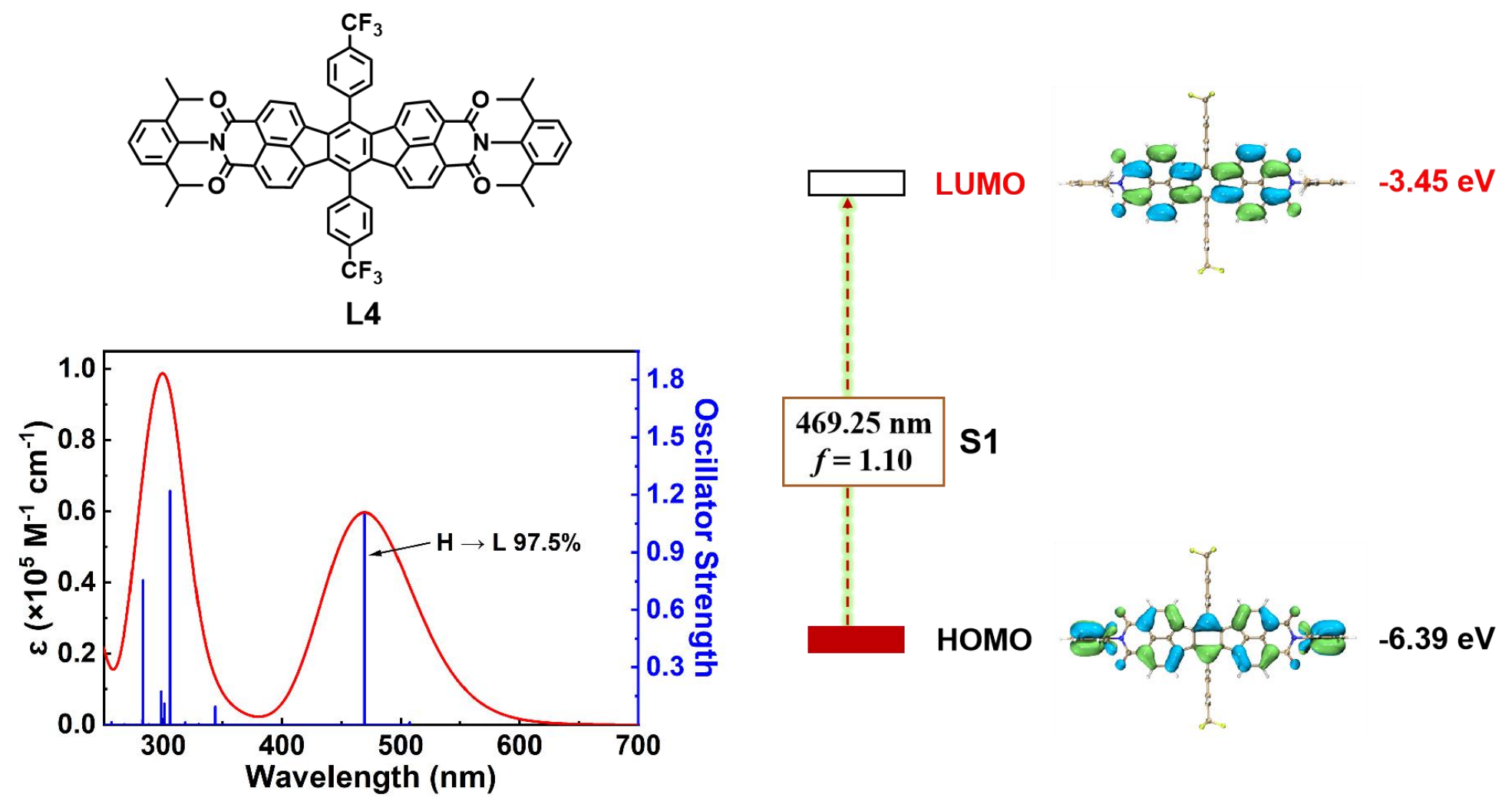

Figure S12. Absorption spectra and typical electron transitions of **L4** evaluated at PBE0/def2-SVP by TD-DFT treatment in chloroform solution.

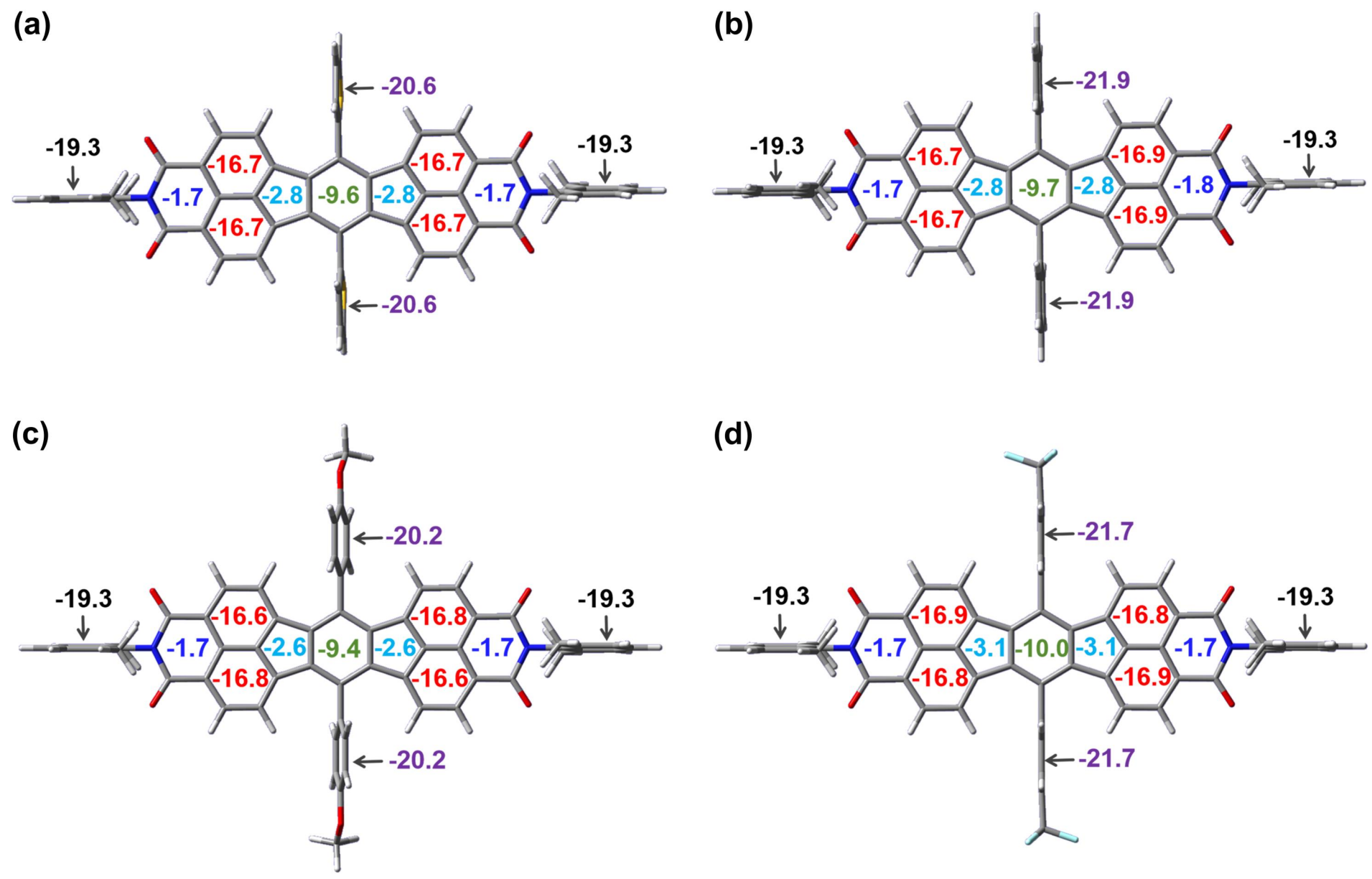

Figure S13. Calculated NICS(1.7)$_{ZZ}$ values of (a) **L1**, (b) **L2**, (c) **L3**, and (d) **L4** at the level of GIAO-B3LYP/6-311+G (d, p).

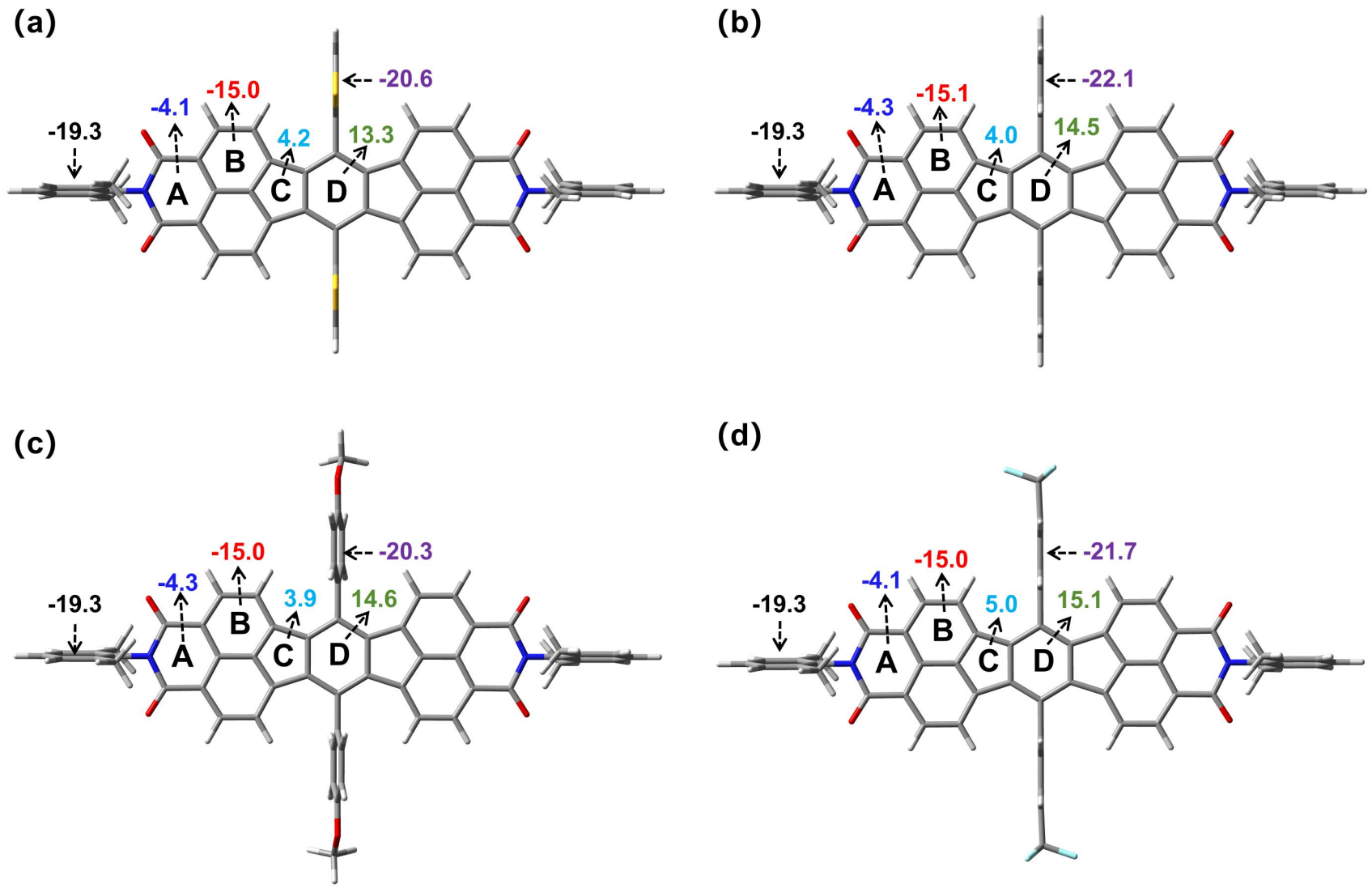

Figure S14. Calculated NICS(1.7)$_{ZZ}$ values of the low-lying triplet excited state (T_1) of (a) L1, (b) L2, (c) L3, and (d) L4.

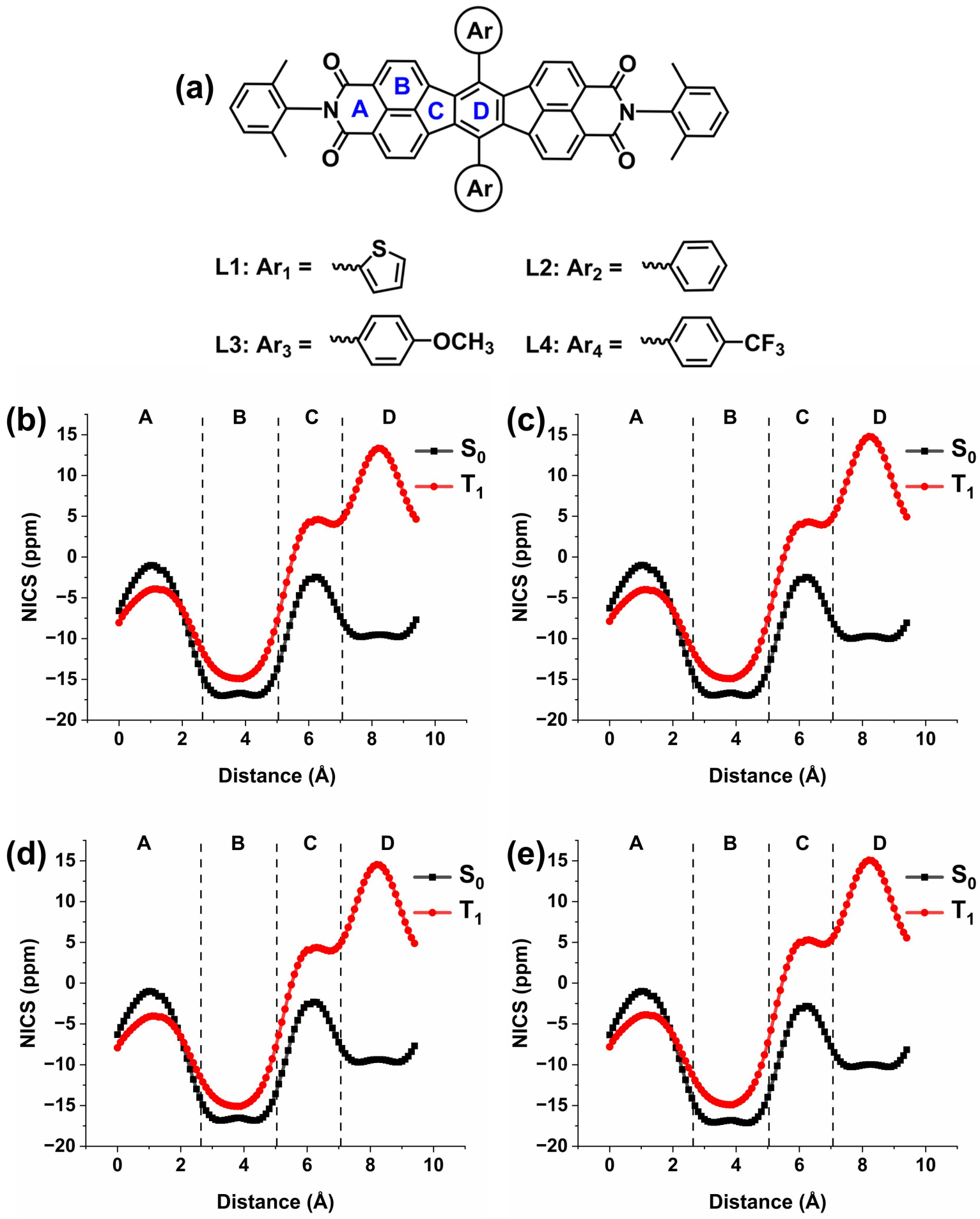

Figure S15. Comparison of the calculated NICS(1.7)$_{ZZ}$ XY-scan values between T_1 and S_0 of (a) L1, (b) L2, (c) L3, and (d) L4.

7. Z-Scan Experiments

To characterize the NLO performance of **L1-L4**, the Z-scan experimental system[17] (Figure S16) was set up with a Q-switched Nd:YAG laser, and a 532 nm laser pulse with 8 ns pulses width and repetition frequency of 10 Hz was used. The sample was loaded onto the platform, which drove by a stepper-motor and moved accurately over a distance of ± 45 mm from the laser focal position (Z = 0) along with the optical z-axis. The Z-scan curves, which are the normalized transmittance (T_{norm}) function of the samples position (Z) relative to the laser focal position, can be obtained from Z-scan experiment.

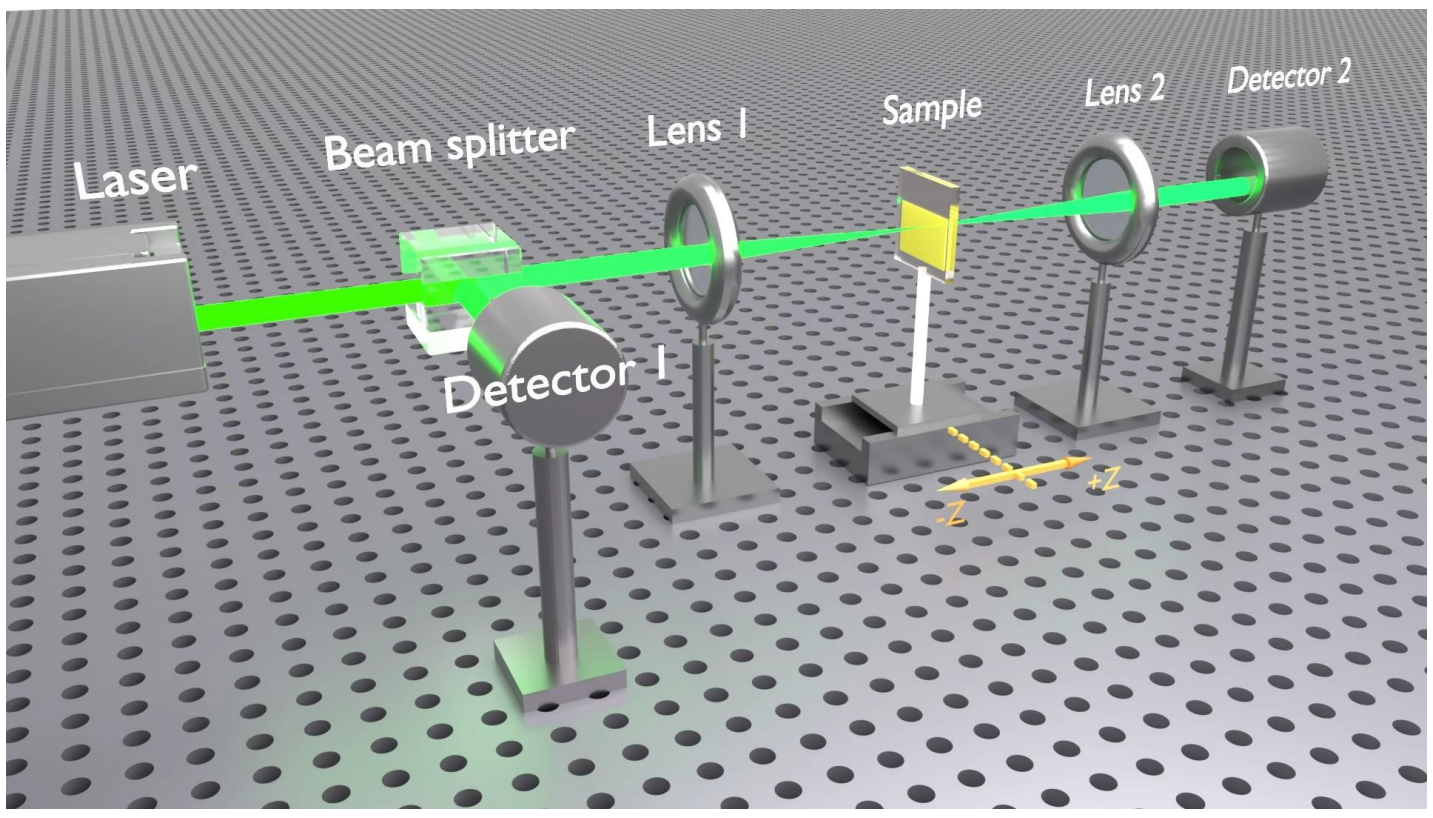

Figure S16. The schematic diagram of the Z-scan technology upon the treatment of a 532 nm pulsed laser.

The effective nonlinear absorption coefficient (β_{eff}) can be fitted by the following equation (1-2)[18].

$$T\,(z, s=1) = \sum_{m=0}^{\infty} \frac{[-q_0(z,0)]^m}{(m+1)^{3/2}} \approx 1 - \frac{\beta_{\mathrm{eff}} I_0 L_{\mathrm{eff}}}{2\sqrt{2}\left(1+z^2/z_0^2\right)} \quad (m \in N) \qquad (1)$$

Where q_0 can be estimated by formula (2)

$$q_0(z,0) = \frac{\beta_{\mathrm{eff}} I_0 L_{\mathrm{eff}}}{1+z^2/z_0^2} \qquad (2)$$

In equations (1) and (2), z is the distance of the sample from laser focus (Z = 0), $z_0 = \pi\omega_0{}^2/\lambda$ is the Rayleigh range, which is calculated to be 1.08 mm with the beam waist radius of $\omega_0 = 13.5$ μm at laser focal position. I_0 is the input intensity of the laser beam at focal point, $L_{eff} = (1-e^{-\alpha L})/\alpha$ is the effective length of the sample, where $\alpha = (-\ln T_{\mathrm{lin}})/L$ is the linear absorption coefficient at the given wavelength, and L is the sample thickness of sample.

For a spatially Gaussian beam, the relationship of the T_{norm} and the input fluence (F_{in}) can be

fitted using open-aperture Z-scan data. The F_{in} at Z position can be calculated from corresponding beam radius ω and the input laser energy E_{in} by using expression (3).

$$F_{in}(z) = \frac{4\sqrt{\ln 2}E_{in}}{\pi^{3/2}\omega_z{}^2} \qquad (3)$$

Where the beam radius (ω_z) can be given by relation (4)

$$\omega_z = \omega_0 \sqrt{1 + (z/z_0)^2} \qquad (4)$$

Table S6. The Z-scan experiments of **L1–L4** and **C_{60}** in *o*-dichlorobenzene with an identical linear transmittance of 60% at 532 nm.

Samples	E_{in} (μJ)[a]	T_{min}[b]	σ_{ex}/σ_0[c]	F_{on} (mJ cm^{-2})[d]	F_{th} (J cm^{-2})[e]	β_{eff} (cm GW^{-1})[f]
C_{60}	5	0.50	1.84	26	1.049	407
L1		0.28	2.34	7	0.123	941
L2		0.25	2.41	7	0.150	885
L3		0.44	1.83	8	0.559	698
L4		0.41	2.66	8	0.195	668
C_{60}	10	0.40	2.04	30	0.935	271
L1		0.22	2.56	10	0.146	610
L2		0.24	2.56	10	0.179	571
L3		0.45	1.88	14	1.375	348
L4		0.26	2.89	11	0.212	527
C_{60}	15	0.36	2.23	25	0.882	225
L1		0.15	3.07	6	0.125	557
L2		0.15	2.99	8	0.151	506
L3		0.41	1.95	10	1.018	321
L4		0.17	3.44	9	0.177	469

[a] Input laser energy. [b] Normalized transmittance at Z = 0. [c] The ratio of the effective excited-state absorption cross-section (σ_{ex}) and the ground-state absorption cross-section (σ_0). [d] The input fluence corresponds to the normalized transmittance of 0.95. [e] The input fluence corresponds to the normalized transmittance of 0.50. [f] Nonlinear extinction coefficient. All samples were placed in a quartz cuvette with the thickness of 1 mm.

Table S7. Comparison of the optical-limiting performance parameters between our developed diimides (**L1–L4**) and some well-known optical-limiting materials reported in literature.

Sample	Parameters	T_{min} (%)[a]	β (cm GW^{-1})[b]	F_{on} (J cm^{-2})[c]	F_{th} (J cm^{-2})[d]	Ref.
**L1**	8 ns, 10 Hz, 5 μJ	28	941	0.007	0.123	***This work***
**L2**	8 ns, 10 Hz, 5 μJ	25	885	0.007	0.150	***This work***
**L3**	8 ns, 10 Hz, 5 μJ	44	698	0.008	0.559	***This work***
**L4**	8 ns, 10 Hz, 5 μJ	41	668	0.008	0.195	***This work***
**C60**	8 ns,10 Hz, 5 μJ	50	407	0.026	1.049	***This work***
**PPor3-MWCNT**	7 ns, 10 Hz, 5 μJ	65	150	0.66	0.98	*Opt. Mater.*, **2022**, *123*, 111913.
**LaPc-4(C_{60})**	4 ns, 10 Hz, 18 μJ	75	8.4	*ca.* 2	*ca.* 9.5	*J. Phys. Chem. C*, **2023**, *127*, 4258.
**PDPP-TTzPt**	8 ns, 10 Hz, 10 μJ	83	36	*ca.* 3	*ca.* 13	*ACS Appl. Mater. Interfaces*, **2020**, *12*, 2944.
**ITIC-5**	8 ns, 10 Hz, 10 μJ	62	162	0.041	*ca.* 3	*ACS Appl. Opt. Mater.*, **2023**, *1*, 1586.
**CuPc-PI-COF-1**	—, 14 μJ	47	33	0.61	9.55	*Adv. Funct. Mater.*, **2024**, *34*, 2404289.
**NNNR-2**	4 ns,10 Hz, 15 μJ	36	374	0.013	0.5	*Angew. Chem. Int. Ed.*, **2023**, 62, e202306418.
**BDFTI**	8 ns,10 Hz, 30 μJ	50	51	0.34	/	*Chem. Eur. J.*, **2025**, 31, e202403332.
**Pt-2b**	4 ns,10 Hz, 100 μJ	10	965	0.037	0.26	*Inorg. Chem. Front.*, **2025**, 12, 6972.
**TTF-Pt(bzimb)**	8 ns,10 Hz, 6μJ	68	156	0.11	/	*J. Mater. Chem. C*, **2018**, *6*, 8495.
**P2-Pt**	8 ns,10 Hz, 15 μJ	59	78	0.16	/	*Macromolecules*, **2024**, *57*,

						1030.
**P2**	8 ns,10 Hz, 30 μJ	54	47	0.53	10.7	*ACS Macro Lett.*, **2025**, *14*, 1081.
**5b**	8 ns,10 Hz, 10 μJ	71	88	0.14	/	*ACS Appl. Mater. Interfaces*, **2025**, *17*, 47323.
**[Hg-T(C_8-F)T-Hg]$_1$**	10 ns,10 Hz, —	60	/	*ca.* 0.02	0.31	*Adv. Funct. Mater.*, **2007**, *17*, 963.
**B-Pt-B**	10 ns,10 Hz, —	45	/	*ca.* 0.03	0.68	*Adv. Funct. Mater.*, **2009**, *19*, 531.
**N-Pt-O**	10 ns,10 Hz, —	40	/	*ca.* 0.02	0.33	*Adv. Funct. Mater.*, **2009**, *19*, 531.
**GNSs**	8 ns, 1Hz, 250 μJ	55	5.4	*ca.* 0.06	2.3	*J. Mater. Chem. C*, **2015**, *3*, 9948.
**GO-Pt-2**	4 ns, 10 Hz, —	69	77.3	—	0.3	*ACS Appl. Mater. Interfaces*, **2017**, *9*, 33029.
**FGO**	5 ns, —, 30 μJ	25	140	—	0.8	*J. Phys. Chem. C*, **2012**, *116*, 25955.
**GO-PcZn**	6 ns, 10 Hz, —	53	51	—	*ca.* 6.5	*Carbon*, **2011**, *49*, 1900.
**SWCNT-2**	10 ns, —	25	300	—	0.21	*J. Phys. Chem. C*, **2014**, *118*, 7057.

[a]Normalized transmittance at Z = 0. [b]Nonlinear extinction coefficient. [c]The input fluence corresponds to the normalized transmittance of 0.95. [d]The input fluence corresponds to the normalized transmittance of 0.50.

8. ^{1}H and ^{13}C NMR Spectra of the Novel Compounds

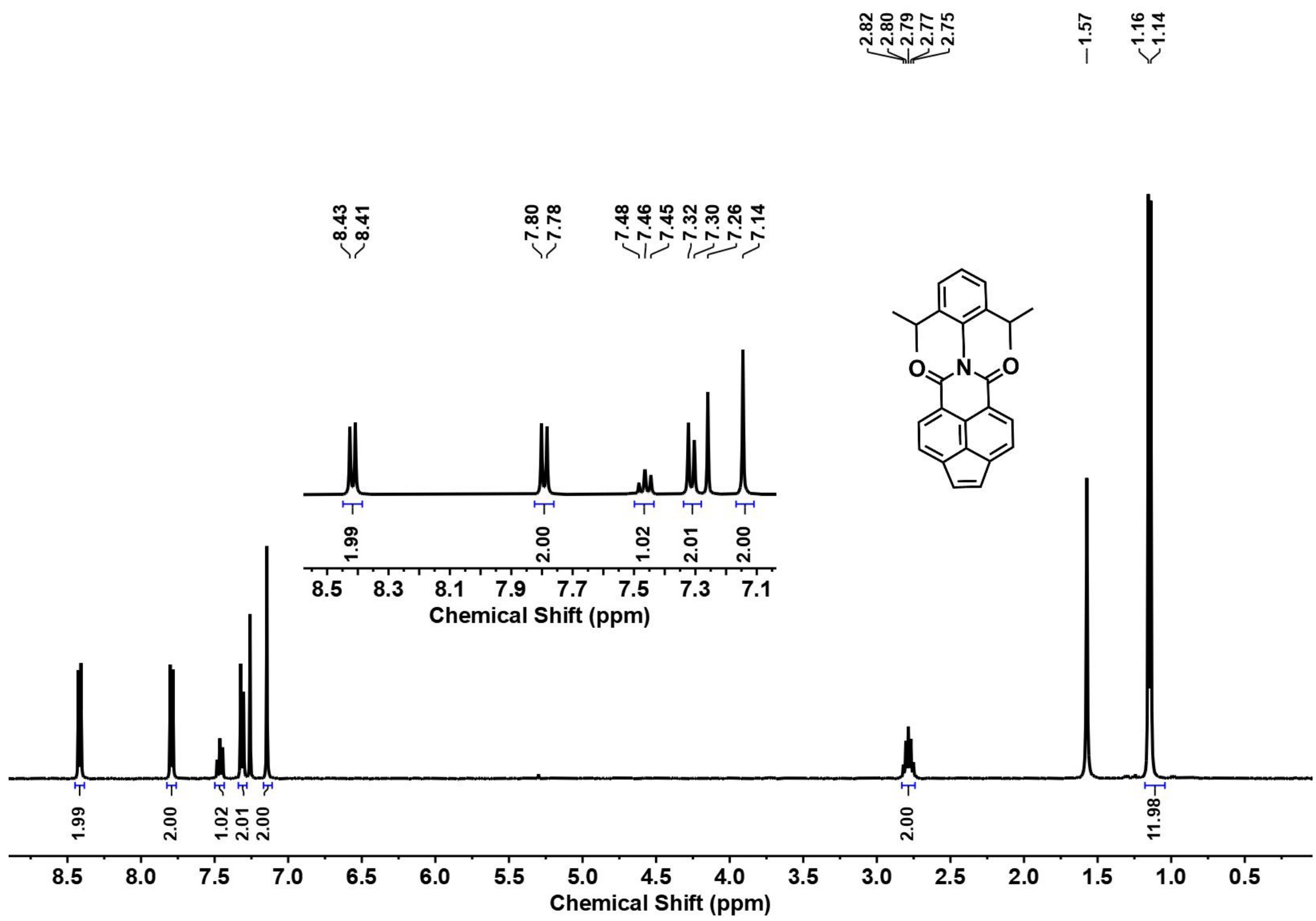

Figure S17. ^{1}H NMR spectra of compound **6a** in $CDCl_3$ (298 K).

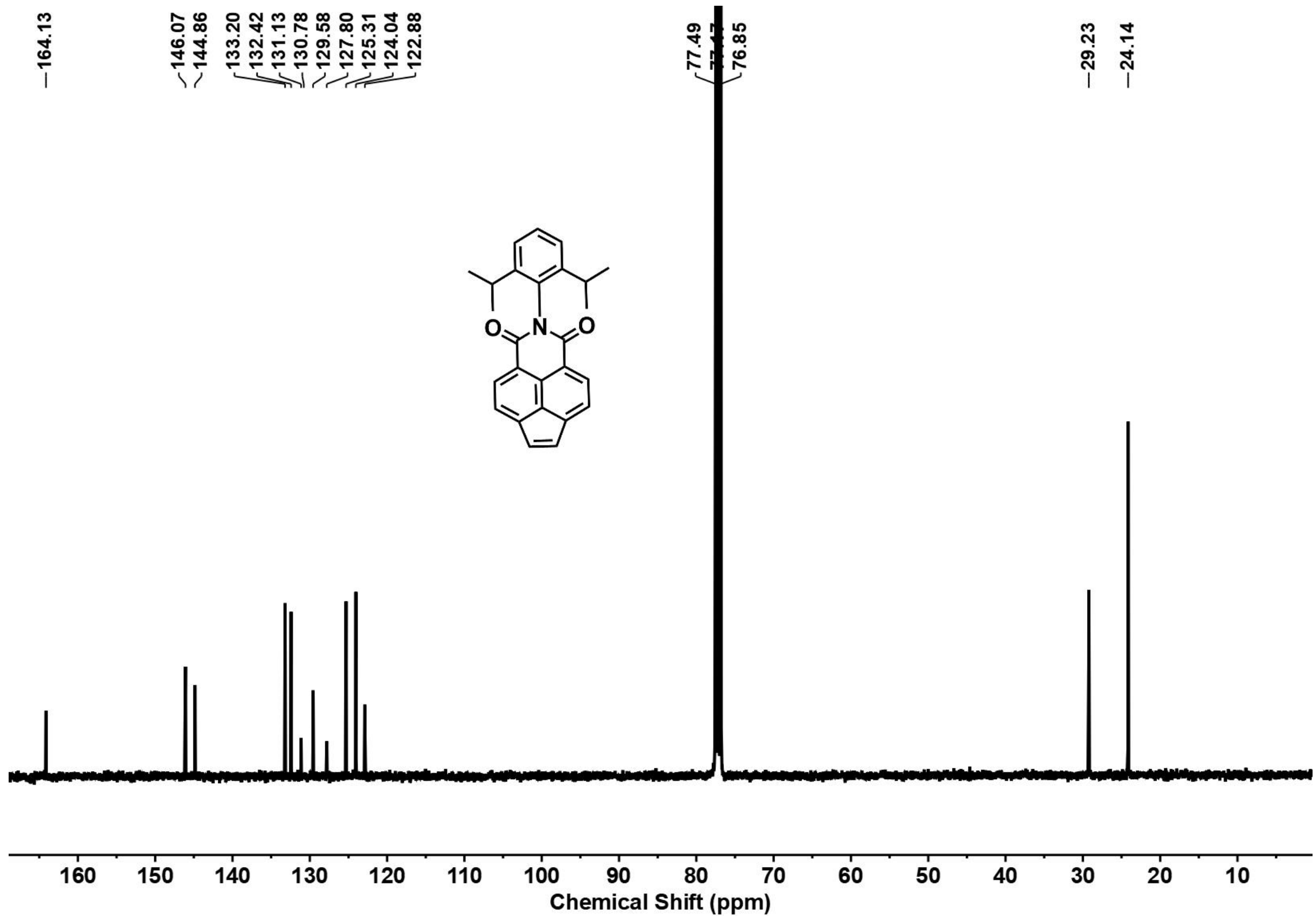

Figure S18. ^{13}C NMR spectra of compound **6b** in $CDCl_3$ (298 K).

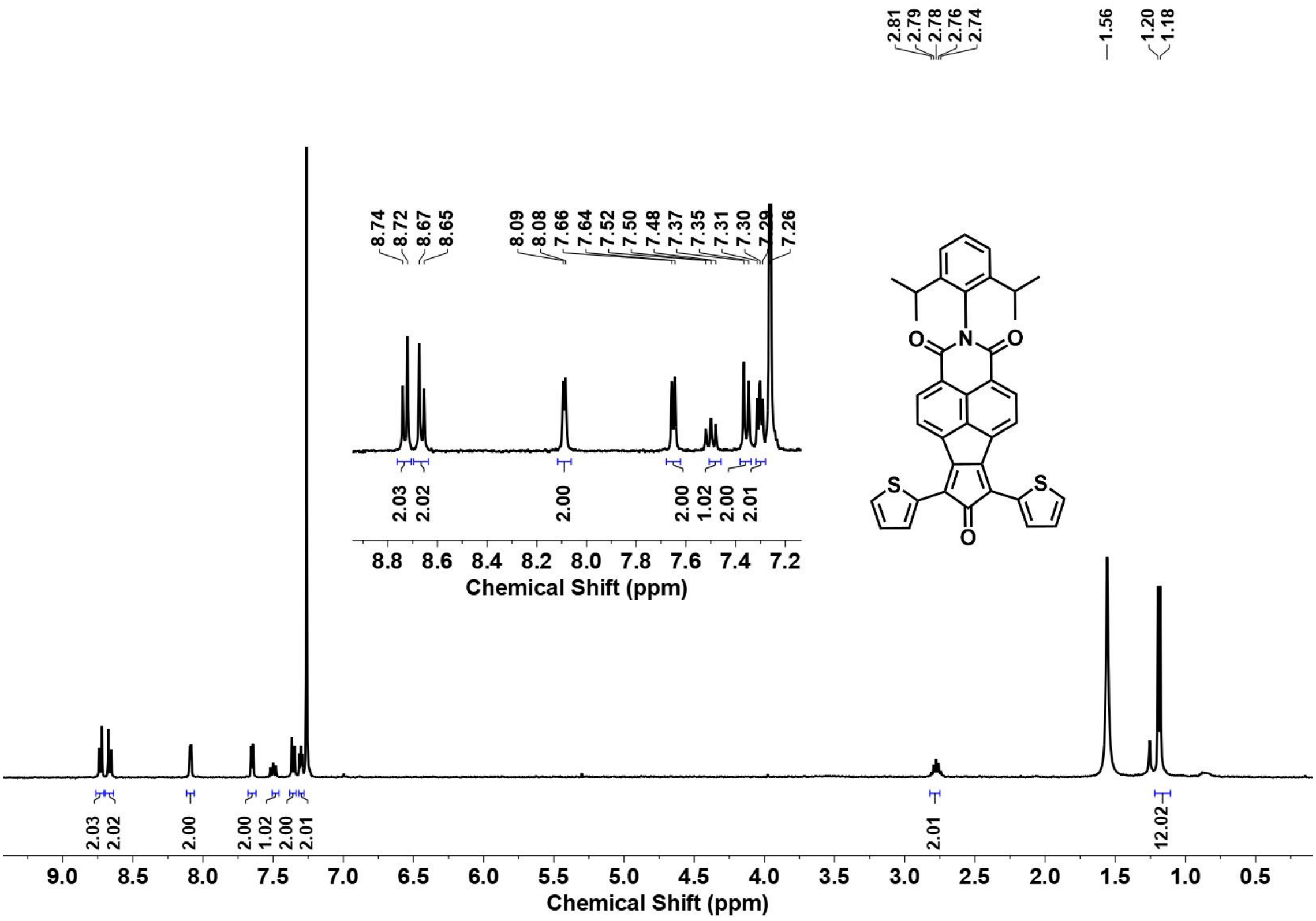

Figure S19. ^{1}H NMR spectra of compound **6a** in $CDCl_3$ (298 K).

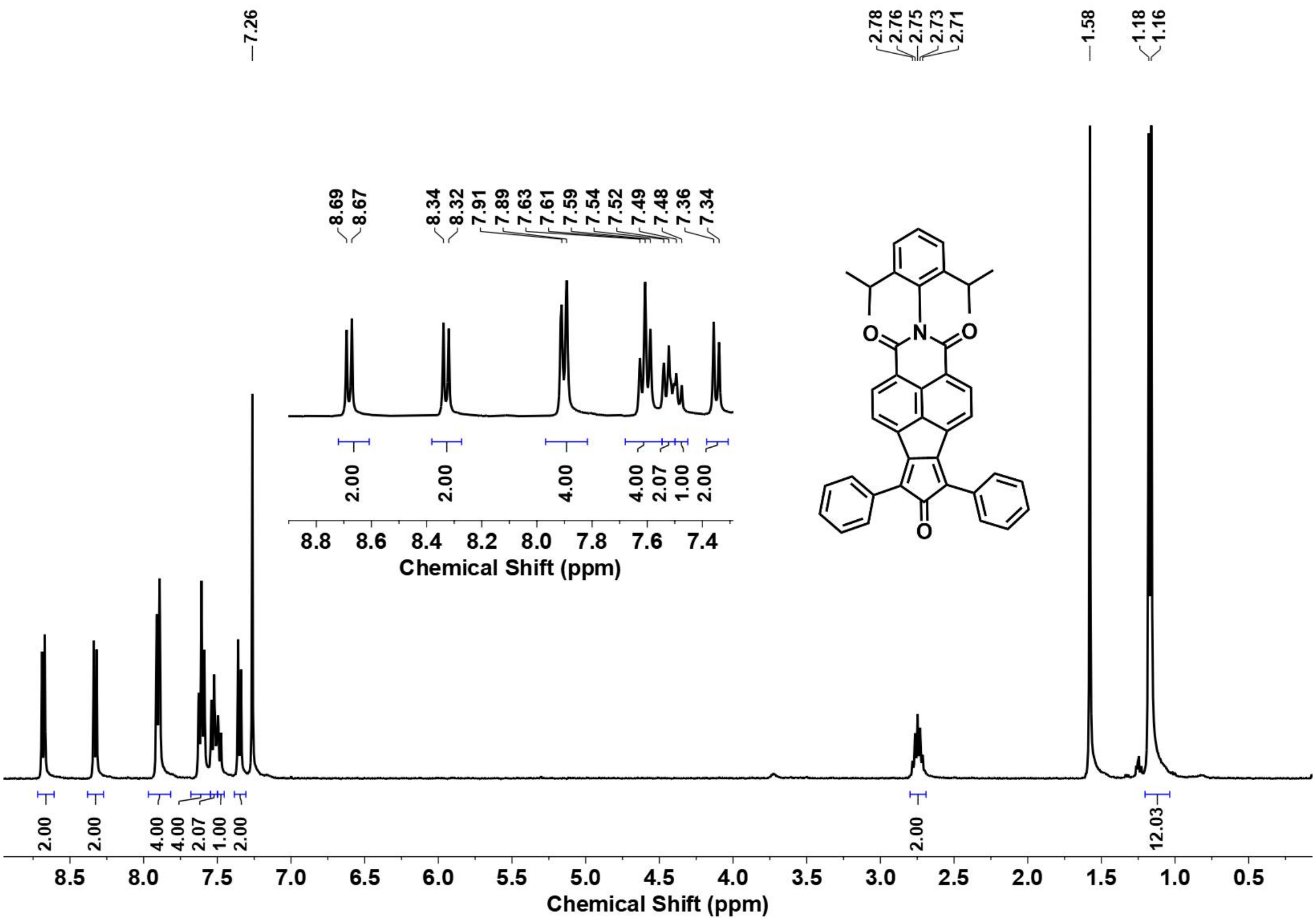

Figure S20. ^{1}H NMR spectra of compound **6b** in $CDCl_3$ (298 K).

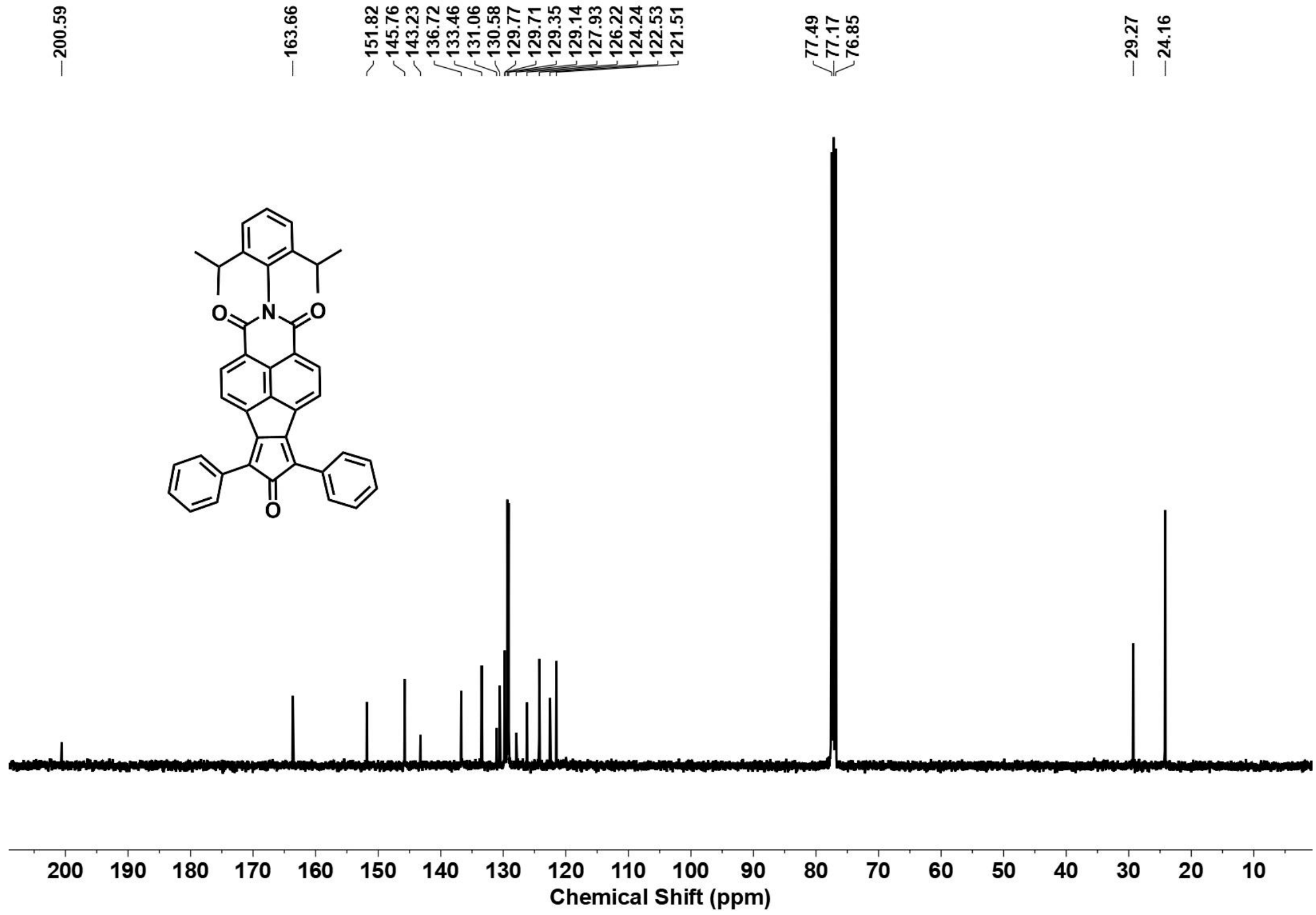

Figure S21. ^{13}C NMR spectra of compound **6b** in $CDCl_3$ (298 K).

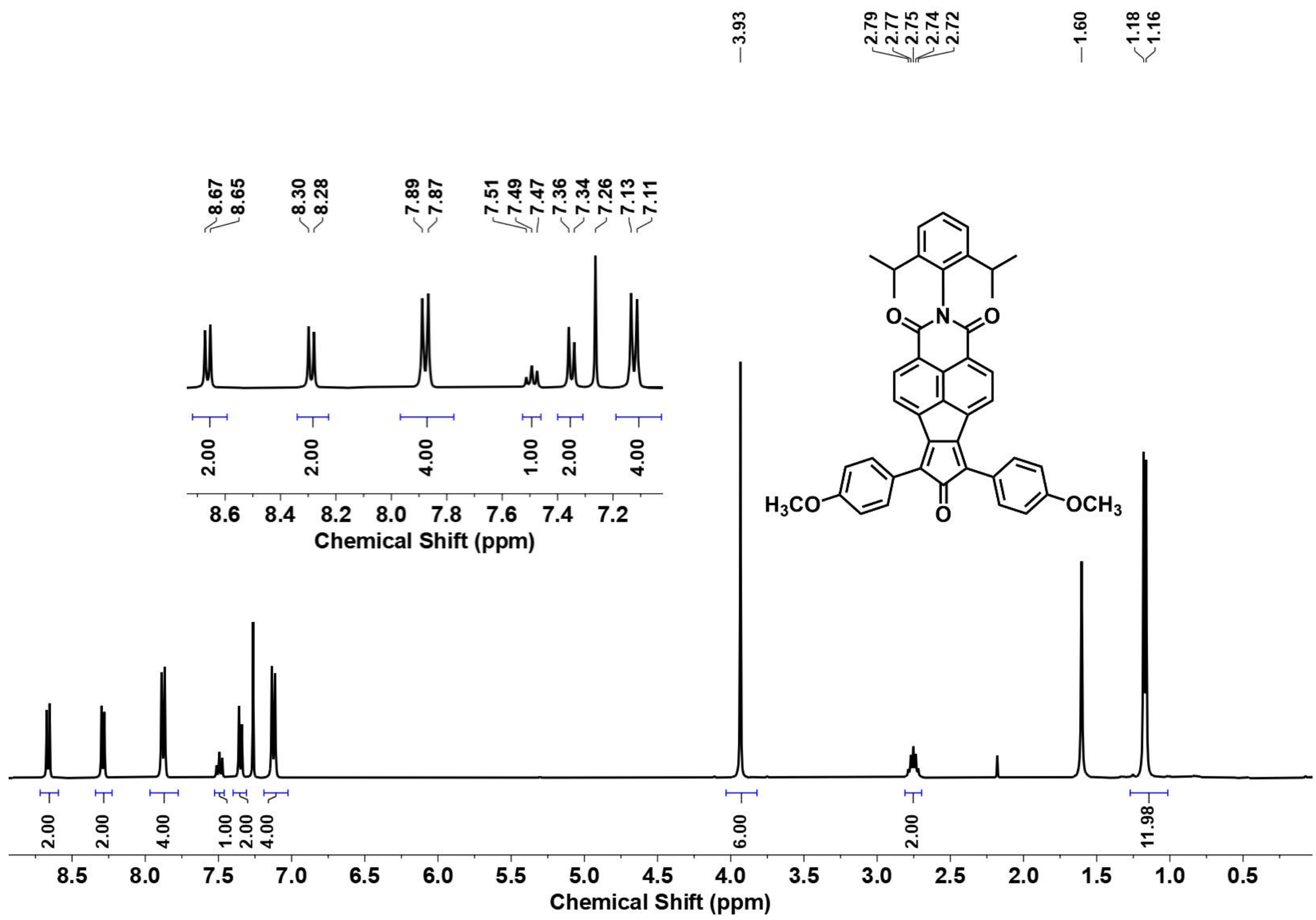

Figure S22. ^{1}H NMR spectra of compound **6c** in $CDCl_3$ (298 K).

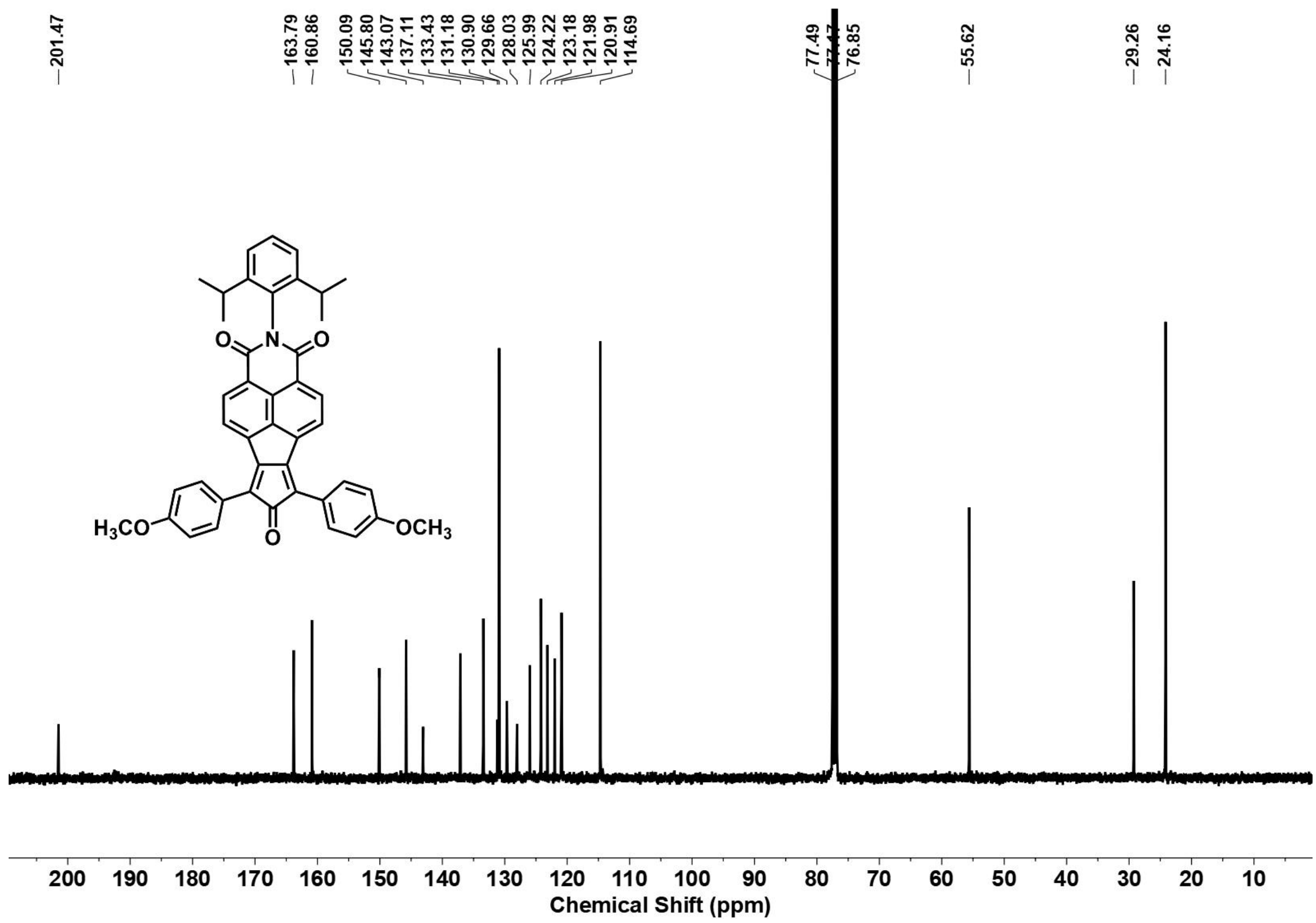

Figure S23. ^{13}C NMR spectra of compound **6c** in $CDCl_3$ (298 K).

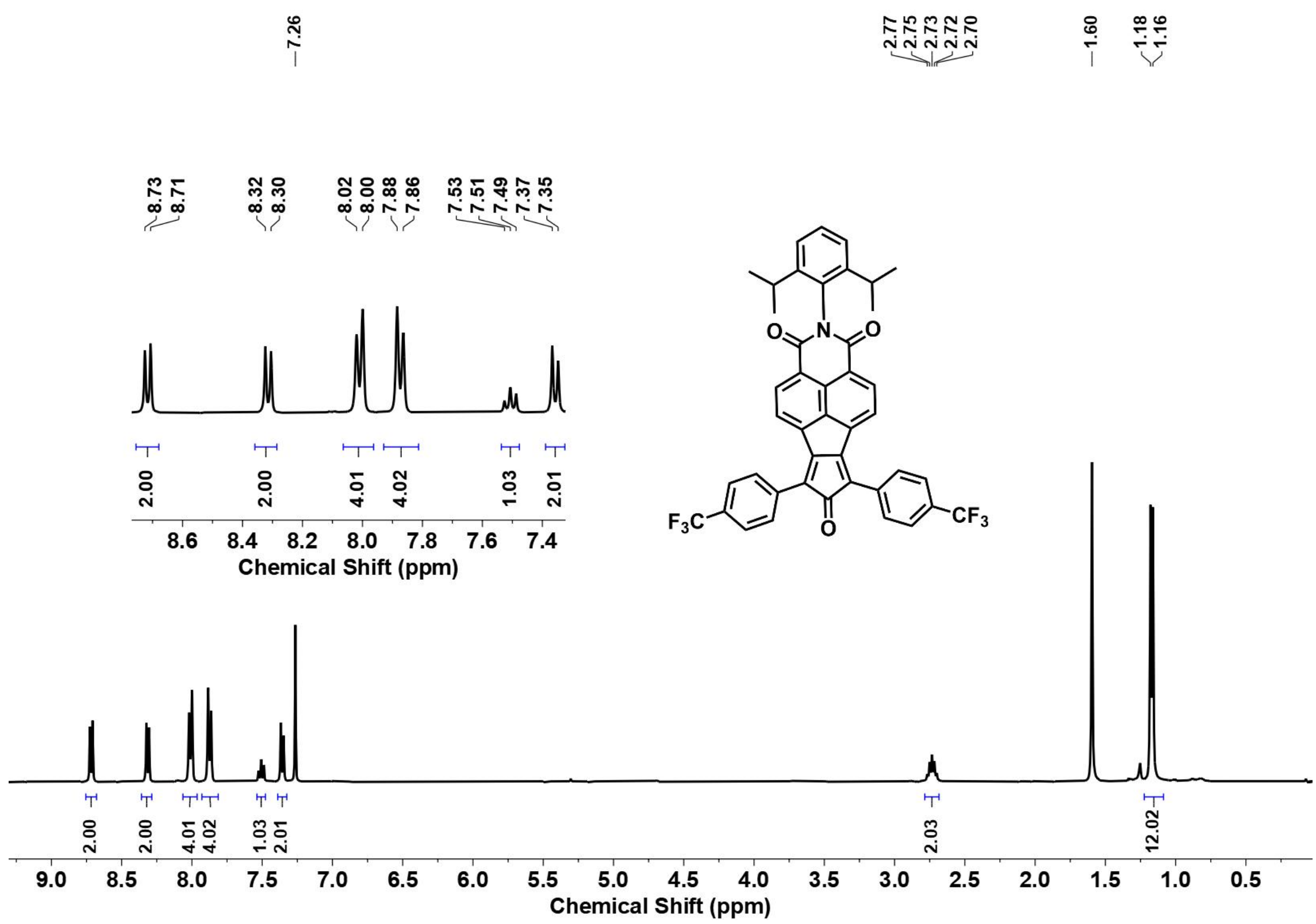

Figure S24. ^{1}H NMR spectra of compound **6d** in $CDCl_3$ (298 K).

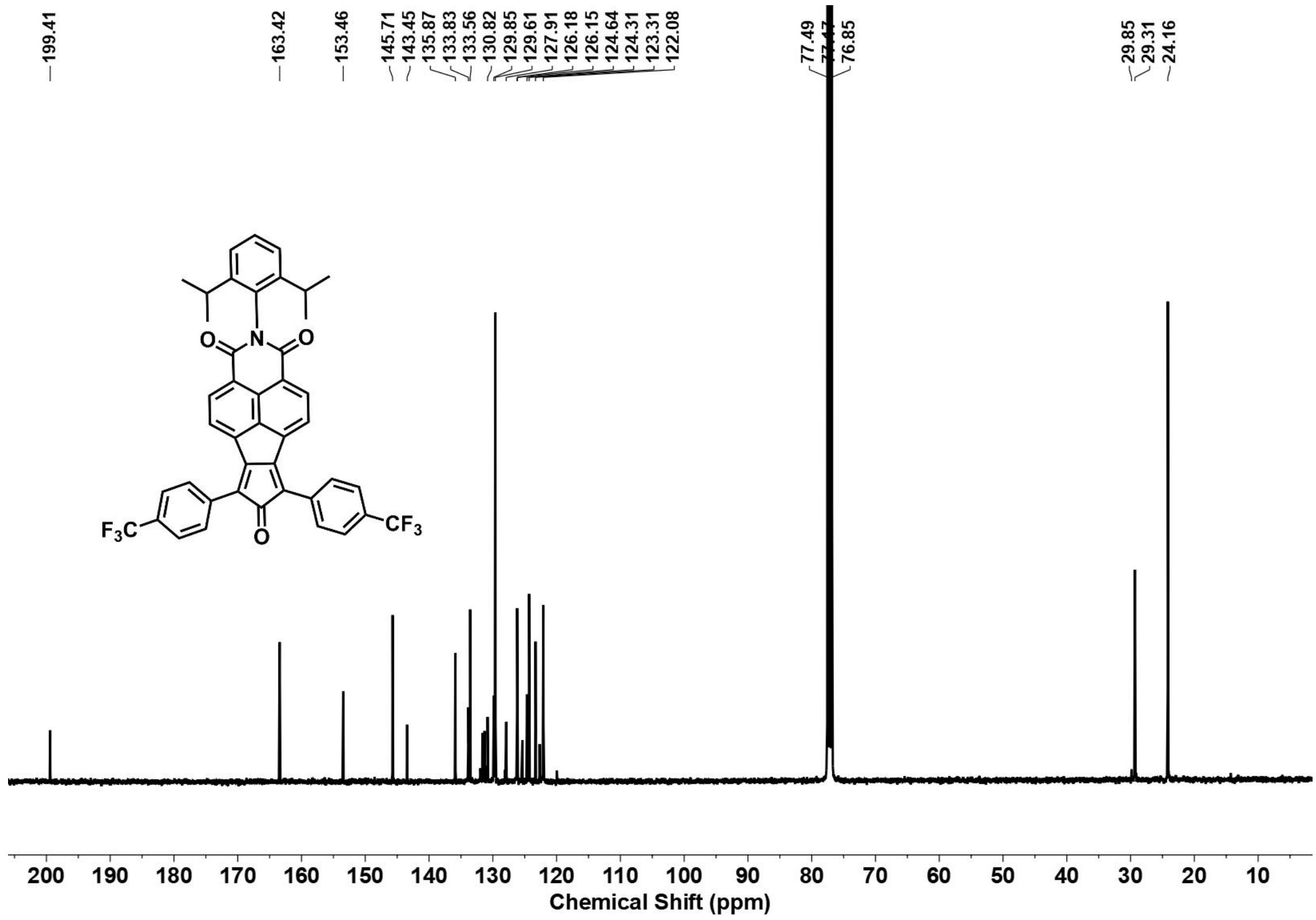

Figure S25. ^{13}C NMR spectra of compound **6d** in $CDCl_3$ (298 K).

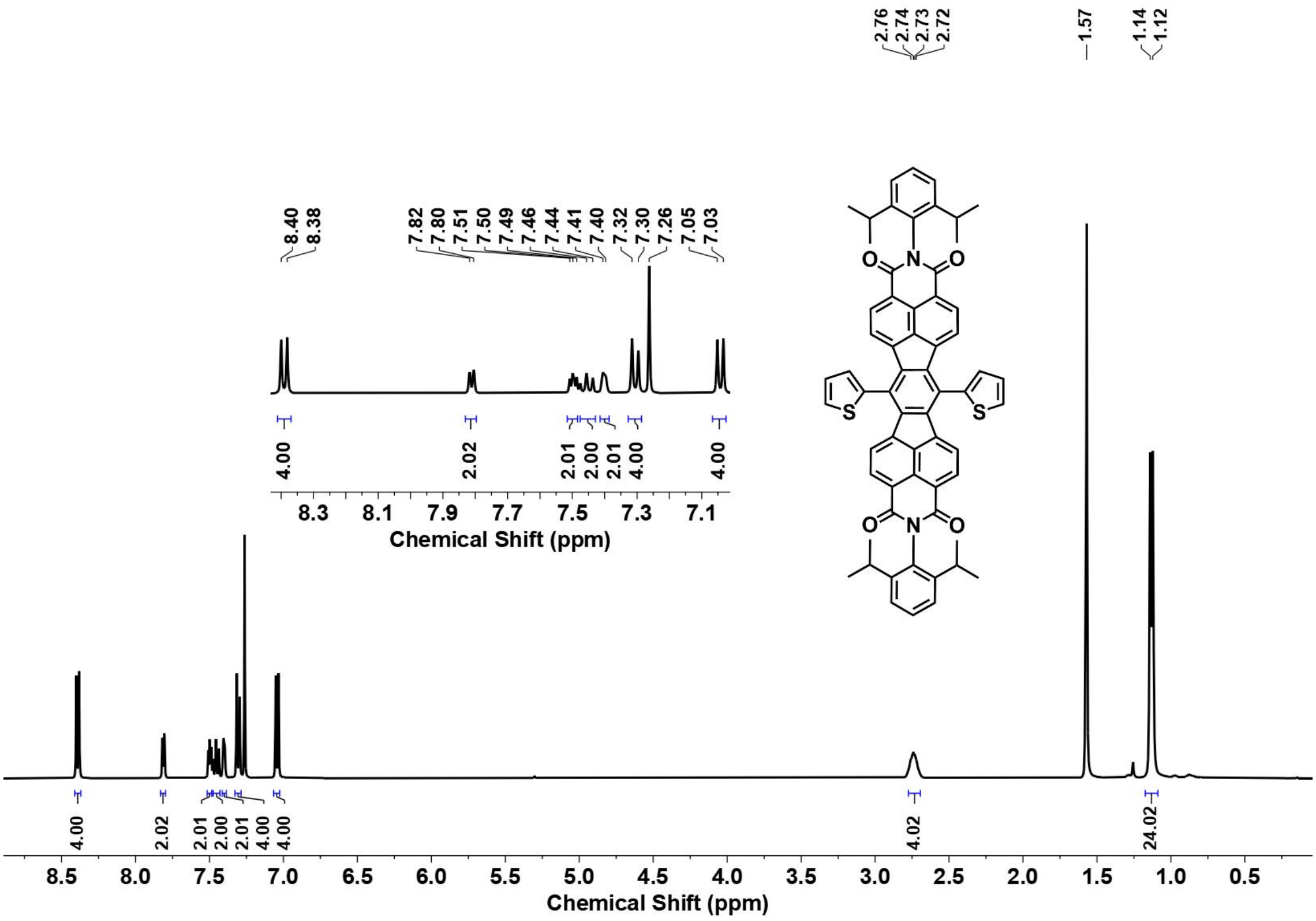

Figure S26. ^{1}H NMR spectra of **L1** in $CDCl_3$ (298 K).

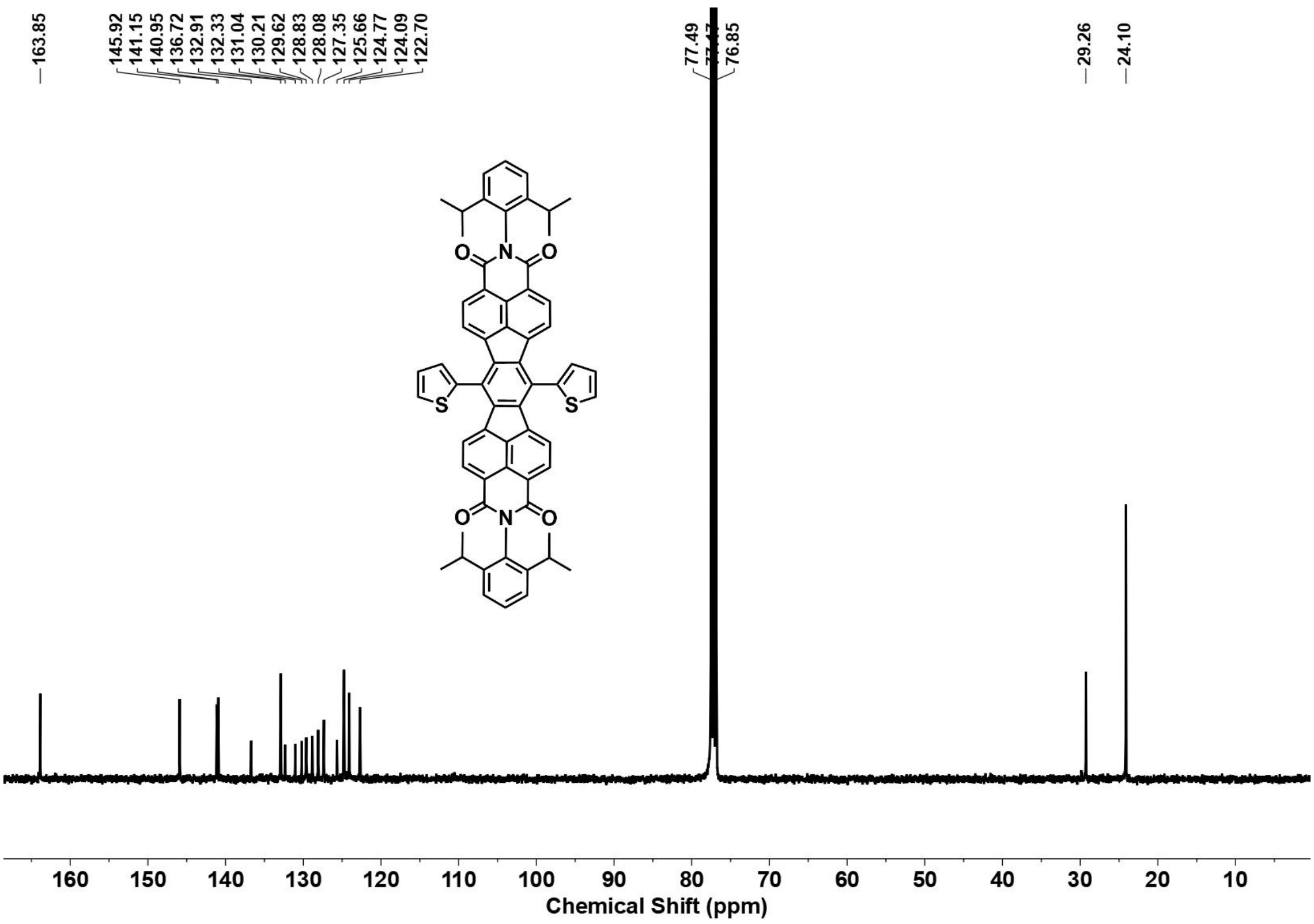

Figure S27. ^{13}C NMR spectra of **L1** in $CDCl_3$ (298 K).

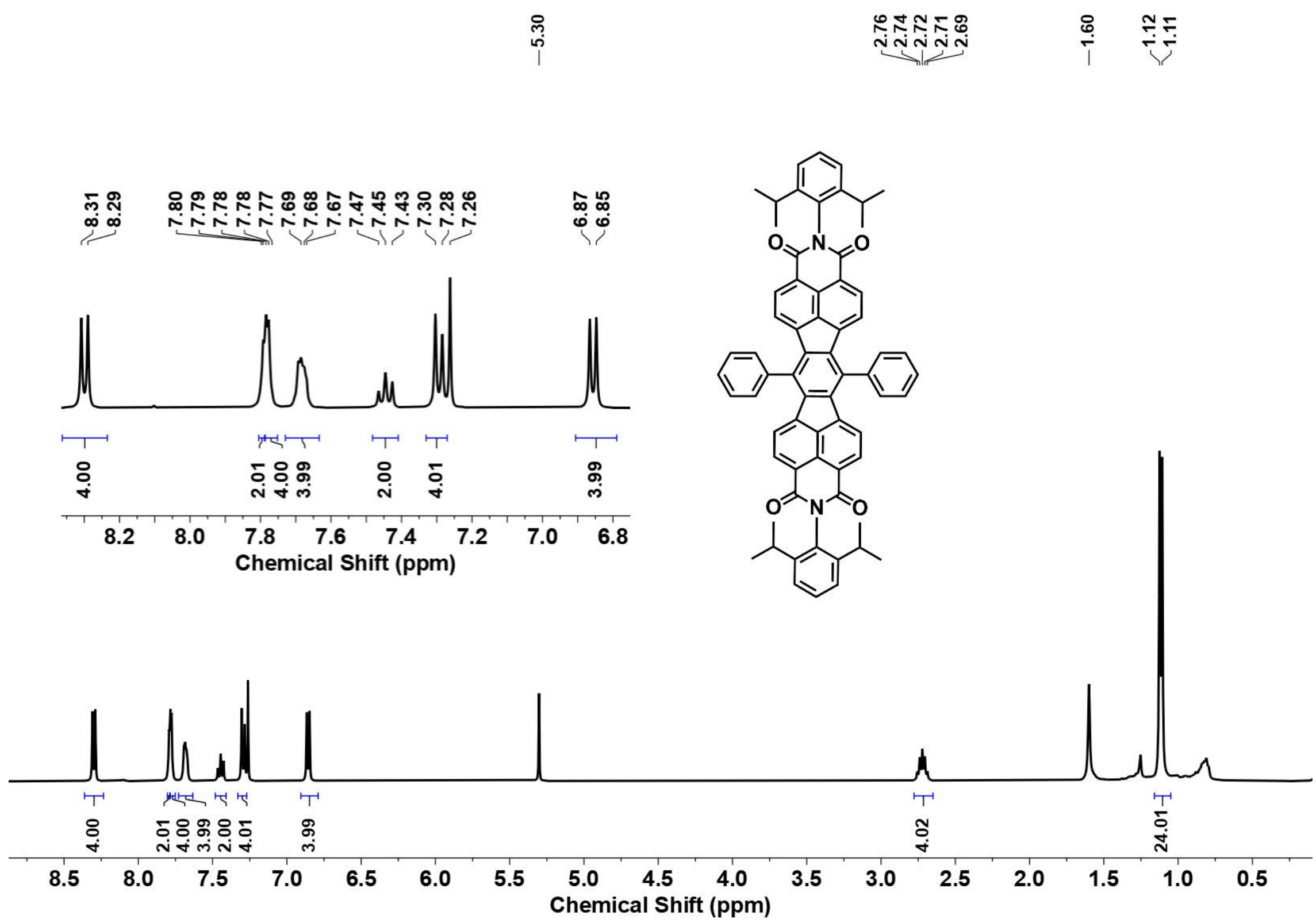

Figure S28. ^{1}H NMR spectra of **L2** in $CDCl_3$ (298 K).

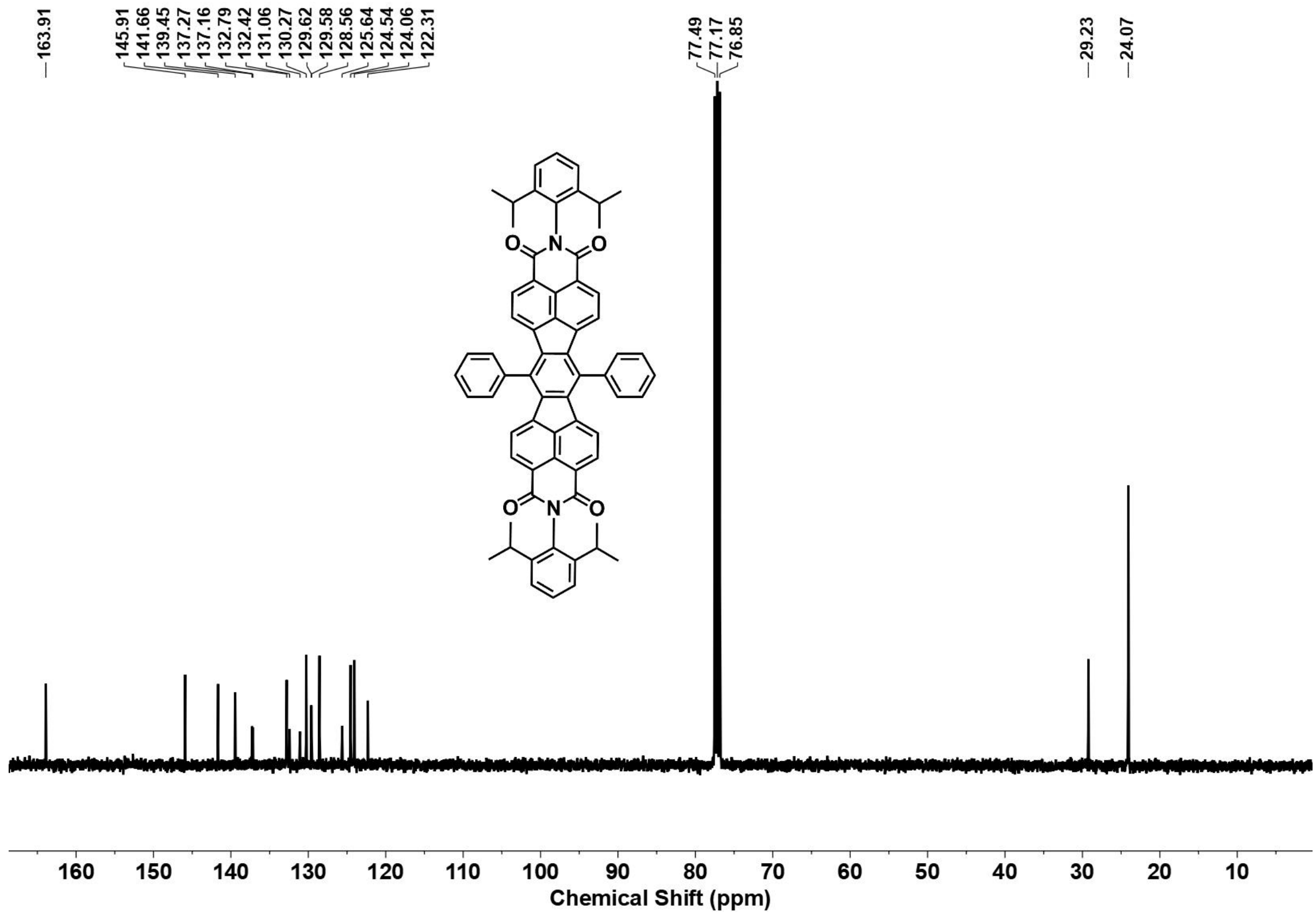

Figure S29. ^{13}C NMR spectra of **L2** in $CDCl_3$ (298 K).

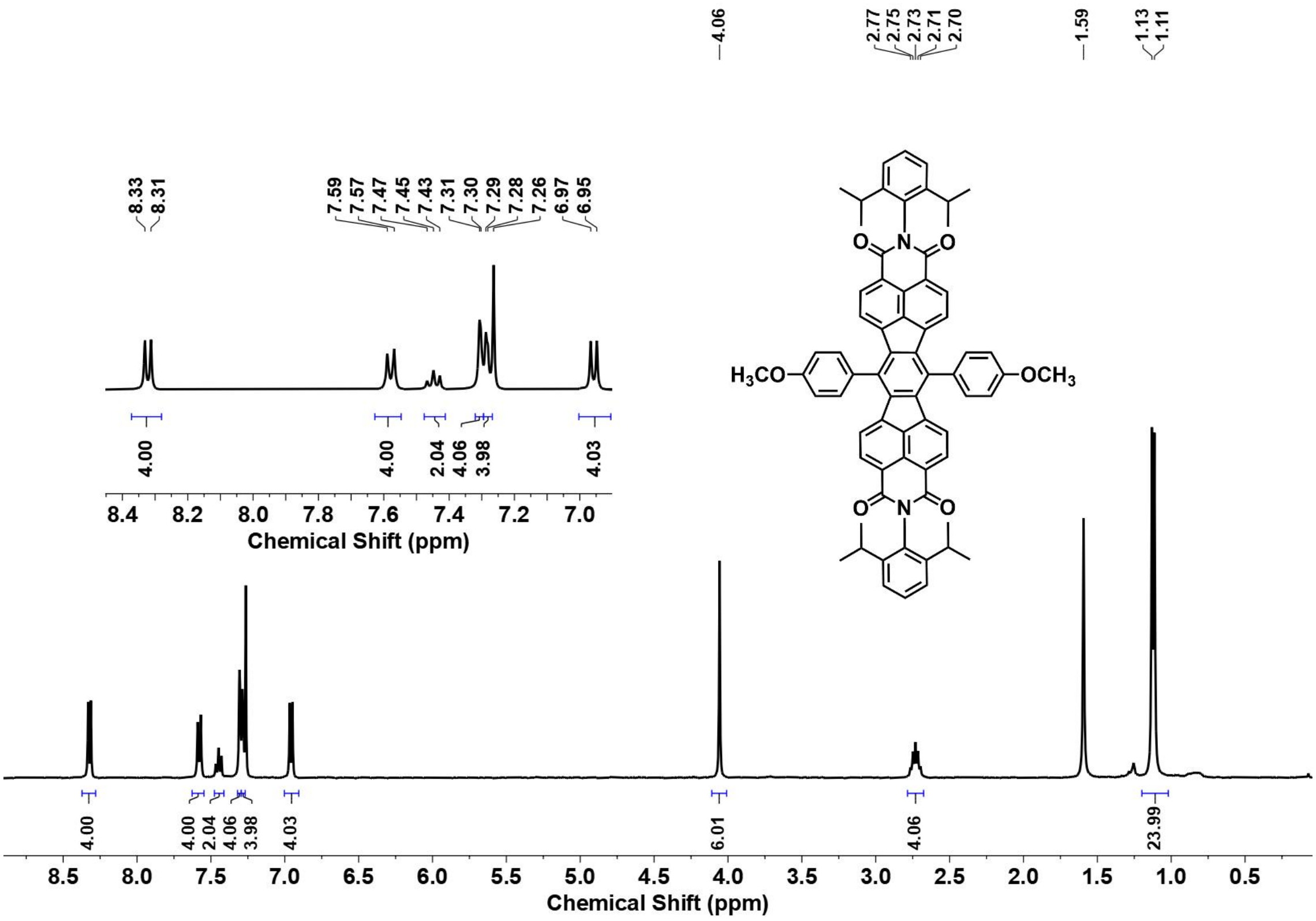

Figure S30. ^{1}H NMR spectra of **L3** in $CDCl_3$ (298 K).

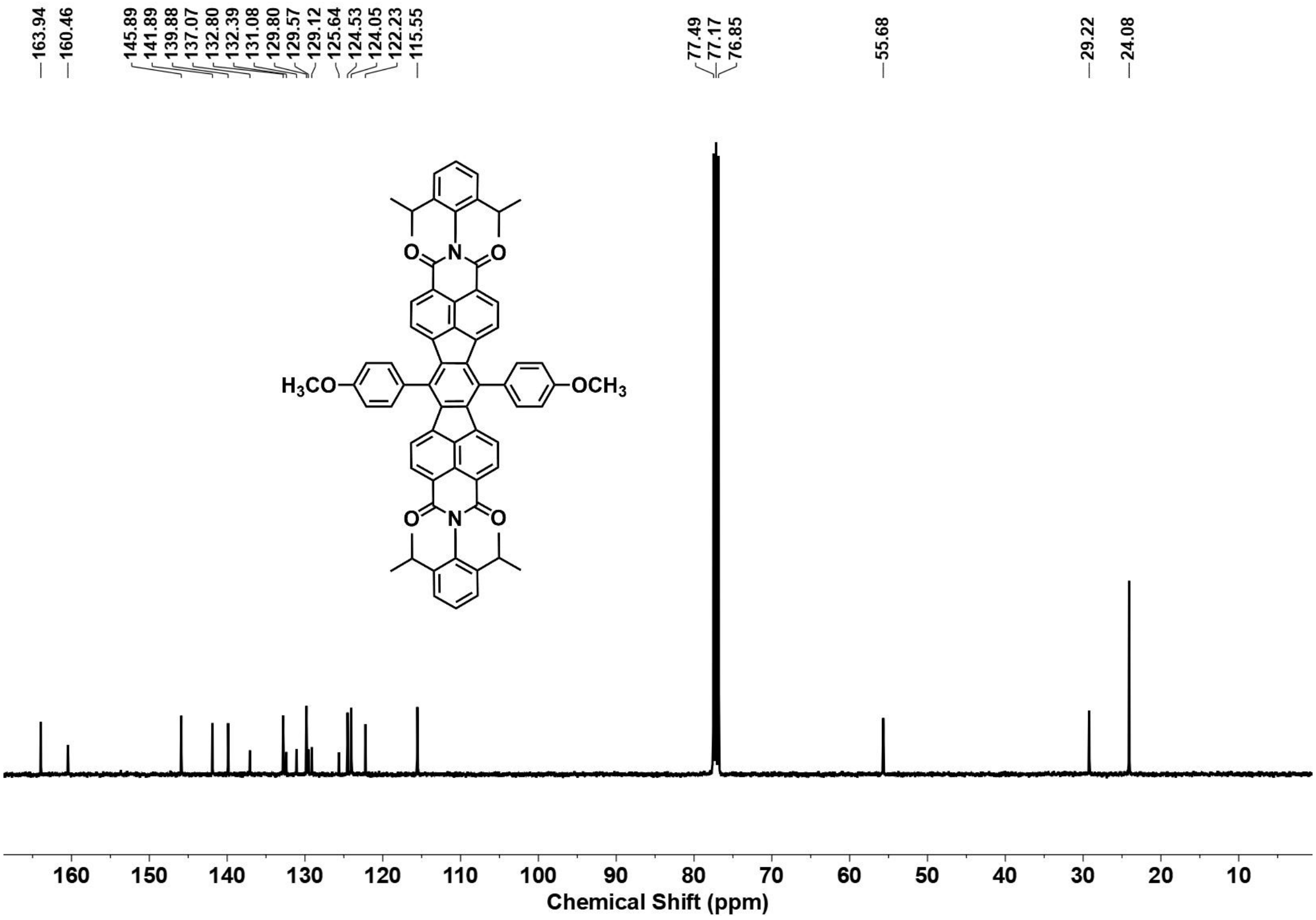

Figure S31. ^{13}C NMR spectra of **L3** in $CDCl_3$ (298 K).

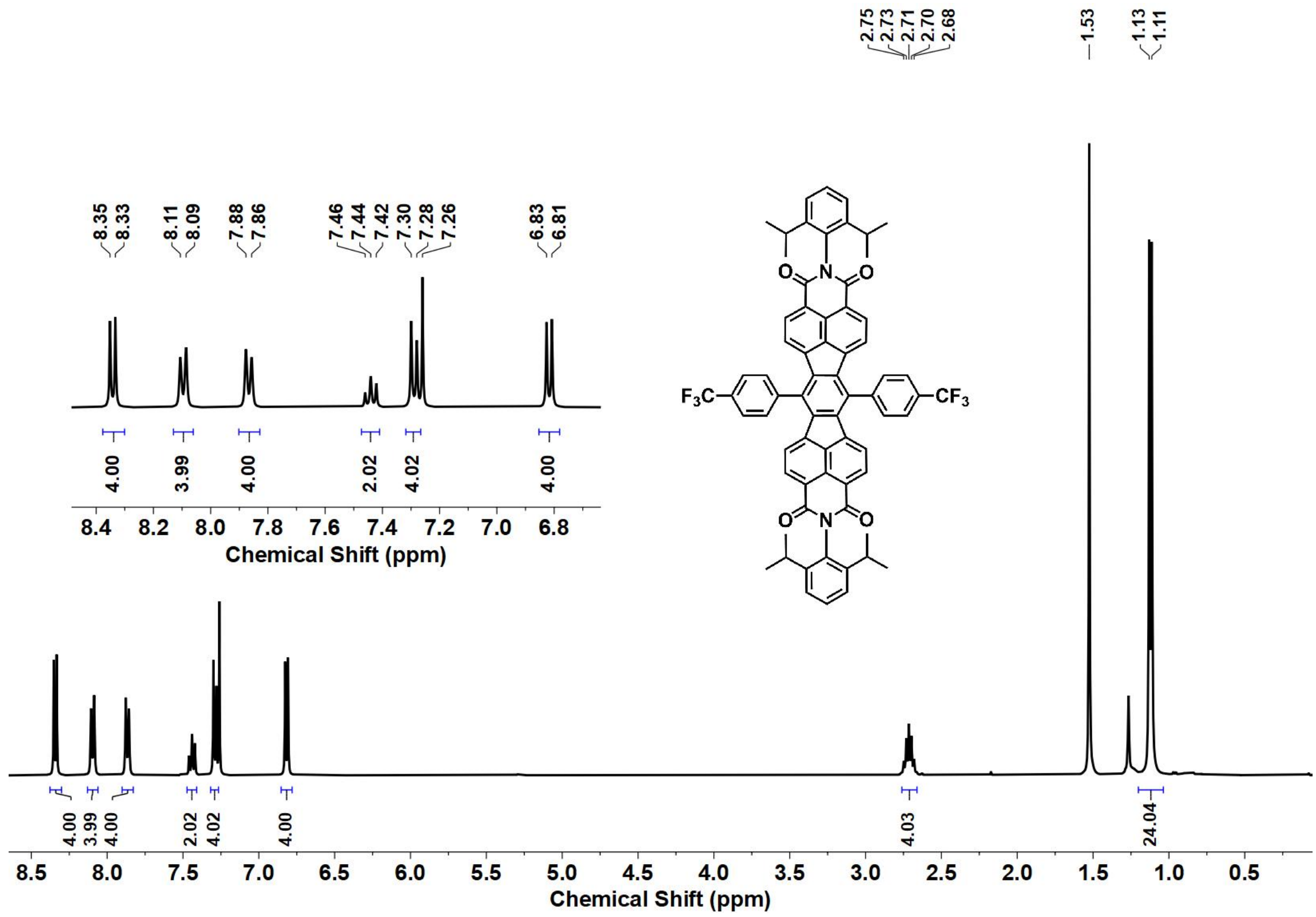

Figure S32. ^{1}H NMR spectra of **L4** in $CDCl_3$ (313 K).

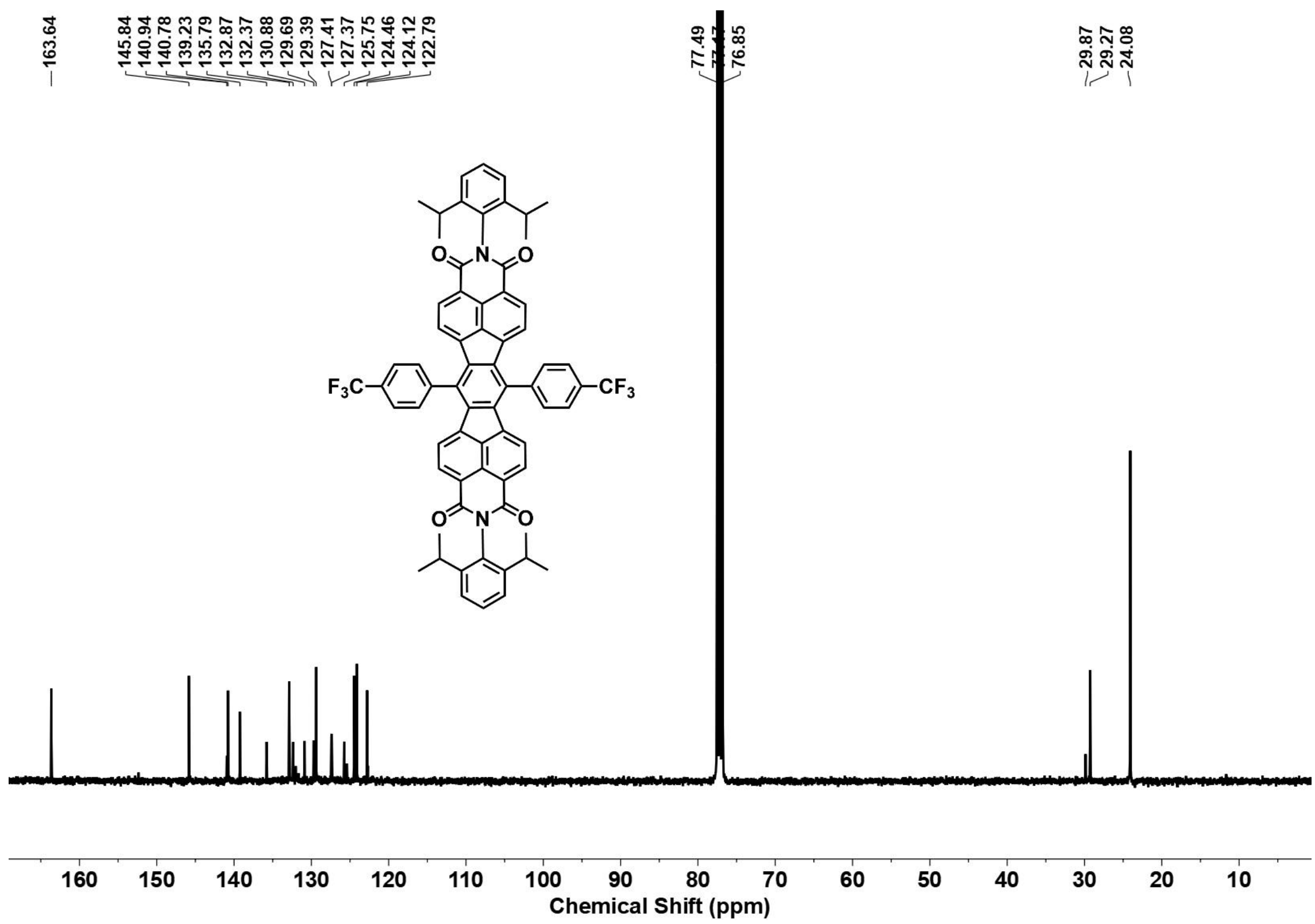

Figure S33. ^{13}C NMR spectra of **L4** in $CDCl_3$ (298 K).

9. Computational Atomic Coordinate

The model structure of **L1**

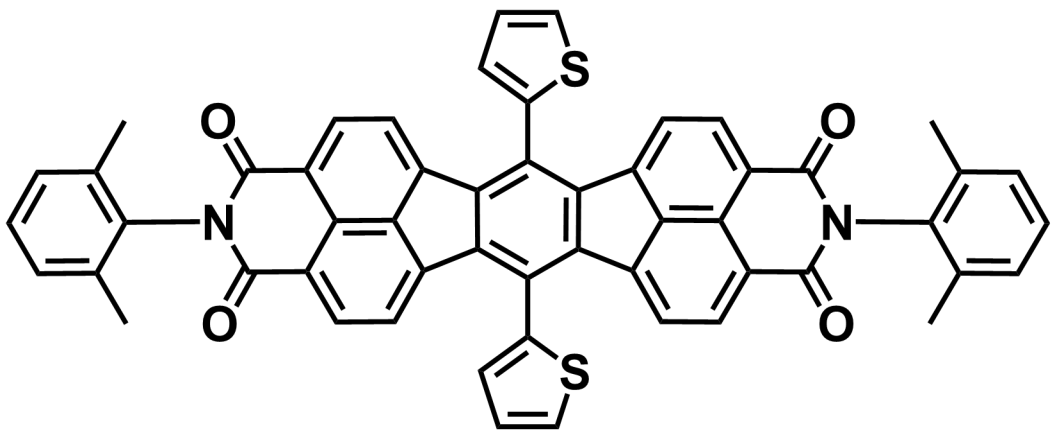

94 atoms
Total energy: -3284.215193 (Hartree)

Table S8. Computational atomic coordinates of ground-state **L1** structure calculated at the DFT//B3LYP-D3(BJ)/6-31G(d) level.

#	Atom	Coordinate		
		X	Y	Z
1	C	11.069671	0.062589	-1.240392
2	C	9.672303	0.063514	-1.271098
3	C	8.991953	-0.000106	-0.050952
4	C	9.652672	-0.06376	1.179953
5	C	11.050379	-0.062831	1.171516
6	C	11.754732	-0.000112	-0.028873
7	N	7.54459	-0.00007	-0.062332
8	C	6.911541	1.265865	-0.069271
9	C	5.432429	1.249623	-0.078201
10	C	4.771881	-0.00005	-0.080761
11	C	5.432398	-1.249737	-0.076901
12	C	6.911493	-1.265997	-0.066165
13	C	4.67751	2.416922	-0.086491
14	C	3.256963	2.399619	-0.097019
15	C	2.598121	1.178062	-0.096538
16	C	3.383134	-0.000036	-0.089279
17	C	3.256912	-2.399695	-0.095643
18	C	4.677456	-2.417025	-0.084476
19	C	2.598101	-1.178127	-0.096101
20	C	1.195777	-0.716519	-0.103223
21	C	1.195795	0.716487	-0.103319
22	C	-0.000003	1.445064	-0.102047
23	C	-0.000037	-1.445066	-0.102134
24	C	8.875116	-0.135042	2.468418
25	C	8.915366	0.134792	-2.571778
26	O	7.571454	-2.29236	-0.059492
27	O	7.571551	2.29222	-0.069579
28	C	0.000035	2.921539	-0.082726

29	C	0.000001	-2.921545	-0.082843
30	C	-11.06958	-0.062372	-1.240722
31	C	-9.672209	-0.063296	-1.271285
32	C	-8.991983	0.00011	-0.051058
33	C	-9.652826	0.063554	1.179789
34	C	-11.050533	0.062629	1.171211
35	C	-11.754764	0.00012	-0.029261
36	N	-7.54462	0.000072	-0.062286
37	C	-6.911524	1.266	-0.066037
38	C	-5.432427	1.249738	-0.076668
39	C	-4.771917	0.000048	-0.080448
40	C	-5.432462	-1.249623	-0.078001
41	C	-6.911571	-1.265864	-0.069207
42	C	-4.677483	2.417026	-0.084302
43	C	-3.256939	2.399691	-0.095414
44	C	-2.598133	1.178118	-0.095712
45	C	-3.383166	0.000034	-0.088883
46	C	-3.256999	-2.39962	-0.096896
47	C	-4.677543	-2.416926	-0.086396
48	C	-2.598161	-1.178061	-0.096264
49	C	-1.195827	-0.716488	-0.103092
50	C	-1.195815	0.716518	-0.102936
51	C	-8.875397	0.134608	2.468343
52	C	-8.915137	-0.134337	-2.5719
53	O	-7.571585	-2.292217	-0.069649
54	O	-7.571487	2.292362	-0.059443
55	C	0.001477	-3.788743	-1.144589
56	C	0.001319	-5.162642	-0.74996
57	C	-0.000225	-5.321433	0.608703
58	S	-0.001525	-3.794294	1.432542
59	C	-0.000326	3.78874	-1.144472
60	C	-0.000107	5.162636	-0.749839
61	C	0.000416	5.321424	0.608826
62	S	0.000658	3.79428	1.432659
63	H	11.619828	0.111569	-2.176166
64	H	11.585493	-0.111811	2.115963
65	H	12.84112	-0.000106	-0.020179
66	H	5.20529	3.364987	-0.085661
67	H	2.714235	3.337819	-0.110197
68	H	2.71417	-3.337891	-0.108436
69	H	5.20521	-3.365102	-0.082433
70	H	8.260765	-1.042075	2.508007
71	H	9.549943	-0.147012	3.328869

72	H	8.204422	0.72521	2.576646
73	H	9.603808	0.146579	-3.421396
74	H	8.246407	-0.72545	-2.690456
75	H	8.301904	1.041961	-2.621063
76	H	-11.619643	-0.111188	-2.17656
77	H	-11.585743	0.111448	2.115612
78	H	-12.841152	0.000115	-0.020678
79	H	-5.205243	3.3651	-0.082384
80	H	-2.714189	3.337882	-0.108256
81	H	-2.714264	-3.337814	-0.110205
82	H	-5.20533	-3.364987	-0.085691
83	H	-8.260967	1.041579	2.508109
84	H	-9.55031	0.14653	3.328727
85	H	-8.204793	-0.72572	2.57653
86	H	-9.603495	-0.146078	-3.421587
87	H	-8.246251	0.725986	-2.690399
88	H	-8.301582	-1.04144	-2.621247
89	H	0.002626	-3.449411	-2.17428
90	H	0.002268	-5.991603	-1.448652
91	H	-0.000678	-6.242449	1.175649
92	H	-0.000762	3.449408	-2.174163
93	H	-0.000338	5.991603	-1.448527
94	H	0.000729	6.242434	1.175781

The model structure of **L2**

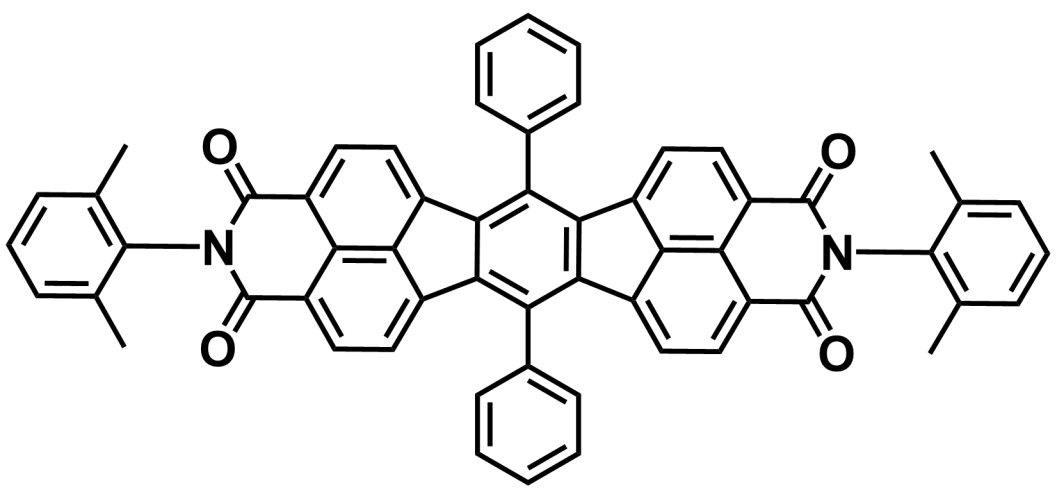

100 atoms

Total energy: -2642.713430 (Hartree)

Table S9. Computational atomic coordinates of ground-state **L2** structure calculated at the DFT//B3LYP-D3(BJ)/6-31G(d) level.

#	Atom	Coordinate		
		X	Y	Z
1	C	11.055269	-0.067004	1.221562
2	C	9.657622	-0.068243	1.235791
3	C	8.991487	0.000475	0.008062
4	C	9.666897	0.069466	-1.214569

5	C	11.064396	0.068712	-1.189771
6	C	11.754614	0.00097	0.018531
7	N	7.544246	0.000249	0.002512
8	C	6.911159	-1.26587	0.002681
9	C	5.432524	-1.249572	-0.00425
10	C	4.771345	-0.00013	-0.010755
11	C	5.432185	1.249506	-0.006461
12	C	6.910823	1.266198	-0.001517
13	C	4.677414	-2.41676	-0.001213
14	C	3.256985	-2.399668	-0.005498
15	C	2.596906	-1.178389	-0.014601
16	C	3.382448	-0.000326	-0.016552
17	C	3.256371	2.399052	-0.008414
18	C	4.676795	2.416524	-0.004996
19	C	2.596684	1.177558	-0.015586
20	C	1.193335	0.716332	-0.014587
21	C	1.193392	-0.717431	-0.014478
22	C	-0.000006	-1.447705	-0.000132
23	C	-0.000004	1.446578	0.00013
24	C	8.904554	0.146593	-2.511748
25	C	8.885514	-0.145511	2.527164
26	O	7.571275	2.292498	-0.00245
27	O	7.571847	-2.291992	0.010008
28	C	-0.000006	-2.936328	-0.000275
29	C	0	2.935234	0.000262
30	C	-11.055259	-0.065508	-1.221624
31	C	-9.657612	-0.066723	-1.235857
32	C	-8.991484	0.00049	-0.008047
33	C	-9.666877	0.067964	1.21467
34	C	-11.064378	0.067219	1.189882
35	C	-11.754598	0.000968	-0.018504
36	N	-7.544242	0.000263	-0.002509
37	C	-6.911171	-1.26586	-0.002939
38	C	-5.432536	-1.249568	0.003974
39	C	-4.771357	-0.000126	0.010661
40	C	-5.432195	1.249515	0.006653
41	C	-6.910833	1.266208	0.001773
42	C	-4.677427	-2.416756	0.000789
43	C	-3.256998	-2.399666	0.005086
44	C	-2.596917	-1.178388	0.014333
45	C	-3.382459	-0.000325	0.016431
46	C	-3.256378	2.399055	0.008885
47	C	-4.676801	2.416531	0.005469

48	C	-2.596693	1.177558	0.015746
49	C	-1.193345	0.716331	0.014727
50	C	-1.193403	-0.717431	0.014356
51	C	-8.904498	0.143423	2.511923
52	C	-8.885479	-0.142352	-2.527311
53	O	-7.571296	2.292501	0.002965
54	O	-7.571881	-2.291966	-0.010391
55	C	-0.104135	3.642737	1.204238
56	C	-0.103794	5.037619	1.204104
57	C	0.000014	5.737498	0.000514
58	C	0.103815	5.037835	-1.203202
59	C	0.104142	3.642952	-1.203586
60	C	0.116869	-3.643861	-1.203077
61	C	0.116503	-5.038738	-1.202962
62	C	-0.000008	-5.738627	-0.000543
63	C	-0.116517	-5.038968	1.202011
64	C	-0.116882	-3.644091	1.202392
65	H	11.594336	-0.119923	2.163567
66	H	11.610573	0.121843	-2.127661
67	H	12.84104	0.001159	0.022654
68	H	5.205238	-3.364835	0.005706
69	H	2.715421	-3.338492	-0.001764
70	H	2.714492	3.33771	-0.005658
71	H	5.204398	3.364734	-0.000074
72	H	8.293277	1.055516	-2.555366
73	H	9.589343	0.159243	-3.364321
74	H	8.232696	-0.711405	-2.630132
75	H	9.563839	-0.157969	3.38489
76	H	8.212607	0.712352	2.640426
77	H	8.274143	-1.05457	2.566193
78	H	-11.594329	-0.11726	-2.163692
79	H	-11.610555	0.11917	2.127838
80	H	-12.841024	0.001163	-0.022621
81	H	-5.205252	-3.36483	-0.00625
82	H	-2.715436	-3.338491	0.001265
83	H	-2.714496	3.337712	0.006382
84	H	-5.204403	3.364743	0.000789
85	H	-8.292708	1.051957	2.556456
86	H	-9.589283	0.155606	3.364507
87	H	-8.233123	-0.715074	2.629451
88	H	-9.56381	-0.15437	-3.385039
89	H	-8.213068	0.716009	-2.639751
90	H	-8.273587	-1.051022	-2.567229

91	H	-0.18536	3.093672	2.137719
92	H	-0.183878	5.577298	2.143296
93	H	0.000019	6.82357	0.000612
94	H	0.183905	5.57768	-2.142296
95	H	0.185363	3.094055	-2.137165
96	H	0.208047	-3.094811	-2.135649
97	H	0.206441	-5.578402	-2.141269
98	H	-0.000009	-6.8247	-0.000647
99	H	-0.206455	-5.578812	2.140214
100	H	-0.208058	-3.095219	2.135069

The model structure of **L3**

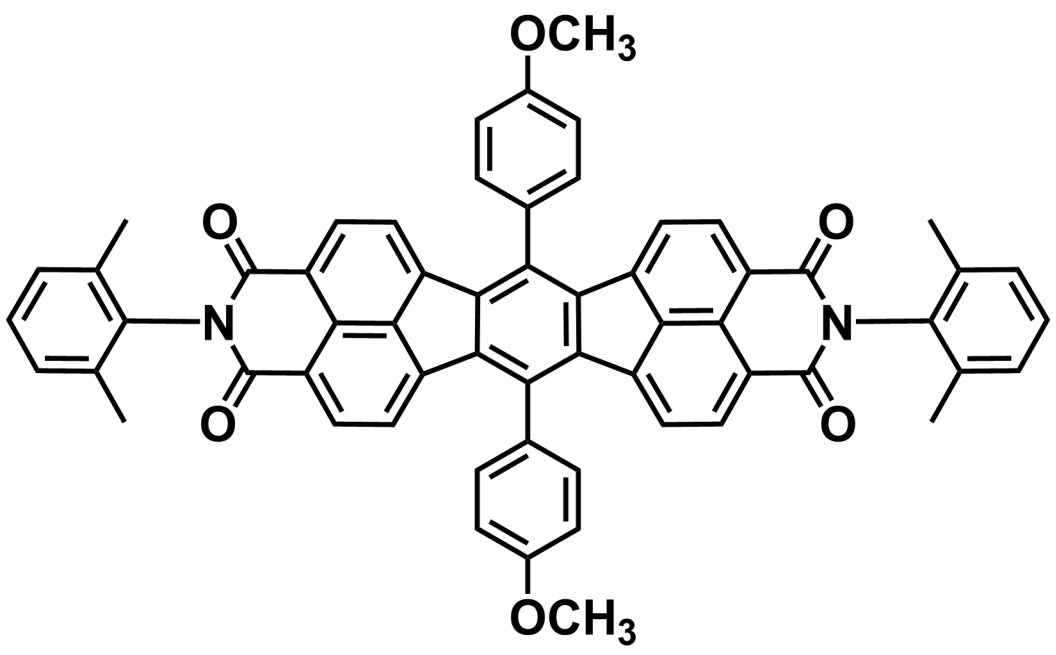

108 atoms

Total energy: -2871.773556 (Hartree)

Table S10. Computational atomic coordinates of ground-state **L3** structure calculated at the DFT//B3LYP-D3(BJ)/6-31G(d) level.

#	Atom	Coordinate		
		X	Y	Z
1	C	11.058967	-0.080734	-1.169319
2	C	9.661373	-0.078504	-1.187395
3	C	8.991699	-0.019779	0.03897
4	C	9.663997	0.036022	1.264046
5	C	11.061577	0.032164	1.243125
6	C	11.755113	-0.025798	0.036231
7	N	7.54459	-0.016606	0.040469
8	C	6.914012	1.250643	0.065827
9	C	5.435699	1.237218	0.066593
10	C	4.771677	-0.01054	0.043573
11	C	5.430095	-1.261286	0.019729
12	C	6.908343	-1.281161	0.015931
13	C	4.682834	2.405652	0.090492
14	C	3.262529	2.391671	0.087106
15	C	2.59935	1.172144	0.060582

16	C	3.382678	-0.007427	0.044795
17	C	3.251724	-2.406077	0.0058
18	C	4.672005	-2.4264	-0.000407
19	C	2.594103	-1.183546	0.03111
20	C	1.191407	-0.719453	0.04372
21	C	1.194533	0.71425	0.049529
22	C	0.003172	1.449058	0.045773
23	C	-0.003155	-1.449045	0.045792
24	C	8.898393	0.103319	2.559829
25	C	8.892851	-0.142556	-2.481623
26	O	7.567183	-2.30836	-0.008915
27	O	7.577317	2.2751	0.086943
28	C	0.006501	2.936486	0.044011
29	C	-0.006499	-2.936475	0.044008
30	C	-11.058919	0.080717	-1.169397
31	C	-9.661325	0.078494	-1.187436
32	C	-8.991682	0.019767	0.038946
33	C	-9.664012	-0.036043	1.264004
34	C	-11.061591	-0.032192	1.243048
35	C	-11.755097	0.025772	0.036136
36	N	-7.544574	0.016598	0.040478
37	C	-6.908331	1.281156	0.015961
38	C	-5.430084	1.261286	0.019761
39	C	-4.771661	0.010544	0.043662
40	C	-5.435678	-1.237218	0.066671
41	C	-6.913992	-1.250648	0.065854
42	C	-4.671997	2.4264	-0.000448
43	C	-3.251716	2.406083	0.005754
44	C	-2.594089	1.183556	0.031156
45	C	-3.382662	0.007436	0.044903
46	C	-3.262505	-2.391665	0.087142
47	C	-4.682811	-2.40565	0.090546
48	C	-2.599333	-1.172135	0.060655
49	C	-1.194515	-0.714238	0.049543
50	C	-1.191391	0.719466	0.043725
51	C	-8.898441	-0.103344	2.559807
52	C	-8.89277	0.142551	-2.481645
53	O	-7.577294	-2.275106	0.087036
54	O	-7.567176	2.30835	-0.008953
55	C	0.203129	-3.655357	1.231284
56	C	0.199436	-5.043185	1.233799
57	C	-0.015141	-5.749182	0.04024
58	C	-0.225068	-5.045799	-1.151887

59	C	-0.218725	-3.649304	-1.137824
60	C	-0.203376	3.655351	1.231246
61	C	-0.199732	5.043182	1.233772
62	C	0.015043	5.749194	0.040261
63	C	0.225222	5.045825	-1.151834
64	C	0.218924	3.649333	-1.137781
65	O	-0.000732	-7.105882	0.14585
66	C	-0.214437	-7.869304	-1.031689
67	O	0.000565	7.105893	0.145873
68	C	0.21447	7.869324	-1.031625
69	H	11.600538	-0.126092	-2.110308
70	H	11.605183	0.075088	2.183039
71	H	12.84156	-0.028191	0.035179
72	H	5.212717	3.352411	0.11112
73	H	2.722844	3.331285	0.104169
74	H	2.708032	-3.343469	-0.006275
75	H	5.19749	-3.375632	-0.019546
76	H	8.219522	-0.7507	2.666061
77	H	9.580681	0.100681	3.414506
78	H	8.293981	1.016337	2.612184
79	H	9.57343	-0.142813	-3.337694
80	H	8.284489	-1.053027	-2.532636
81	H	8.21738	0.714355	-2.586404
82	H	-11.600467	0.126077	-2.110399
83	H	-11.605222	-0.075122	2.182947
84	H	-12.841544	0.028161	0.035055
85	H	-5.197484	3.37563	-0.019658
86	H	-2.708031	3.343478	-0.006379
87	H	-2.722816	-3.331279	0.104141
88	H	-5.212691	-3.352411	0.111155
89	H	-8.219567	0.75067	2.666054
90	H	-9.580751	-0.100701	3.414466
91	H	-8.294037	-1.016366	2.612177
92	H	-9.573327	0.14281	-3.337732
93	H	-8.284407	1.053023	-2.53264
94	H	-8.217295	-0.714358	-2.586409
95	H	0.37062	-3.114969	2.158275
96	H	0.358246	-5.606831	2.147184
97	H	-0.390906	-5.567183	-2.086837
98	H	-0.383443	-3.106632	-2.064111
99	H	-0.371026	3.114955	2.158205
100	H	-0.358736	5.606812	2.147133
101	H	0.39122	5.567224	-2.086747

102	H	0.383835	3.10667	-2.064039
103	H	-0.163704	-8.914426	-0.723397
104	H	-1.200736	-7.665518	-1.468153
105	H	0.561083	-7.672971	-1.783236
106	H	0.163643	8.914443	-0.72334
107	H	1.20086	7.665571	-1.467901
108	H	-0.5609	7.672966	-1.78332

The model structure of **L4**

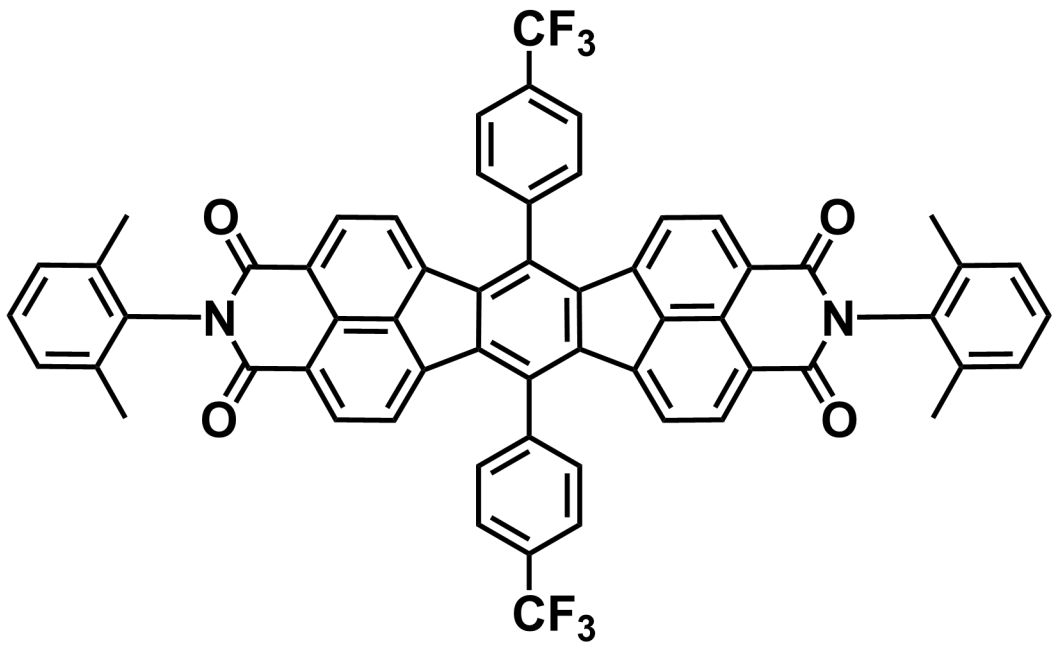

106 atoms

Total energy: -3316.798812 (Hartree)

Table S11. Computational atomic coordinates of ground-state **L4** structure calculated at the DFT//B3LYP-D3(BJ)/6-31G(d) level.

#	Atom	Coordinate		
		X	Y	Z
1	C	11.06135	0.092152	1.206855
2	C	9.663794	0.088284	1.226394
3	C	8.993859	0.030053	0.000273
4	C	9.664367	-0.023704	-1.22574
5	C	11.061915	-0.01819	-1.205976
6	C	11.75626	0.039315	0.000495
7	N	7.546181	0.025196	0.000154
8	C	6.917261	-1.242402	-0.002082
9	C	5.437523	-1.231207	-0.00147
10	C	4.772081	0.015814	-0.000185
11	C	5.429141	1.26725	0.0014
12	C	6.908766	1.288446	0.002301
13	C	4.687176	-2.401143	-0.00257
14	C	3.266543	-2.388387	-0.002262
15	C	2.601974	-1.169613	-0.001103
16	C	3.383116	0.011177	-0.000335
17	C	3.250522	2.409927	0.002175
18	C	4.670997	2.432147	0.002655

19	C	2.594129	1.186726	0.000553
20	C	1.192474	0.720867	-0.000021
21	C	1.197231	-0.713035	-0.000444
22	C	0.004756	-1.444274	-0.000007
23	C	-0.004736	1.444316	0.000374
24	C	8.897409	-0.089138	-2.520873
25	C	8.8962	0.148471	2.521405
26	O	7.563787	2.317687	0.006111
27	O	7.579297	-2.267108	-0.005735
28	C	0.009899	-2.93279	-0.000258
29	C	-0.009881	2.932831	0.000514
30	C	-11.061663	-0.09697	1.205648
31	C	-9.664111	-0.09316	1.225531
32	C	-8.993875	-0.030106	-0.000203
33	C	-9.664127	0.02846	-1.226151
34	C	-11.061679	0.022866	-1.206715
35	C	-11.756305	-0.03939	-0.000645
36	N	-7.546193	-0.025229	0.000029
37	C	-6.90873	-1.288471	0.002048
38	C	-5.429108	-1.267251	0.001438
39	C	-4.772059	-0.015811	0.000476
40	C	-5.437522	1.231194	-0.000825
41	C	-6.917257	1.242377	-0.001832
42	C	-4.670952	-2.432142	0.002257
43	C	-3.250477	-2.409907	0.001976
44	C	-2.594099	-1.186697	0.001146
45	C	-3.383096	-0.011156	0.000664
46	C	-3.266556	2.388406	-0.001219
47	C	-4.687189	2.401141	-0.001755
48	C	-2.601965	1.169642	0.000105
49	C	-1.197214	0.713076	0.000596
50	C	-1.192452	-0.720825	0.00064
51	C	-8.89697	0.099262	-2.520884
52	C	-8.896904	-0.1587	2.520511
53	O	-7.579268	2.267099	-0.005609
54	O	-7.563701	-2.317744	0.005602
55	C	0.001743	3.640698	-1.207598
56	C	-0.002029	5.033257	-1.209844
57	C	-0.016307	5.72931	0.000778
58	C	-0.026381	5.032967	1.211283
59	C	-0.022639	3.640413	1.208787
60	C	0.004542	-3.640318	-1.208612
61	C	0.008302	-5.032876	-1.211229

62	C	0.016324	-5.729268	-0.000744
63	C	0.020142	-5.033263	1.209992
64	C	0.016389	-3.640709	1.207868
65	C	-0.082918	7.231988	0.000343
66	F	0.524385	7.759902	-1.084218
67	F	0.503941	7.759586	1.096259
68	F	-1.363243	7.669118	-0.011582
69	C	0.082938	-7.231946	-0.001251
70	F	-0.518796	-7.759563	-1.089054
71	F	-0.509533	-7.759843	1.0915
72	F	1.363307	-7.669072	-0.006729
73	H	11.603849	0.137054	2.147247
74	H	11.604855	-0.059495	-2.146278
75	H	12.842637	0.042948	0.000584
76	H	5.218016	-3.347499	-0.003651
77	H	2.730088	-3.330229	-0.002965
78	H	2.70765	3.348089	0.003107
79	H	5.195674	3.381935	0.004074
80	H	8.227731	0.771763	-2.630647
81	H	9.579326	-0.096892	-3.375721
82	H	8.283657	-0.996142	-2.570115
83	H	9.577912	0.160853	3.376362
84	H	8.27626	1.051263	2.570571
85	H	8.232401	-0.716986	2.631048
86	H	-11.60437	-0.145582	2.145735
87	H	-11.6044	0.067867	-2.146974
88	H	-12.842682	-0.043031	-0.000819
89	H	-5.19562	-3.381935	0.003093
90	H	-2.707593	-3.348063	0.002445
91	H	-2.730119	3.330257	-0.001892
92	H	-5.218041	3.34749	-0.002965
93	H	-8.225169	-0.75968	-2.632957
94	H	-9.578676	0.107838	-3.375892
95	H	-8.285411	1.007893	-2.567317
96	H	-9.57881	-0.171974	3.375298
97	H	-8.279105	-1.063092	2.56714
98	H	-8.231082	0.704856	2.632799
99	H	0.016694	3.094231	-2.145371
100	H	0.01329	5.579495	-2.146603
101	H	-0.029716	5.578985	2.148293
102	H	-0.026736	3.093724	2.146541
103	H	-0.005517	-3.093587	-2.146297
104	H	-0.002238	-5.578852	-2.148206

105	H	0.018698	-5.579544	2.146854
106	H	0.015596	-3.09428	2.145782

10. References

[1] X. Yang, Y. Yan, W. Zeng, Y. Song, W. Li, L. Zhao, Y. Zhao, H. Chen, Y. Liu, *Chem. Commun.* **2021**, *57*, 7822-7825.

[2] X. Wang, H. Wang, Q. Jiang, Y. I. Lee, S. Feng, H. G. Liu, *Spectrochim. Acta, Part A.* **2018**, *188*, 202-207.

[3] M. A. Salykina, A. S. Bunev, N. A. Lozinskaya, S. E. Sosonyuk, *Tetrahedron* **2025**, *185*, 134824.

[4] Y. Chen, P. Lan, M. G. Banwell, *Org. Lett.* **2022**, *24*, 2931-2934.

[5] J. Robert A. Pascal, William D. McMillan, Donna Van Engen, and Robert Gregory Eason, *J. Am. Chem. Soc.* **1987**, *109*, 4660-4665.

[6] O. V. Dolomanov, L. J. Bourhis, R. J. Gildea, J. A. K. Howard, H. Puschmann, *J. Appl. Cryst.* **2009**, *42*, 339-341.

[7] G. Sheldrick, *Acta Crystallogr., Sect. A* **2008**, *64*, 112-122.

[8] G. Sheldrick, *Acta Crystallogr., Sect. A* **2015**, *71*, 3-8.

[9] M. J. Frisch. et. al. Gaussian 16, Revision B.01;Gaussian, Inc., Wallingford CT, **2016**.

[10] S. Grimme, *J. Comput. Chem.* **2006**, *27*, 1787-1799.

[11] Y. Zhao, D. G. Truhlar, *Theor. Chem. Acc.* **2007**, *120*, 215-241.

[12] B. M. J. Tomasi, R. Cammi., *Chem. Rev.* **2005**, *105*, 2999-3094.

[13] T. Lu, F. Chen, *J. Comput. Chem.* **2012**, *33*, 580-592.

[14] R. Gershoni-Poranne, A. Stanger, *Chemistry* **2014**, *20*, 5673-5688.

[15] A. Stanger, *Chemphyschem* **2023**, *24*, e202300080.

[16] K. H. D. Geuenich, F. Köhler, R. Herges, *Chem. Rev.* **2005**, *105*, 3758-3772.

[17] M. Sheik-Bahae, A. A. Said, E. W. Van Stryland, *Opt. Lett.* **1989**, *14*, 955-957.

[18] M. Sheik-Bahae, A. A. Said, T. H. Wei, D. J. Hagan and E. W. Van Stryland, *IEEE J. Quantum Electron.* **1990**, *26*, 760-769.
